# *Elp1* facilitates RAD51-mediated homologous recombination repair via translational regulation

**DOI:** 10.1186/s12929-021-00773-z

**Published:** 2021-11-24

**Authors:** Wei-Ting Chen, Huan-Yi Tseng, Chung-Lin Jiang, Chih-Ying Lee, Peter Chi, Liuh-Yow Chen, Kai-Yin Lo, I-Ching Wang, Fu-Jung Lin

**Affiliations:** 1 Department of Biochemical Science and Technology, National Taiwan University, No.1, Sec.4, Roosevelt Rd., Taipei, 10617 Taiwan; 2 Institute of Biochemical Sciences, National Taiwan University, Taipei, 10617 Taiwan; 3 Institute of Biological Chemistry, Academia Sinica, Taipei, 11529 Taiwan; 4 Institute of Molecular Biology, Academia Sinica, Taipei, 11529 Taiwan; 5 Department of Agricultural Chemistry, National Taiwan University, Taipei, 10617 Taiwan; 6 Institute of Biotechnology, National Tsing Hua University, Hsinchu, 30013 Taiwan; 7 Research Center for Development Biology and Regenerative Medicine, National Taiwan University, Taipei, 10617 Taiwan

**Keywords:** *Elp1*, RAD51, Homologous recombination, DNA damage, Translational regulation

## Abstract

**Background:**

RAD51-dependent homologous recombination (HR) is one of the most important pathways for repairing DNA double-strand breaks (DSBs), and its regulation is crucial to maintain genome integrity. *Elp1* gene encodes IKAP/ELP1, a core subunit of the Elongator complex, which has been implicated in translational regulation. However, how ELP1 contributes to genome maintenance is unclear.

**Methods:**

To investigate the function of *Elp1*, *Elp1*-deficient mouse embryonic fibroblasts (MEFs) were generated. Metaphase chromosome spreading, immunofluorescence, and comet assays were used to access chromosome abnormalities and DSB formation. Functional roles of *Elp1* in MEFs were evaluated by cell viability, colony forming capacity, and apoptosis assays. HR-dependent DNA repair was assessed by reporter assay, immunofluorescence, and western blot. Polysome profiling was used to evaluate translational efficiency. Differentially expressed proteins and signaling pathways were identified using a label-free liquid chromatography–tandem mass spectrometry (LC–MS/MS) proteomics approach.

**Results:**

Here, we report that *Elp1* depletion enhanced genomic instability, manifested as chromosome breakage and genotoxic stress-induced genomic DNA fragmentation upon ionizing radiation (IR) exposure. *Elp1*-deficient cells were hypersensitive to DNA damage and exhibited impaired cell proliferation and defective HR repair. Moreover, *Elp1* depletion reduced the formation of IR-induced RAD51 foci and decreased RAD51 protein levels. Polysome profiling analysis revealed that ELP1 regulated RAD51 expression by promoting its translation in response to DNA damage. Notably, the requirement for ELP1 in DSB repair could be partially rescued in *Elp1*-deficient cells by reintroducing RAD51, suggesting that *Elp1*-mediated HR-directed repair of DSBs is RAD51-dependent. Finally, using proteome analyses, we identified several proteins involved in cancer pathways and DNA damage responses as being differentially expressed upon *Elp1* depletion.

**Conclusions:**

Our study uncovered a molecular mechanism underlying *Elp1*-mediated regulation of HR activity and provides a novel link between translational regulation and genome stability.

**Supplementary Information:**

The online version contains supplementary material available at 10.1186/s12929-021-00773-z.

## Background

Genomic instability is a common feature of most cancer cells and it is considered an additive impact of DNA damage, impaired DNA repair, and a failure to block cell cycle progression before the damaged DNA has been passed on to daughter cells. Maintenance of genomic integrity and the high fidelity of DNA damage repair mechanisms are fundamental processes for ensuring proper cell growth, development, and survival.

DNA double-strand breaks (DSBs), the most deleterious type of DNA damage, can alter genomic stability and lead to cell death. In mammalian cells, DSBs are induced by exposure to various external hazards, such as UV, ionizing radiation (IR), and interstrand crosslinking agents. DSBs also arise normally during DNA replication and other cellular process, such as V(D)J recombination, meiotic DNA crossover formation, and upon attack by endogenous reactive oxygen species [1]. To prevent accumulation of DNA lesions, a DNA damage response (DDR) can be activated, or genes involved in promoting apoptosis or senescence are induced. The two predominant mechanisms for repairing DSBs are homologous recombination (HR) and non-homologous end-joining (NHEJ) [2, 3]. In general, upon detecting DNA damage, a group of DDR factors is rapidly recruited to the damaged sites, which together activate cell cycle checkpoints and promote DNA repair pathways. Defects in the DNA damage repair response can result in cell death and a number of diseases.

HR is critical for error-free repair of broken chromosomes, such as collapsed replication forks and DNA interstrand crosslinks. It requires the presence of a homologous DNA region as a template for accurate repair. An early step of HR repair is DNA end resection, which generates a single-stranded DNA (ssDNA) tail to initiate the recombination process. Upon recruitment of HR components, the recombinase RAD51 forms a nucleoprotein filament and catalyzes DNA strand exchange with an undamaged sister chromatid, with this latter serving as the DNA template for high-fidelity repairs [4–6]. RAD51-containing core protein complexes in conjunction with context-specific factors are needed for a number of interrelated HR pathways. As RAD51 is the major strand transfer protein in HR-mediated DNA repair, a reduction in the formation of RAD51 foci at DSBs implies impaired recruitment of repair proteins and diminished HR-dependent DSB repair.

Elongator is a highly conserved multisubunit complex that consists of two copies of each of its six subunits (Elp1–6). Every subunit is functionally essential in vivo as deletion of any of them leads to the loss of complex integrity and generates almost identical pleiotropic phenotypes [7]. ELP1 (also known as IKAP and encoded by *Elp1/Ikbkap* gene) is a scaffold protein of Elongator. ELP1 has been shown to play a key role not only in transcription, but also in translation [7, 8]. Mutations in the human *IKBKAP/ELP1* gene, resulting in severely reduced IKAP/ELP1 protein levels in the nervous system, cause the autosomal-recessive neurodevelopmental disorder—familial dysautonomia (FD) [9, 10]. Mice lacking *Elp1* exhibit developmental delay and early embryonic lethality [11]. Peripheral neural-specific deletion of *Elp1* in mice results in increased apoptosis of neural progenitors and peripheral neurons [12]. Recently, germline loss-of-function *IKBKAP/ELP1* variants were identified in patients with pediatric Sonic Hedgehog medulloblastomas, highlighting the role of Elongator and its subunits in tumor development [13]. Apart from its cell autonomous role in neurogenesis, several lines of evidence support a role for ELP1 in suppressing genomic instability. *Elp1-*deficient mouse spermatocytes exhibit defects in DSB repair [14]. In yeast, Elongator mutants are hypersensitive to the DNA damage reagents methyl methanesulfonate (MMS) and hydroxyurea [15]. Although the phenotypes arising from *Elp1* deficiency in mice and yeasts often reflect impaired genome stability, a role for ELP1 in maintaining genomic stability has not yet been systematically established.

Here, we show definitively that *Elp1* plays a role in genome maintenance. Deletion of *Elp1* results in genotoxic agent-induced chromosomal instability. *Elp1-*deficient mouse embryonic fibroblasts (MEFs) were deficient in HR repair and hypersensitive to IR and chemicals that induce DSBs, even culminating in cell death. *Elp1* facilitated HR-directed DSB repair, at least in part, by enhancing *Rad51* translational efficiency and by promoting its expression. Finally, by means of a mass spectrometry (MS)-based proteomics approach and bioinformatics tools, we show that differentially expressed proteins in *Elp1-*deficient MEFs were most significantly enriched in pathways related to cancer, apoptosis, and DNA repair. Overall, our study uncovers a crucial role for *Elp1* in genome integrity.

## Materials and methods

### Generation of *Elp1*^*flox/flox*^ MEFs and cell culture

*Elp1*^*flox/flox*^ mice [14] were provided by Yi Zhang (Harvard Medical School, MA, USA). *Elp1*^*flox/flox*^ MEFs were isolated from embryonic day (E) 13.5 *Elp1*^*flox/flox*^ embryos according to standard protocols. In brief, after removing the head and innards of embryos, the remaining tissue was minced and then incubated overnight with 0.25% trypsin/EDTA at 4 °C. The supernatant was extracted and supplemented with complete medium. After cell dissociation, the cellular suspension was transferred to 10 cm plates. Once the cells had grown to confluency, they were collected and stored in liquid nitrogen (passage 1 MEFs). These primary MEFs were cultured in Dulbecco’s Modified Eagle’s Medium (DMEM) containing 10% fetal bovine serum (HyClone Laboratories, Logan, UT, USA), 100 U/mL penicillin/streptomycin (HyClone Laboratories), 0.1 mM non-essential amino acids (HyClone Laboratories), and 50 µM β-mercaptoethanol (Sigma-Aldrich, St. Louis, MO, USA). To immortalize primary MEFs, early passage (P2) cells were immortalized via retroviral infections with pBabeSV40LT (Addgene #13970) and then selected with G418 (Life Technologies, Carlsbad, CA, USA) for 7 days. Immortalized MEFs were cultured in the same DMEM medium but without addition of β-mercaptoethanol. Human osteosarcoma U2OS cells containing the DR-GFP or EJ5-GFP reporter system were provided by Jeremy M. Stark (City of Hope Medical Center, CA, USA) [16], and they were cultured in DMEM containing 10% fetal bovine serum, penicillin–streptomycin and sodium pyruvate, and maintained at 37 °C in an incubator at 5% CO_2_, 95% humidity.

### Knockout or knockdown of *Elp1*

To knock out *Elp1* from *Elp1*^*flox/flox*^ MEFs, we used control (pLKO.1) and *Cre*-expressing lentiviruses. The *Cre*-expressing lentiviral vectors were provided by I-Ching Wang (National Tsing Hua University, Taiwan). To knock down *ELP1* from U2OS cells, we used control (shCtrl, pLKO_TRC025), and two independent *ELP1* shRNAs, shELP1-73 (clone ID: TRCN0000037873, target sequence: GCTGTGCTCTTGCTGTTAGAA), and shELP1-69 (clone ID: TRCN0000037869, target sequence: GCGTCAAATATCACGTCATTT) lentiviruses. All viruses were obtained from the RNAi Core of Academia Sinica, Taiwan. Cells were incubated with viral supernatant (M.O.I. = 4) supplemented with 6 µg/mL polybrene (Sigma-Aldrich) for 24 h, followed by puromycin-based selection (InvivoGen, San Diego, CA, USA).

### Transfections and DSB reporter assays

Two days after shRNA virus infection, I-SceI expression plasmids containing a hemagglutinin (HA) tag (pCBASceI, Addgene #26477) or pcDNA3.1 control plasmids were transfected into U2OS cells using Lipofectamine 3000 (Thermo Fisher Scientific, Waltham, MA, USA) according to the manufacturer’s protocol. One day after transfection, cells were harvested and analyzed for the percentage of GFP-positive cells by means of flow cytometry (FACSCanto II, BD Biosciences, San Jose, CA, USA). Data were analyzed in FlowJo software (version 9.4.2; Treestar, Ashland, OR, USA).

### Immunofluorescence staining

Cells grown on coverslips were fixed in 4% paraformaldehyde for 20 min, followed by permeabilization with 0.2% Triton X-100 in PBS for 10 min. After blocking, coverslips were incubated overnight with anti-γH2AX (ab22551, 1:1000, Abcam, Cambridge, UK), anti-ELP1 (ab62498, 1:500, Abcam), anti-RAD51 (PC130, 1:500, Merck Millipore, Burlington, MA, USA) or RPA70 (GTX108749, 1:400, GeneTex, Irvine, CA, USA) antibodies at 4 °C. Following several washes with 0.1% Triton X-100/PBS buffer, coverslips were incubated with Alexa Fluor 488-conjugated anti-mouse or Alexa Fluor 594-conjugated anti-rabbit secondary antibodies (#A11001 or #A11012, 1:500, Invitrogen, Waltham, MA, USA) for 1 h. DAPI was used to stain nuclei. Fluorescence mounting medium (Agilent Dako, Santa Clara, CA, USA) was used to mount coverslips. Images were taken with a Leica TCS SP5 II Confocal microscope (Leica Microsystems, Wetzlar, Germany) or Zeiss AxioImager M2 microscope (Zeiss, Oberkochen, Germany). Images were analyzed using Axiovision or Leica Application Suite X software.

### Metaphase chromosome spread

Cells were incubated with 0.1 μg/mL colcemid (Thermo Fisher Scientific) for 12 h to block cell cycle progression at metaphase. Following trypsinization, cells were washed twice with ice-cold PBS, and pelleted by centrifugation. The cell pellet was treated in hypotonic solution (75 mM KCl) at 37 °C for 20 min and fixed in a fixative with methanol and acetic acid (2:1, v/v) for at least 4 h at − 20 °C. Cells were re-suspended in freshly prepared fixative, and the fixed cells were dropped on clean glass slides and air-dried. The slides were stained with DAPI to visualize chromosomes. Images were taken with a Leica TCS SP5 II Confocal microscope (Leica Microsystems). Standard criteria were used for scoring metaphase cells. At least 250 cells at metaphase were analyzed per sample.

### Analysis of micronuclei and nuclear buds

Cells were infected with control or *Cre*-expressing viruses at D0 and harvested at D5. Cells containing micronuclei and nuclear buds were identified by DAPI staining. Micronuclei were scored when they did not attach to the main nucleus and had no connection to it [17]. Nuclear buds were counted only when they touched the main nucleus by a nucleoplasmic connection that was thinner than the diameter of the bud [18]. Microscopic images were obtained using a Zeiss AxioImager M2 microscope (Zeiss).

### Comet assay

Cells were seeded in 12-well plates and infected with control or *Cre*-expressing viruses for 4 days. Once cells reached 70% confluency at day 4, a total of 0.75 µg plasmid DNA (hRAD51-expressing or control vectors) was transfected into MEFs using Lipofectamine 3000 (Thermo Fisher Scientific). One day after transfection, cells were washed twice with PBS and irradiated with a 4 Gy IR. At day 6, a comet assay using CometSlides™ (Trevigen, Gaitherburg, MD, USA) under neutral conditions was performed according to the manufacturer’s protocol. In brief, cells were mixed with 1% low-melting temperature agarose and spread onto CometSlides™. Slides were immersed in lysis buffer containing 1% Triton X-100 for 40 min at 4 °C, and then subjected to electrophoresis at 30 V for 35 min at 4 °C, followed by staining with EtBr solution (Sigma-Aldrich). Images encompassing > 50 randomly selected cells per sample were taken using a Zeiss AxioImager M2 microscope (Zeiss). The tail moment was computed as the percentage of DNA in the comet tail multiplied by the tail length in individual nuclei using the TriTek CometScore Freeware 1.6.1.13 (TriTek Technologies, Inc., Wilmington, DE, USA).

### Cell functional assays

For detection of apoptotic cells, cells were infected with control or *Cre*-expressing viruses for 5 days and exposed to etoposide (ETO) (Sigma-Aldrich) or irradiation as indicated. Apoptotic cells were stained and quantified using a FITC Annexin V Apoptosis Detection Kit with 7-amino-actinomycin-D (7-AAD; BioLegend Biotechnology, San Diego, CA, USA). For the cell viability assay, 1 × 10^4^ cells were seeded in 6-well plates one day before being infected with control or *Cre*-expressing lentivirus (M.O.I. = 4). Five days after virus infection (day 5), cells were exposed to IR (4 Gy) (IBL 637 Cesium-137 γ-ray machine, Cisbio International, Saclay, France). Cell numbers were counted at days 3, 4, 6, and 9. For clonogenic experiments, 3000 cells were seeded in 6-well plates. Cells were infected with lentivirus, treated with puromycin, and exposed to irradiation as indicated. Cells were allowed to grow and on day 10 were stained with 0.1% crystal violet. Quantitative changes in clonogenicity were determined by extracting the colonies with acetic acid and measuring the absorbance of the extracted dye at 592 nm. For cell cycle analysis, cells were harvested and fixed in 70% ethanol at 4 °C for 24 h. Prior to analysis, the fixed cells were washed with PBS and stained in PBS containing propidium iodide (10 µg/mL) (BioLegend Biotechnology), RNase (10 U/mL), and triton X-100 (0.1% v/v). Analysis was performed using a FACSCalibur flow cytometer (BD Biosciences).

### Quantitative real-time-PCR assay

Total RNAs were extracted from cells using QIAzol (Qiagen, Hilden, Germany). Complementary DNAs were generated by SuperScriptIV reverse transcriptase (Thermo Fisher Scientific). Quantitative RT-PCR analyses were performed with a QuantStudio 3 Real-Time PCR System (Applied Biosystems, Waltham, MA, USA). Data are presented as fold-change in gene expression, normalized to 18S ribosomal RNA. The primer sequences are listed in Additional file 1: Table S1.

### Western blot analysis

Cells were lysed with 1× RIPA buffer (Thermo Fisher Scientific) supplemented with protease and phosphatase inhibitor cocktail (Roche Applied Science, Upper Bavaria, Germany) for 30 min on ice. After centrifugation for 10 min, the supernatant was collected and the protein content of the samples was analyzed by means of BCA Protein Assay (T-Pro Biotechnology, Taipei, Taiwan). Proteins were loaded onto SDS-polyacrylamide gels and blotted onto PVDF membranes (GE Healthcare Life Science, Marlborough, MA, USA). Western blots were performed using primary antibodies directed against ELP1 (ab62498, 1:1500, Abcam), RAD51 (PC130, 1:2000, Merck Millipore), HA (GTX115044, 1:200, GeneTex), p53 (#2524, 1:1000, Cell Signaling Technology, Danvers, MA, USA), phospho-p53 (#9284, 1:1000, Cell Signaling Technology), GAPDH (GTX100118, 1:5000, GeneTex) and β-actin (GTX109639, 1:10000, GeneTex). Horseradish peroxidase-linked anti-mouse or anti-rabbit conjugates were used as secondary antibodies (1:5000, Jackson ImmunoResearch, West Grove, PA, USA). Visualization was carried out by enhanced chemiluminescence development (T-Pro Biotechnology).

### Polysome profiling analysis

At least 3 × 10^6^ cells were lysed with polysome lysis buffer (200 mM NaCl, 15 mM MgCl_2_, 15 mM Tris-HCl pH 7.3, 1% Triton X-100, 0.1 mg/mL cycloheximide) on ice for 10 min and centrifuged at 13,300 rpm for 10 min at 4 °C. The supernatant containing 300 μg RNA was loaded onto the top of a 10–50% sucrose gradient and centrifuged at 40,000 rpm for 2 h at 4 °C using a Beckman L8-60M Ultracentrifuge (Beckman Coulter, Brea, CA, USA). By continuous measurement of absorbance at 254 nm, the gradient was collected into 13 fractions (1 mL/fraction) using a gradient fractionator (BioComp Instruments, Fredericton, Canada). RNAs in each fraction were isolated and analyzed.

### Protein preparation and liquid chromatography–tandem mass spectrometry (LC–MS/MS) analysis

Cells were lysed and proteins were precipitated with acetone. Following BCA-based quantification of protein concentration (T-Pro Biotechnology), extracted protein was reduced in 5 mM dithiothreitol (DTT) and alkylated in 10 mM iodoacetamide. It was further digested with trypsin at 29 °C for 16 h, and the reaction was stopped by adding 10% trifluoroacetic acid (TFA) to a final concentration of 0.5%. Digested peptides were desalted by using C18 before being analyzed by LC–MS/MS using an Orbitrap Fusion Lumos Tribrid quadrupole-ion trap-Orbitrap mass spectrometer (Thermo Fisher Scientific) coupled to a UHPLC system (UltiMate 3000, Thermo Fisher Scientific). Peptide mixtures were loaded onto the analytical column and separated by reversed-phase chromatography using a 25-cm column with an inner diameter of 75 μm, packed with 2 μm C_18_ particles (Acclaim PepMap RSLC; Thermo Fisher Scientific). The protein solution was run with a gradient concentration of acetonitrile ranging from 2 to 40% in 0.1% formic acid in acetonitrile at a flow rate of 300 nL/min for 90 min. Positive ions were generated by electrospray and the Orbitrap was operated in data-dependent acquisition (DDA) mode. A survey scan 350–1750 m/*z* was acquired in the Orbitrap (resolution = 120,000 at 200 m/*z*, with the AGC target of 500,000 ions). High-energy collision activated dissociation (HCD)-MS/MS (resolution of 15,000) was used to fragment multiply-charged ions within a 1.4 Da isolation window at normalized collision energy of 32. An AGC target of 50,000 was set for MS/MS analysis with previously selected ions dynamically excluded for 180 s. Samples were analyzed in duplicate. All MS/MS spectral data were searched against the mouse database from the UniProt website (http://www.uniprot.org) using MASCOT 2.3 (Matrix Science, London, UK) via the Proteome Discoverer (PD) package (version 2.2, Thermo Fisher Scientific). The following parameter settings were used: trypsin cleavage; two missed cleavage sites; modification allowed for cysteine carbamidomethylation and methionine oxidation. Peptide mass tolerance was set to ± 10 ppm, and fragment MS/MS tolerance was set to 0.02 Da, and a 1% false discovery rate (FDR) was accepted. For relative protein quantification across different samples, each protein group is represented by a single master protein and the raw abundance of each protein was normalized by total abundance. At least two independent samples per condition were analyzed.

### Ingenuity pathway analysis

Differentially expressed proteins were analyzed by Ingenuity pathway analysis (IPA, www.ingenuity.com) (Qiagen, Redwood City, CA, USA) to identify canonical pathways, associated diseases, biological functions, and upstream regulators.

### Statistical analysis

Results are presented as mean ± standard error of the mean (SEM). All datasets were tested for normality for Student’s *t*-test, and if the normality test failed, a Mann–Whitney rank-sum test was used instead. Statistical analyses were performed using SigmaPlot 12.5 or Microsoft Excel. *p* values < 0.05 were considered significant. *p < 0.05, **p < 0.01, and ***p < 0.005.

## Results

### *Elp1* prevents genomic instability

**Fig. 1:**
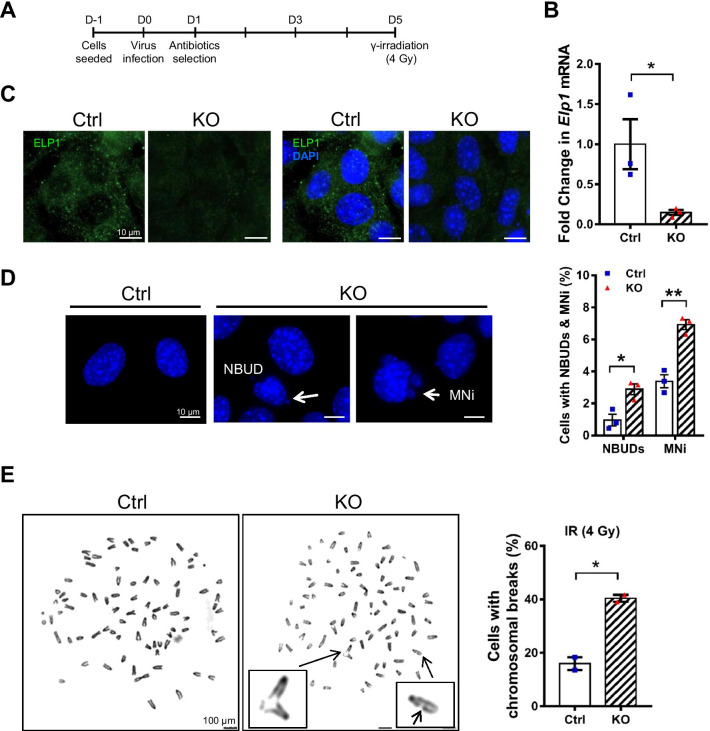
*Elp1* knockout results in chromosomal instability. **A** Schematic of our experimental design. Infected cells were irradiated with a single acute dose of 4 Gy IR at day (D) 5 unless otherwise described. **B** qRT-PCR analysis of the *Elp1* mRNA expression by D5 MEFs. **C** Immunofluorescence for ELP1 (green) in D5 MEFs. Nuclei were counterstained with DAPI (blue). Scale bars, 10 μm. **D** Representative images of nuclear buds (NBUDs), and micronuclei (MNi) in the Ctrl and KO MEFs. Scale bars, 10 μm. Statistical results were obtained from analyzing at least 100 cells in each group. Data represent the average of three independent experiments. **E** Ctrl and KO MEFs were exposed to 4 Gy of IR at D2, and chromosome breaks were counted in a minimum of 100 metaphase cells in each group at D3. Representative images of chromosomal spreads for Ctrl and KO MEFs are shown in the left panel, and quantification of the percentage of cells displaying chromosome breaks is presented in the right panel. Arrows indicate DNA breaks. Data represent the average of two independent experiments. Results are presented as mean ± SEM. Scale bars, 100 μm. *p < 0.05; **p < 0.01 compared to Ctrl cells using Student’s *t*-test

To generate *Elp1* conditional knockout cells, we isolated *Elp1*^*flox/flox*^ MEFs from mice harboring an *Elp1* conditional knockout allele in which two *loxP* sites were inserted on either side of exon 4 (Additional file 1: Fig. S1). Cre recombinase was then introduced by lentiviral infection to generate *Elp1-*deficient (KO) MEFs. Puromycin was added on the next day to enrich for infected cells. Five days after Cre introduction, a low-dose IR treatment (4 Gy) was applied to induce DSBs (Fig. 1A). We found that *Elp1* transcripts were significantly reduced by that time-point, as evidenced by qRT-PCR analysis (Fig. 1B). Immunofluorescence staining also revealed that ELP1 is predominantly expressed in the cytoplasm of control (Ctrl) MEFs, whereas ELP1 signal was marked diminished in KO MEFs (Fig. 1C). Chromosomal instability is usually characterized by a high frequency of nuclear buds (NBUDs) and micronuclei (MNi). We examined the effect of *Elp1* knockout on these biomarkers of chromosomal damage, which revealed higher incidences of NBUD and MNi formation in KO MEFs relative to Ctrl cells (Fig. 1D). To directly assess if *Elp1* deletion increases genomic instability, we performed cytogenetic analysis of chromosomes in MEFs following low-dose IR treatment. *Elp1* transcripts were significantly reduced at day 3 (Additional file 1: Fig. S2). We observed an elevated frequency of chromosomal abnormalities, including at least two-fold increases in chromosomal breakage, in KO MEFs compared to Ctrl cells (Fig. 1E). These results suggest an increased level of genomic instability in KO cells.

### KO MEFs are hypersensitive to IR-induced DNA damage

**Fig. 2:**
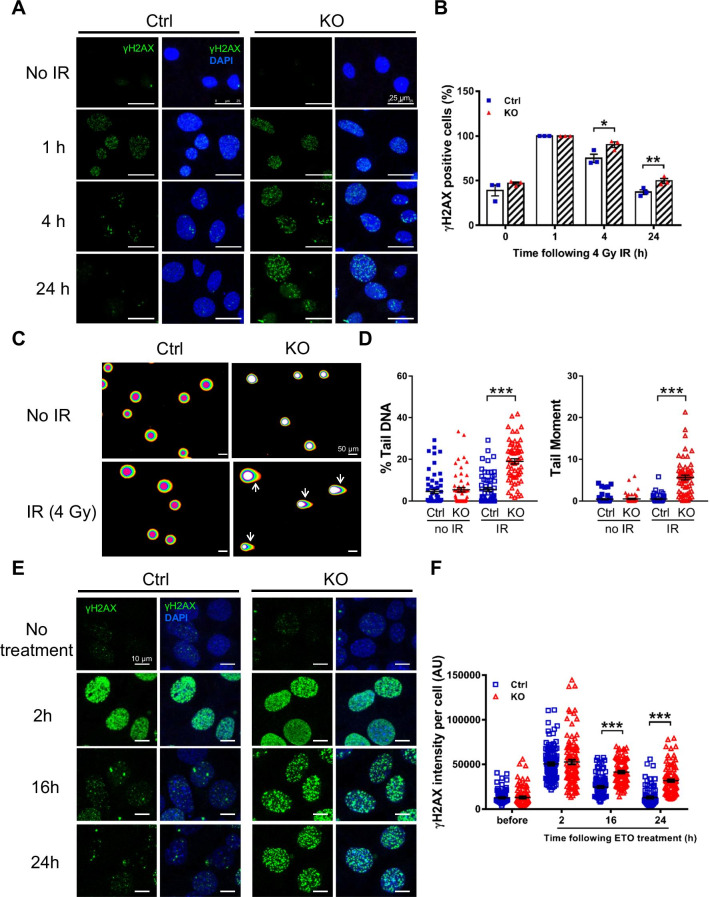
KO MEFs accumulate elevated levels of DNA damage in response to IR and ETO treatment. **A** Representative images of γH2AX foci staining. *Elp1*^*flox/flox*^ MEFs were infected with Ctrl or *Cre*-expressing lentivirus. Cells were either non-irradiated (0 h) or treated with 4 Gy of IR at D5, before being returned to normal culture conditions for a further 1, 4 or 24 h. Cells were then fixed and an immunofluorescence assay was performed using antibodies against γH2AX (green). Nuclei were stained with DAPI (blue). Scale bars, 25 µm. **B** Quantification of the number of cells with more than 10 γH2AX-positive foci per cell. For each time-point, at least 200 cells per group were counted. Results represent the average of three independent experiments. **C** Representative images of cells analyzed by comet assay. Ctrl and KO MEFs were either non-irradiated (0 h) or exposed to 4 Gy of IR, and then 24 h later they were subjected to comet assay. Arrows indicate genomic DNA migrating away from its original location. Scale bars, 50 µm. **D** Quantification of DNA damage was performed by comet assay. We measured the parameters of % tail DNA (left panel) and tail moment (right panel). Data shown are from three independent experiments, and at least 50 comets per group were measured per experiment. **E**, **F** Detection of DNA damage by staining for γH2AX foci. Ctrl and KO MEFs were either untreated (no treatment) or treated with 40 μM of ETO for 2, 16 or 24 h, and then subjected to γH2AX immunofluorescence staining. Representative images are shown in (**E**), and γH2AX intensity per cell is summarized in (**F**). γH2AX intensity per nucleus was analyzed using Image J software. The images of at least 200 cells per group were taken and used to quantify γH2AX intensity. Scale bars, 10 µm. Data are presented as mean ± SEM. *p < 0.05; **p < 0.01; ***p < 0.001 compared to Ctrl cells using Student’s *t*-test

Defects in genome maintenance lead to an accumulation of unrepaired DNA lesions and the activation of the DDR [19]. To establish if *Elp1* deficiency promotes DDR activation, we assayed for formation of phosphorylated histone variant H2AX (γH2AX) foci in KO cells with or without IR (4 Gy), as γH2AX is indicative of unrepaired or irreparably damaged DNA [20]. γH2AX strongly promotes the recruitment of DSB repair proteins, with the process of repair usually being completed within 24 h after DSB induction [21]. We found that the percentage of cells with γH2AX-positive foci was significantly higher in KO cells compared to Ctrl cells at 4 and 24 h following irradiation (Fig. 2A, B), indicating that KO cells are less effective at repairing DSBs. To directly determine the effect of *Elp1* knockout on DNA damage, we performed a comet assay. In the absence of IR treatment, amounts of DSBs were comparable between Ctrl and KO cells (Fig. 2C, D). However, upon IR treatment, we observed a significant increase in DNA migration, manifested as comet tails, for KO MEFs compared to Ctrl cells (Fig. 2C), which was confirmed by statistical analyses of the percentage of tail DNA and the extent of tail moment (Fig. 2D). These results indicate that unrepaired DNA damage following IR was significantly higher in KO cells relative to Ctrl MEFs.

### *Elp1* knockout enhances levels of DNA damage in the presence of genotoxic stress

To further demonstrate DDR activation upon *Elp1* deletion, we treated MEFs with ETO, a compound that targets DNA topoisomerase II and that promotes DSB production. As observed for IR treatment, we observed significantly higher numbers of γH2AX foci in KO MEFs compared to Ctrl MEFs at 16 and 24 h after drug removal (Fig. 2E, F), demonstrating that *Elp1* knockout renders MEFs more susceptible to genotoxic agent-induced DSB accumulation. Numbers of γH2AX foci were comparable in Ctrl and KO MEFs at the earlier time-point of 2 h (Fig. 2E, F), evidencing that ETO treatment had induced similar levels of DNA damage. These results indicate that KO MEFs are hypersensitive to genotoxic stress relative to Ctrl MEFs and further reveal that KO MEFs are less effective in DNA damage repair rather than being more susceptible to DNA damage.

### *Elp1* deficiency and DNA damage together reduce cell division and induce apoptosis

**Fig. 3:**
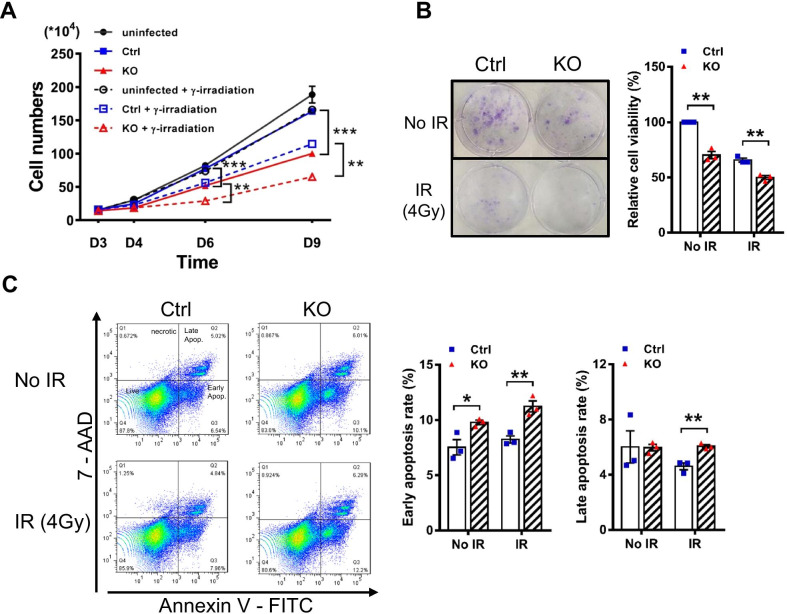
*Elp1* deficiency inhibits cell viability and colony formation, and induces apoptosis in MEFs in response to IR. **A**, **B** Cell numbers and clonogenic assays for Ctrl and KO MEFs that had either been non-radiated (no IR) or exposed to 4 Gy of IR at D5. **A** Cell numbers were counted at the indicated time-points. **B** At D10, cells were stained with crystal violet (left panel), and cell viability was quantified (right panel). **C** Analysis of apoptosis in Ctrl and KO MEFs with or without IR treatment at D10. Cells are classified as “late apoptotic” (top right quadrant), “necrotic” (top left), “live” (bottom left), or “early apoptotic” (bottom right). Representative scatter plots (left panel) and quantitative analysis (right panel) of early apoptotic cells are shown. Results represent the average of three independent experiments. Data are presented as mean ± SEM. *p < 0.05; **p < 0.01 compared to Ctrl cells using Student’s *t*-test

To examine the functional consequences of defective DSB repair in KO cells, we assayed cell viability in the presence or absence of IR. In the absence of IR treatment, we observed a significant decrease in cell division six or nine days after induction of *Elp1* deletion relative to Ctrl cells (Fig. 3A). Moreover, combinatorial *Elp1* knockout and radiation treatment was additive in terms of reducing cell division (Fig. 3A), revealing that *Elp1* knockout alone or in combination with IR treatment was sufficient to compromise cell division. We further analyzed the cell cycle profiles of Ctrl and KO cells using flow cytometry and propidium iodide staining. In the absence of IR treatment, knockout of *Elp1* significantly increased the proportion of cells in the G1 phase and reduced the proportion of cells in the S phase of the cell cycle (Additional file 1: Fig. S3), suggesting G1 cell cycle arrest in KO cells. Next, we employed a colony formation assay to further confirm these results. As shown in Fig. 3B, the colony-forming capacity of KO MEFs was significantly impaired when compared to Ctrl MEFs. As anticipated, combining *Elp1* knockout with IR further reduced cell clonogenicity (Fig. 3B).

To characterize a role for *Elp1* in DNA damage repair, we analyzed apoptotic rates of Ctrl and KO MEFs in response to IR treatment by staining the cells with 7-AAD and Annexin V-FITC. Using flow cytometric analysis, we classified four groups of cells: necrotic (quadrant 1), late apoptotic (quadrant 2), early apoptotic (quadrant 3), and live (quadrant 4). *Elp1* knockout alone or in combination with IR treatment caused a marked increase in the proportion of early and late apoptotic cells relative to Ctrl cells (Fig. 3C). We obtained similar results for cells treated with ETO, with the proportion of late apoptotic cells being significantly elevated in cells lacking *Elp1* upon treatment with 10 μM ETO for 24 h compared to Ctrl cells (Additional file 1: Fig. S4). These data indicate that knockout of *Elp1* results in hypersensitivity to IR- and genotoxic agent-induced DNA damage.

### *Elp1* is required for HR repair

**Fig. 4:**
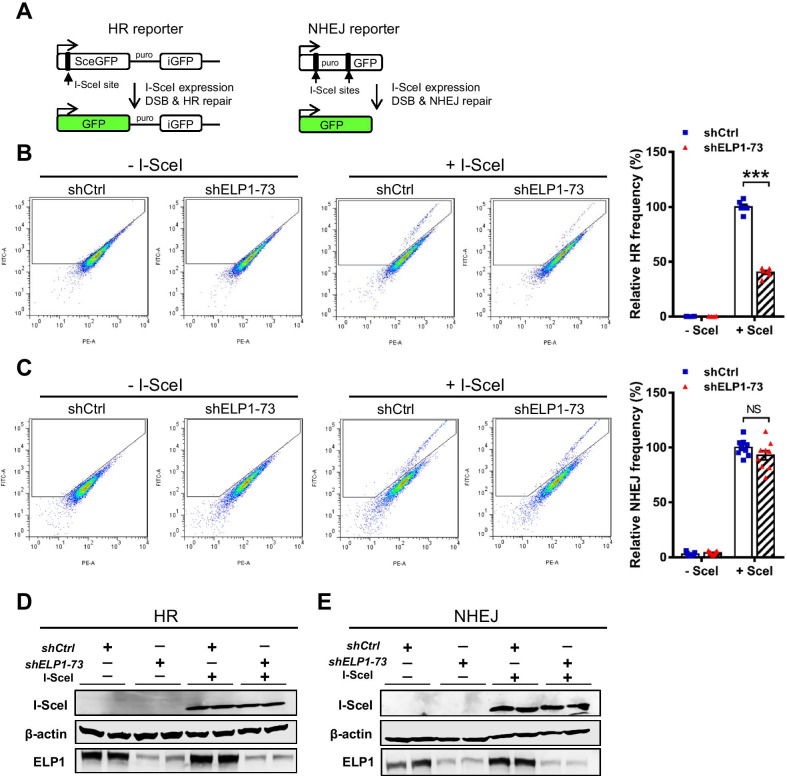
*Elp1* deficiency impairs homology-directed DNA repair. **A** Schematics to the HR and NHEJ reporters. **B**, **C** Determination of relative HR and NHEJ efficiency using the DR-GFP (**B**) or EJ5-GFP (**C**) reporter assay, respectively. U2OS cells were infected with Ctrl or *ELP1* shRNAs for 48 h prior to I-SceI transfection. One day after I-SceI expression, GFP-positive cells were analyzed using flow cytometry. Relative HR or NHEJ frequencies were normalized to the percentage of GFP-positive cells in the Ctrl group. The frequency in the Ctrl group with I-SecI expression was set as 100%. Data are presented as mean ± SEM. **D**, **E** Expression levels of I-SceI, ELP1 and β-actin were examined in DR-GFP (**D**) and EJ5-GFP (**E**) reporter cell lines by immunoblotting. Bars present mean ± SEM. ***p < 0.001 compared to Ctrl cells using Student’s *t*-test. NS, not-significant

Eukaryotic cells repair DSBs mainly via the HR and NHEJ pathways. Next, we investigated a possible role for *Elp1* in DSB repair using U2OS cell lines harboring reporter systems for HR or NHEJ [16]. Relative HR or NHEJ frequency was normalized to the percentage of untreated cells. I-SceI, a yeast homing endonuclease, was used to induce a DSB within an 18 base-pair (bp) sequence, causing overhangs of four nucleotides [22]. U2OS direct repeat green fluorescent protein (DR-GFP) cells that harbor a single copy of a genomically-integrated DR-GFP construct were used to measure homology-directed repair (HDR). DR-GFP includes a GFP expression cassette interrupted by an I-SceI recognition site, followed by an internal GFP (iGFP) fragment (Fig. 4A). Upon DSB induction following I-SceI expression, a functional GFP gene and GFP signals can only be restored by HDR using the downstream iGFP sequence as template. We found that, in the absence of I-SceI expression, GFP signals were barely detected in these U2OS DR-GFP cells treated with control or *ELP1* shRNA (Fig. 4B). In the presence of I-SceI expression, HDR was triggered in cells treated with control shRNA, as reflected by our detection of GFP signals (Fig. 4B and S5A). Conversely, knockdown of *ELP1* significantly decreased the frequency of HDR upon exogenous expression of I-SceI (Fig. 4B and Additional file 1: Fig. S5A), suggesting that the HR repair pathway was suppressed by silencing *ELP1*. In addition, we employed the NHEJ reporter EJ5-GFP, which includes two fragments of a GFP expression cassette interrupted by a *puro* gene that is flanked by two I-SceI recognition sites [23]. GFP signal can only be restored by the NHEJ repair pathway when the *puro* gene is removed by exogenously expressed I-SceI (Fig. 4A) [23]. As for EJ5-GFP, we could barely detect GFP signal in Ctrl or *ELP1*-knockdown cells in the absence of I-SceI induction (Fig. 4C). However, unlike our results for the HR reporter assay, the frequency of NHEJ was not significantly altered by silencing *ELP1* (Fig. 4C and Additional file 1: Fig. S5B). Expression of ELP1, HA-I-SecI, and β-actin in both DR-GFP and EJ5-GFP cell lines was further confirmed by Western blot analysis (Fig. 4D, E, Additional file 1: Fig. S5C, D). These results imply that *ELP1* mainly affects the HR repair pathway elicited by DNA damage.

In response to DNA damage, mammalian cells activate cell cycle checkpoints, recruit DNA repair proteins, and/or trigger apoptosis. ATM and ATR are central to these responses [24]. ATM rapidly reacts to DSBs induced by agents such as IR. At the DNA damage foci, ATM phosphorylates effector molecules, such as p53 [25]. We then analyzed the activation of p53. In the absence of IR treatment, p53 phosphorylation on serine 15 was increased in KO MEFs compared to Ctrl cells, whereas the p53 protein level was not significantly altered between groups (Additional file 1: Figure S6A). Compared to non-irradiated cells, IR induced phosphorylation of p53 at serine 15 in both Ctrl and KO cells, with a significant increase in Ctrl cells. These results suggested that ATM-mediated phosphorylation of p53 was not defective upon *Elp1* deficiency. DNA end resection, an early step of HR repair, produces 3ʹ single-stranded DNA (ssDNA) tails, and replication protein A (RPA) is the main eukaryotic ssDNA-binding protein [26]. We examined whether RPA-ssDNA complex was formed in KO MEFs by measuring RPA foci. Cells showing the high level of foci (> 10 per cell) were evident as early as 1 h after IR treatment and continued to accumulate through 24 h. The proportion of cells with high level of RPA foci was similar between Ctrl and KO MEFs (Additional file 1: Fig. S6B), suggesting that RPA coating was not significantly affected by the loss of *Elp1*.

### *Elp1* regulates the translational efficiency of *Rad51*

**Fig. 5:**
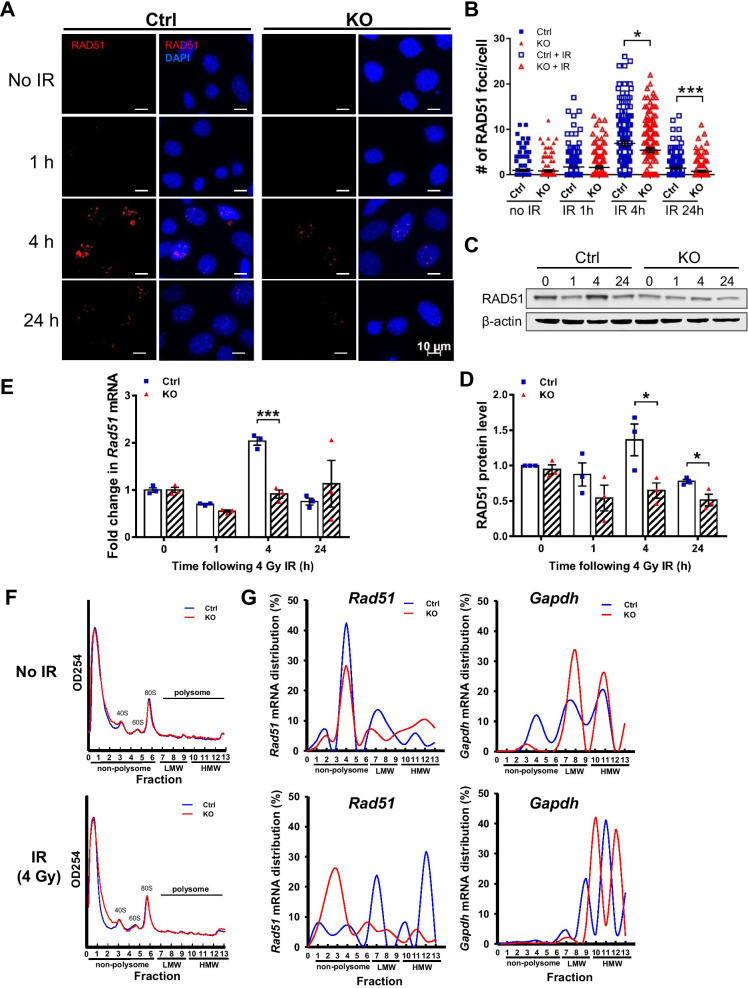
*Elp1* deficiency impairs formation of RAD51 foci by regulating RAD51 protein levels and *Rad51* translation. **A** Representative images of RAD51 foci in Ctrl and KO MEFs without irradiation (0 h) or 1, 4 or 24 h post-irradiation. Scale bars, 10 μm. **B** Quantification of the number of RAD51 foci per cell, with at least 100 cells counted for each independent experiment. **C**, **D** Western blotting analysis (**C**) and quantitative analysis (**D**) of RAD51 protein levels. β-actin was used as a loading control. **E** qRT-PCR analysis of *Rad51* mRNA in Ctrl and KO MEFs that were either non-radiated (0 h) or exposed to 4 Gy of IR. Data are from three independent experiments. Results are presented as means ± SEM. Student’s *t*-test: *p < 0.05; **p < 0.01; ***p < 0.001. **F** Polysomes from Ctrl and KO MEFs without irradiation (0 h) (top panel) or 4 h post-irradiation (4 Gy) (lower panel) were fractionated by sucrose gradient centrifugation for polysome profile analyses. **G** qRT-PCR analysis of *Rad51* and *Gapdh* mRNAs in polysomal fractions from (**F**). The translational activity associated with each fraction is indicated as: non-polysome (not translated); LMW—low-molecular-weight polysomes (moderately translated), or HMW—high-molecular-weight polysomes (actively translated)

Targeting RAD51 to the resected ssDNA and formation of RAD51 filament are critical steps during the HR process. Recruitment of RAD51 to the resected ssDNA can be monitored by the appearance of RAD51 foci, which are considered markers of HR repair [27]. To confirm a role for *Elp1* in promoting HR, we examined the effect of *Elp1* deficiency on formation of RAD51 foci. We performed an immunofluorescence analysis of KO cells using anti-RAD51 antibodies before and after IR treatment. Consistent with the results of our HR reporter assay, KO cells displayed a significant decrease in number of RAD51 foci at 4 and 24 h post-irradiation relative to Ctrl cells (Fig. 5A, B). These results confirm a crucial role for *Elp1* in the HR repair pathway.

Since RAD51 is a critical regulator of HR repair, we wondered if loss of *Elp1* affects RAD51 protein levels. Immunoblot analysis demonstrated that although amounts of RAD51 were not affected upon knockout of *Elp1* from cells in the absence of irradiation treatment (0 h) and 1 h post-irradiation, we observed a reduced amount of RAD51 protein in KO cells relative to Ctrl cells at 4 and 24 h post-irradiation (Fig. 5C, D). Since ELP1 was implicated in transcription elongation [8], we wondered if knockout of *Elp1* affects *Rad51* mRNA levels. In the absence of IR, *Rad51* mRNA levels were comparable between Ctrl and KO cells (Fig. 5E), suggesting that *Rad51* transcription was unlikely to be regulated by ELP1. Intriguingly, Ctrl cells displayed a significant increase in *Rad51* mRNA expression at 4 h post-irradiation relative to KO cells.

Mammalian *Elp1* also functions in tRNA modifications [14], which may impact protein synthesis. To assess if *Elp1* affects translation of *Rad51*, we performed a polysome profiling analysis, enabling us to separate translating mRNAs on a sucrose gradient based on amounts of associated ribosomes. The number of ribosomes associated with an mRNA can be used as a measure of the translation state of that mRNA. Generally, mRNAs found in the non-polysomal fraction of cells are considered non-translated. mRNAs found in the low-molecular-weight (LMW) polysomal fraction present a moderate translational efficiency, whereas those found in the high-molecular-weight (HMW) polysomal fraction exhibit a high translational efficiency [28]. We calculated the translation status of *Rad51* mRNA based on relative levels in each polysomal fraction. We found overall polysome abundance was similar between Ctrl and KO cells in the presence or absence of irradiation treatment (Fig. 5F and Additional file 1: Fig. S7), suggesting that loss of *Elp1* does not globally alter protein translation. Next, we analyzed the translational status of *Rad51* mRNA specifically. In the absence of irradiation, *Elp1* knockout did not significantly change the polysome distribution pattern of *Rad51* and *Gapdh* mRNAs (Fig. 5G). Notably, upon irradiation treatment, knockout of *Elp1* from MEFs caused *Rad51* mRNA to be distributed in the non-polysomal fraction, whereas the majority of *Gapdh* mRNA in both Ctrl and KO cells remained associated with high-molecular-weight (HMW) polysomes (Fig. 5G). We also examined the distribution of *Brca2* and *Xrcc4* mRNAs in the polysomal fractions. Breast cancer 2 (BRCA2) has been shown previously to interact with RAD51 and mediates RAD51-dependent DNA repair [29, 30]. DNA repair protein 4 (Xrcc4) is one of several core proteins involved in the NHEJ pathway for repairing DSBs [31]. The polysome distribution profiles of both *Brca2* and *Xrcc4* mRNAs were not significantly altered between Ctrl and KO cells upon IR treatment (Additional file 1: Fig. S8). These results indicate that *Elp1* knockout decreases the translational efficiency of *Rad51* mRNA upon IR exposure without affecting the translation of some other mRNAs.

### Ectopic RAD51 expression partially rescues impairment of DNA damage repair in *Elp1-*knockout MEFs

**Fig. 6:**
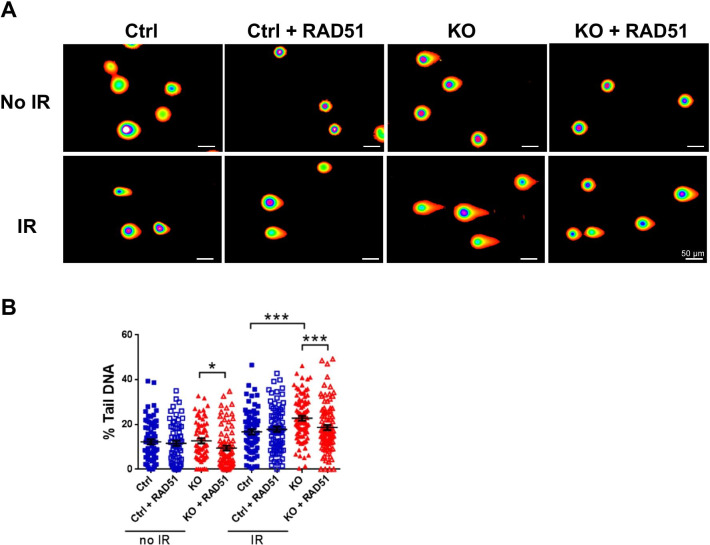
Ectopic expression of RAD51 in KO MEFs abrogates radiation-induced genomic fragmentation. **A** The effects of human *RAD51* overexpression on DNA damage were examined in Ctrl and KO MEFs with or without IR treatment by means of comet assay. Scale bars, 50 µm. **B** Quantification of % tail DNA. Data shown are from three independent experiments, and at least 100 comets per group were measured in each experiment. Data are presented as mean ± SEM. Student’s *t*-test: *p < 0.05; **p < 0.01; ***p < 0.001. Scale bars, 50 µm

If RAD51 mediates the role of *Elp1* in regulating DNA damage repair, we predicted that ectopic expression of RAD51 in KO MEFs might rescue their defects in DSB repair. To explore that possibility, we infected MEFs with control or *Cre* lentiviruses to induce *Elp1* deletion and then transfected the cells with control or human *RAD51*-expressing vectors. Comet assays were then performed in the irradiated or non-irradiated cells. Consistent with our above-described results, we found that deletion of *Elp1* significantly increased DNA tail length upon IR treatment relative to Ctrl cells, indicative of their having more DNA breaks (Fig. 6A, B). More importantly, ectopic expression of RAD51 in KO MEFs partially rescued IR-induced genomic DNA fragmentation (Fig. 6A, B), suggesting that *Elp1* mediates DSB repair at least in part by modulating the expression of RAD51. Western blot analysis confirmed the ectopic expression of RAD51 in MEFs, and we found no influence of ectopic RAD51 expression on ELP1 levels (Additional file 1: Fig. S9).

### Important signaling pathways are associated with *Elp1* deficiency

To elucidate the mechanisms of *Elp1-*mediated IR-induced DSB repair in MEFs, we conducted comprehensive profiling on the proteins isolated from the Ctrl and KO MEFs treated with or without IR (0 or 4 h, respectively). We identified a total of 5753 proteins by LC–MS/MS. Normalized abundance values of the housekeeping protein GAPDH were comparable among groups (Additional file 1: Fig. S10). The normalized abundance value of β-actin was slightly higher in KO than Ctrl MEFs (Additional file 1: Fig. S10). Our proteomic profiling analysis revealed a total of 901 or 569 significant changes in proteins abundance (fold-change ≤ 0.67 or ≥ 1.5) between Ctrl and KO cells at the 0 time-point or between Ctrl and KO cells at the 4 h time-point, respectively, representing a 10–16% alteration from the total identified proteome.

**Fig. 7:**
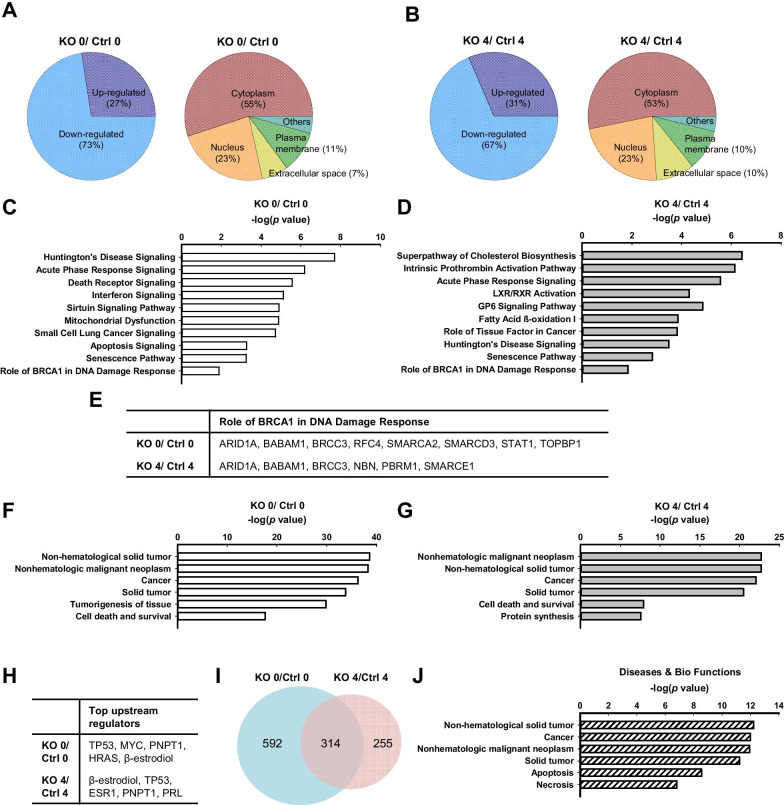
Mass spectrometry proteomics of differentially expressed proteins from Ctrl and KO MEFs. **A**, **B** Pie charts of the expression profiles (KO/Ctrl) (left panel) and subcellular distributions (right panel) of identified proteins from cells without IR treatment (Ctrl 0 and KO 0) (**A**) and 4 h post-IR exposure (Ctrl 4 and KO 4) (**B**). Up-regulated, KO/Ctrl fold-change ≥ 1.5 (*p* < 0.05); down-regulated, fold-differences ≤ 0.67 (*p* < 0.05). **C**, **D** Ingenuity Pathway Analysis (IPA) showing ten of the top most interesting canonical pathways significantly modulated by differentially expressed proteins in KO cells compared to Ctrl cells (*p* < 0.05) without IR treatment (**C**) and 4 h post-IR exposure (**D**). The bar represents − log (*p*-value) for each pathway. **E** Differentially expressed proteins identified in the canonical pathway of “role of BRCA1 in DNA damage response” from the two sets of cells (irradiated and non-irradiated). **F**, **G** IPA showing the top six diseases and biological functions identified for differentially expressed proteins in KO cells compared to Ctrl cells without IR treatment (**F**) and 4 h after IR exposure (**G**). **H** IPA showing the top upstream regulators from the two sets of cells (irradiated and non-irradiated). **I** Venn diagram showing the number of proteins identified from the two sets of cells (irradiated and non-irradiated) by LC–MS/MS, as well as the number of shared proteins. **J** IPA showing the top six diseases and biological functions of overlapping proteins identified between the two sets of cells (irradiated and non-irradiated)

We further examined the differentially expressed proteins according to biological processes using Ingenuity software for IPA. Of the 901 proteins identified from non-irradiated MEFs (0 h time-point), 247 were up-regulated (27%) and 654 were down-regulated (73%) (Fig. 7A). The cell localizations of these 901 proteins were primarily linked to cytoplasm (n = 496, 55%), nucleus (n = 211, 23%), plasma membrane (n = 95, 11%), and extracellular space (n = 60, 7%) (Fig. 7A). Of the 569 differentially expressed proteins in irradiated cells (4 h time-point), 179 were up-regulated (31%) and 390 were down-regulated (69%) (Fig. 7B), and their principle localization were in cytoplasm (n = 303, 53%), nucleus (n = 130, 23%), plasma membrane (n = 59, 10%), and extracellular space (n = 55, 10%) (Fig. 7B).

IPA also revealed several important cancer-related signaling pathways that are differentially impacted in irradiated and non-irradiated cells. Our interested top ten common canonical pathways associated with *Elp1* deficiency include Huntington’s disease signaling, acute phase response signaling, senescence pathway, and role of BRCA1 in DNA damage response (Fig. 7C). These same four pathways were also identified in KO MEFs subjected to IR treatment (Fig. 7D). Although RAD51 did not appear among proteins in the pathway underlying the “role of BRCA1 in DNA damage response”, we did identify ARID1A, BABAM1, and BRCC3 as being differentially expressed in both sets of MEFs (Fig. 7E). Protein expression levels were retrieved from MS results and protein expression ratio (relative to Ctrl) of ARID1A, BABAM1, and BRCC3 in non-irradiated MEFs (0 h time-point) was 2.7, 0.29, and 0.38, while their ratio in irradiated cells (4 h time-point) was 0.56, 1.56, and 0.28. We further analyzed their mRNA levels using qRT-PCR. In the presence or absence of IR, *Arid1a*, *Babam1*, and *Brcc3* mRNA levels were not significantly altered between Ctrl and KO cells, indicating that their transcription was unlikely to be regulated by ELP1 (Additional file 1: Fig. S11A). Moreover, in the absence of irradiation, knockout of *Elp1* from MEFs caused more *Babam1* and *Brcc3* mRNA to be distributed in the LMW and non-polysomal fractions (Additional file 1: Fig. S11B). In response to IR treatment, similar result on *Arid1a* mRNA distribution was obtained, whereas comparable or more *Babam1* and *Brcc3* mRNA was associated with HMW polysomes (Additional file 1: Fig. S11B). These results suggested that in non-irradiated MEFs, knockout of *Elp1* decreases the translational efficiency of *Babam1* and *Brcc3* mRNA.

We also performed disease and biological function analysis on these proteins, and found that important differentially-expressed proteins associated in common with *Elp1* deficiency and/or IR treatment included non-hematological solid tumor, non-hematologic malignant neoplasm, cancer, and solid tumor (Fig. 7F, G). In particular, “protein synthesis” which encompasses 84 proteins, was resolved as one of the top diseases and functions for differentially expressed proteins in the irradiated cell group (Fig. 7G). Details of these selected common alterations of “disease and function” in the paired sets of MEFs are summarized in Additional file 1: Tables S2, S3. Statistically significant upstream regulators in both sets of MEFs included tumor protein 53 (TP53) and polyribonucleotide nucleotidyltransferase 1 (PNPT1) (Fig. 7H). Between the two paired treatments, a total of 314 proteins were found to be overlapped (Fig. 7I). A total of 314 proteins were shared across the two sets of proteins (i.e. non-irradiated and irradiated) (Fig. 7I), which could be linked to cancer, apoptosis, and necrosis (Fig. 7J). Overall, our findings indicate that cancer pathways are the most common feature associated with *Elp1* deficiency.

## Discussion

Here, we provide multiple lines of evidence demonstrating a critical role for *Elp1* in maintaining genomic stability and facilitating DNA repair. Deletion of *Elp1* enhanced genomic instability, as evidenced by the elevated rate of chromosome breakage upon IR treatment. KO MEFs are hypersensitive to IR and chemicals that induce DSBs. In addition, together with the results of our HR reporter assay, the reduced RAD51 protein levels and a diminished capability to form RAD51 foci of KO MEFs strongly imply that *Elp1* promotes HR-dependent DSB repair.

With respect to the mechanism underlying the stimulatory role of *Elp1* in HR repair, our findings unveil that *Elp1* potentially promotes RAD51 protein levels by controlling its translation, consequently enhancing RAD51 foci formation at DSBs. Ectopic expression of RAD51 in KO MEFs partially inhibited comet tail formation, implying that *Elp1* promotes HR repair in a RAD51-dependent manner. RAD51 plays a central role in HR-mediated DSB repair. Disruption of *Rad51* in mice was shown previously to induce early embryonic lethality [32]. *Rad51*-defective cells were found to exhibit chromosome breaks and cell death [33], suggesting a role for RAD51 in cell survival. Intriguingly, RAD51 is overexpressed in many tumors that present aberrantly increased HR activity [34]. Thus, tight regulation of RAD51 is required to maintain genomic integrity. Our results reveal a novel mechanism by which RAD51 protein levels are regulated in response to DNA damage.

ELP1 was initially purified and described as an RNA polymerase II-associated transcription elongation factor [8]. However, the predominantly cytoplasmic localization of ELP1 has prompted an intense investigation of its functions apart from nuclear transcription. It has been subsequently reported that the Elongator complex is involved in translation due to its crucial involvement in modifications, including a thiol (S^2^) and a methoxycarbonyl-methyl (mcm^5^), at the wobble position (U_34_) of certain tRNAs in fission yeast, nematode, mouse, and human [7, 14, 35]. The mcm^5^S^2^ modification to U_34_ enhances the translational efficiency of the AA-ending codons (i.e., AAA, GAA, CAA) [36, 37], whereas its impact on the translation of the AG-ending codons remains largely unclear. There is now widespread consensus that the Elongator complex has an evolutionarily conserved role in regulating the translation efficiency of a vast number of transcripts [38, 39]. In support of that notion, we showed herein by means of a polysome profiling assay that ELP1 modulates *Rad51* mRNA translation efficiency in response to IR treatment. Using a proteome-wide approach, we identified proteins that are biologically significant in the response to *Elp1* knockout, and those proteins were enriched in several significant pathways such as cancer, apoptosis signaling, protein synthesis, and DNA damage response. We and others’ findings support a critical role for *Elp1* in translational regulation. Studies in fission yeast and mouse have shown that Elongator mutants exhibit reduced levels of proteins encoded by large (≥ 1755 total codons), AA-biased transcripts [38–41]. We then analyzed the codon usage of mouse *Rad51* transcript (NM_011234). Interestingly, we found that mouse *Rad51* transcript encodes 339 amino acids, and is AG-biased (the number of AA-ending codons over the number of AG-ending codons, or the AA:AG ratio = 0.9). Although some studies report that the targets of Elongator are large, and enriched in AA-ending (wobble) codons [38, 39], in agreement with our finding, *elp3* mutant yeast exhibited a small decrease in ribosome density at AG-ending codons [42]. Further studies will be necessary to examine whether and how the loss of Elongator complex affects the pairing between U_34_ and G-ending codons, and whether RAD51 is directly regulated by ELP1.

Another surprising finding that emerged from our study is that not only *Rad51* translation but also *Rad51* transcription was considerably affected by the combination of *Elp1* knockout plus IR treatment. Knockout of *Elp1* did not affect *Rad51* transcription in the absence of IR. In contrast, a robust increase in *Rad51* mRNA was observed in Ctrl cells, but not in KO cells upon IR treatment, suggesting that other ELP1-dependent factor(s) promote *Rad51* transcription. Previous studies have shown that *Rad51* transcription is positively regulated by the transcription factor EGR1 and members of the E2F transcription factor family [43, 44], but it is negatively regulated by p53 and hypoxia [45, 46]. However, neither the EGR1 nor E2F transcription factors were identified as differentially expressed in our KO MEFs. There are two possibilities for that outcome. First, the EGR1 and E2F transcription factors may not be regulated by ELP1. Second, the change in expression levels of these factors is below the detection levels achievable by LC–MS/MS method. It would be interesting to validate the protein levels of the EGR1 and E2F transcription factors using Western blot analysis. Although we cannot exclude the possibility that some nuclear ELP1 directly regulates *Rad51* mRNA expression, our results favor the cytoplasmic localization of ELP1 modulates the translation of certain transcriptional regulators that might control *Rad51* mRNA expression. Moreover, apart from transcriptional and translational controls, we did not rule out the possibility that ELP1 may regulate *Rad51* mRNA stability.

Using polysome profiling analysis, we demonstrated an altered distribution of the majority of *Rad51* mRNA from the non-translated to highly translated fractions in Ctrl MEFs in response to IR treatment. Meanwhile, we also noticed a burst in *Rad51* transcription in irradiated Ctrl MEFs compared to non-irradiated MEFs. It is possible that during this time, newly synthesized *Rad51* transcripts in Ctrl MEFs are modified differently such that they are more stable, more likely to be recognized and picked up by polysomes. Nevertheless, under most conditions, translational efficiency is proportional to the number of ribosomes associated with an mRNA [47]. In the context of total *Rad51* mRNA within the cells, its translation efficiency was reduced in KO MEFs upon IR treatment.

The 314 proteins shared between the two sets of MEFs (irradiated or non-irradiated) were involved in various functions including cancer, cell death and survival, and organismal survival. Strikingly, around 95% (n = 301) of them were mapped to cancer pathways, evidencing a strong link between *Elp1* deficiency and cancer. Unexpectedly, RAD51 was not identified among the differentially expressed proteins between Ctrl and KO cells. There are two possibilities for this outcome, including incomplete *Elp1* knockout and the presence of abundant RAD51 proteins/peptides of similar molecular weight. First, the normalized abundance value of ELP1 was only reduced by 30% in our KO cells (data not shown), potentially causing an undetectable change in RAD51 protein levels. Second, the presence of abundant proteins/peptides of similar molecular weight to RAD51 might have prevented us from detecting RAD51. Even though RAD51 was not identified among the mapped canonical pathways, three proteins (ARID1A, BABAM1, and BRCC3) involved in the pathway for “role of BRCA1 in DNA Damage Response” were shared between the two sets of MEFs. ARID1A (AT rich interactive domain 1A), one of the SWI/SNF chromatin remodeling complexes, facilitates efficient DNA DSB end resection and it sustains ATR-dependent DNA damage signaling [48]. Both BABAM1 (BRISC and BRCA1 A complex member 1, also known as MERIT40) and the deubiquitinylating enzyme BRCC3 (BRCA1/BRCA2-containing complex subunit 3) belong to BRCA1-A complex [49, 50], which mediates DNA repair through HR upon DNA damage. BRCA1 can also form a complex with BRCA2/PALB2/RAD51, which promotes efficient HR-mediated repair [30, 51]. We also showed that in the absence of IR treatment, knockout of *Elp1* did not affect transcription, but decreased the translational efficiency of *Babam1* and *Brcc3* mRNA. Further studies will be necessary to examine whether these proteins are directly or indirectly regulated by *Elp1* at translational and/or post-translational level. Together, it is likely that the dysregulation of ARID1A, BRCC3, BABAM1, and RAD51 in KO MEFs can negatively impact genomic stability.

The disease FD is caused by reduced ELP1 protein levels in neuronal tissues due to a homozygous splice site mutation in the *ELP1* gene, and it is characterized by sympathetic nerve system dysfunction [9, 10]. The neurodegenerative hallmarks of FD as patients age include progressive gait ataxia and death of retinal ganglion cells [52]. Progressive neuronal apoptosis has also been observed in sympathetic and sensory ganglia upon neurons innervating target tissues in a mouse model of FD [53]. In addition, elevated DNA damage and mis-regulation of multiple DNA damage response genes was observed in dorsal root ganglia (DRG) of peripheral nervous system (PNS)-specific *Elp1* knockout embryos [38]. In agreement with these studies, our proteomic results have indicated that the apoptosis pathway and DNA damage response are two of the most affected canonical pathways upon *Elp1* deficiency in MEFs. Our finding regarding to elevated levels of fragmented DNA in KO MEFs relative to Ctrl MEFs upon IR treatment was slightly different from the published data from Goffena et al., who showed that *Elp1* conditional knockout neurons displayed increased levels of DNA damage in the absence of IR treatment [38]. The discrepancy may be due to the cell type difference and/or that the power of our comet assay may not be strong enough to detect the minor differences. DNA damage induces cell cycle arrest and apoptosis. Thus, an accumulation of damaged DNA over time could contribute to impaired cell growth and survival. Our results support *Elp1* playing a general role in the maintenance of genome integrity.

## Conclusions

*Elp1* maintains genomic stability by promoting HR-mediated DNA repair in a RAD51-dependent manner. We believe accumulation of DNA damage as a consequence of defective HR repair is the mechanism underlying increased genomic instability in KO cells.

## Supplementary Information

**Additional file 1.** *Elp1* facilitates RAD51-mediated homologous recombination repair via translational regulation. **Figure S1.** Schematic of the *Elp1* mutant allele. **Figure S2.** qRT-PCR analysis of the *Elp1* mRNA expression by D3 MEFs. **Figure S3.** Knockout of *Elp1* impaired cell cycle. **Figure S4.** Apoptosis analysis of Ctrl and KO MEFs treated with ETO for 24 h via flow cytometry. **Figure S5.** *Elp1* deficiency impairs homology-directed DNA repair. **Figure S6.** The ratio of phospho-p53/p53 protein level and the number of RPA foci were not significantly affected in the absence of *Elp1*. **Figure S7.** Part of polysome profiling of Ctrl and KO MEFs shown in Fig. 5F. **Figure S8.** qRT-PCR analysis of *Brca2* and *Xrcc4* mRNA in polysomal fractions shown in Fig. 5F. **Figure S9.** Detection of RAD51, GAPDH and ELP1 by Western blot of Ctrl and KO MEFs transfected with vector expressing full-length human RAD51. **Figure S10.** Normalized abundance values of GAPDH and β-actin among four groups. **Figure S11.** qRT-PCR analysis of *Arid1a*, *Babam1* and *Brcc3* mRNA in Ctrl and KO MEFs and in polysomal fractions shown in Fig. 5F. **Table S1.** The primer sequences for qRT-PCR. **Table S2.** Ingenuity disease and function analysis of mapped differentially expressed proteins between KO0 and Ctrl0 cells. **Table S3.** Ingenuity disease and function analysis of mapped differentially expressed proteins between KO4 and Ctrl4 cells.

## Data Availability

The MS data obtained in this work have been deposited to the ProteomeXchange Consortium via the PRIDE partner repository with the dataset identifier PXD024628. The rest of data generated in this study are available from corresponding author on reasonable request.

## Abbreviations

DDR: DNA damage response
DMEM: Dulbecco’s Modified Eagle’s Medium
DR-GFP: Direct repeat green fluorescent protein
DSBs: Double-strand breaks
ETO: Etoposide
HDR: Homology-directed repair
HMW: High-molecular-weight
HR: Homologous recombination
IPA: Ingenuity pathway analysis
IR: Ionizing radiation
LC–MS/MS: Liquid chromatography–tandem mass spectrometry
LMW: Low-molecular-weight
MEFs: Mouse embryonic fibroblasts
MNi: Micronuclei
MS: Mass spectrometry
NBUDs: Nuclear buds
NHEJ: Non-homologous end-joining
ssDNA: Single-stranded DNA
TP53: Tumor protein 53
