# Supplementary material for: Elp1 facilitates RAD51-mediated homologous recombination repair via translational regulation
Source: J Biomed Sci. 2021 Nov 24;28:81. doi: 10.1186/s12929-021-00773-z (PMC8613991; doi:10.1186/s12929-021-00773-z)
Supplement: Supplementary file 1 — Additional file 1. Elp1 facilitates RAD51-mediated homologous recombination repair via translational regulation. Figure S1. Schematic of the Elp1 mutant allele. Figure S2. qRT-PCR analysis of the Elp1 mRNA expression by D3 MEFs. Figure S3. Knockout of Elp1 impaired cell cycle. Figure S4. Apoptosis analysis of Ctrl and KO MEFs treated with ETO for 24 h via flow cytometry. Figure S5. Elp1 deficiency impairs homology-directed DNA repair. Figure S6. The ratio of phospho-p53/p53 protein level and the number of RPA foci were not significantly affected in the absence of Elp1. Figure S7. Part of polysome profiling of Ctrl and KO MEFs shown in Fig. 5F. Figure S8. qRT-PCR analysis of Brca2 and Xrcc4 mRNA in polysomal fractions shown in Fig. 5F. Figure S9. Detection of RAD51, GAPDH and ELP1 by Western blot of Ctrl and KO MEFs transfected with vector expressing full-length human RAD51. Figure S10. Normalized abundance values of GAPDH and β-actin among four groups. Figure S11. qRT-PCR analysis of Arid1a, Babam1 and Brcc3 mRNA in Ctrl and KO MEFs and in polysomal fractions shown in Fig. 5F. Table S1. The primer sequences for qRT-PCR. Table S2. Ingenuity disease and function analysis of mapped differentially expressed proteins between KO0 and Ctrl0 cells. Table S3. Ingenuity disease and function analysis of mapped differentially expressed proteins between KO4 and Ctrl4 cells. [file 12929_2021_773_MOESM1_ESM.pdf]

Additional file 1.

Elp1 facilitates RAD51-mediated homologous recombination repair via translational regulation

Wei-Ting Chen[1], Huan-Yi Tseng[1], Chung-Lin Jiang[1], Chih-Ying Lee[2], Peter Chi[2,3], Liuh-Yow Chen[4], Kai-Yin Lo[5], I-Ching Wang[6] and Fu-Jung Lin[1,7#]

[1]Department of Biochemical Science and Technology, National Taiwan University, Taipei, 10617, Taiwan [2]Institute of Biochemical Sciences, National Taiwan University, Taipei, 10617, Taiwan [3]Institute of Biological Chemistry, Academia Sinica, Taipei, 11529, Taiwan [4]Institute of Molecular Biology, Academia Sinica, Taipei, 11529, Taiwan [5]Department of Agricultural Chemistry, National Taiwan University, Taipei, 10617, Taiwan [6]Institute of Biotechnology, National Tsing Hua University, Hsinchu, 30013, Taiwan [7]Research Center for Development Biology and Regenerative Medicine, National Taiwan University, Taipei, 10617, Taiwan

[#]To whom correspondence should be addressed, e-mail: fujlin@ntu.edu.tw (F.-J. Lin)

Additional File 1.

Additional Figures 1-11

Additional Tables 1-3

Figure S1

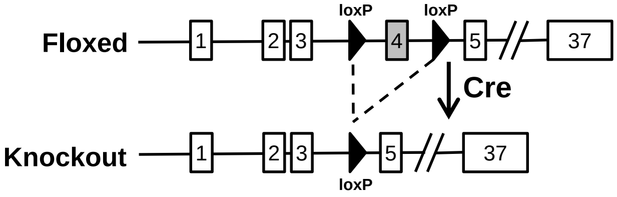

Figure S1. Schematic of the *Elp1* mutant allele.
White boxes with numbers indicate the respective exons of the *Elp1* gene. Exon 4 was flanked by loxP sites, and it was subsequently removed by infecting the *Elp1*$^{flox/flox}$ cells with virus expressing *Cre* recombinase to generate *Elp1*-deficient (KO) cells.

Figure S2

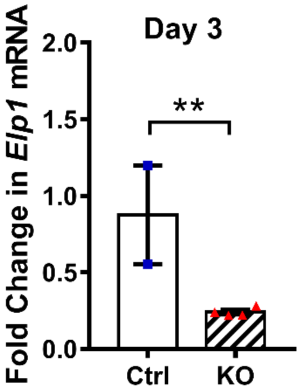

Figure S2. qRT-PCR analysis of the *Elp1* mRNA expression by D3 MEFs.
Ctrl and KO MEFs were exposed to 4 Gy of IR at D2, and *Elp1* mRNA expression was examined at D3. Data represent the average of at least two independent experiments. Results are presented as mean ± SEM. $^{**}p < 0.01$ compared to Ctrl cells using Student's *t*-test.

Figure S3

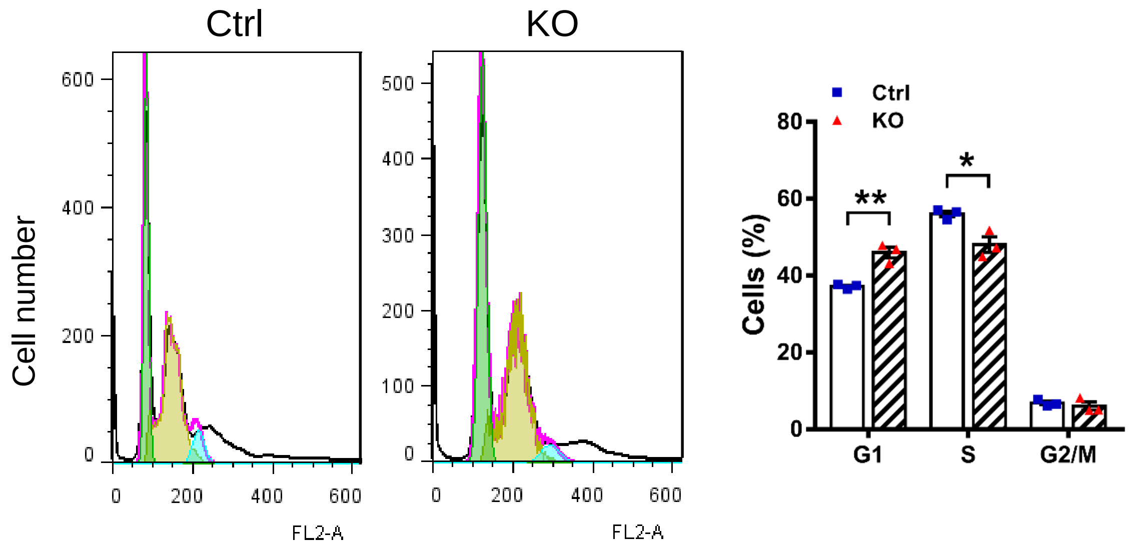

Figure S3. Knockout of *Elp1* impaired cell cycle progression.
(A) The percentage of Ctrl and KO MEFs at different phases of the cell cycle were detected using PI staining. (B) The statistical results of the cycle distribution of Ctrl and KO MEFs. Results shown are the average of three independent experiments. Bars represent mean ± SEM. *$p < 0.05$, **$p < 0.01$ compared to Ctrl cells using Student's *t*-test.

Figure S4

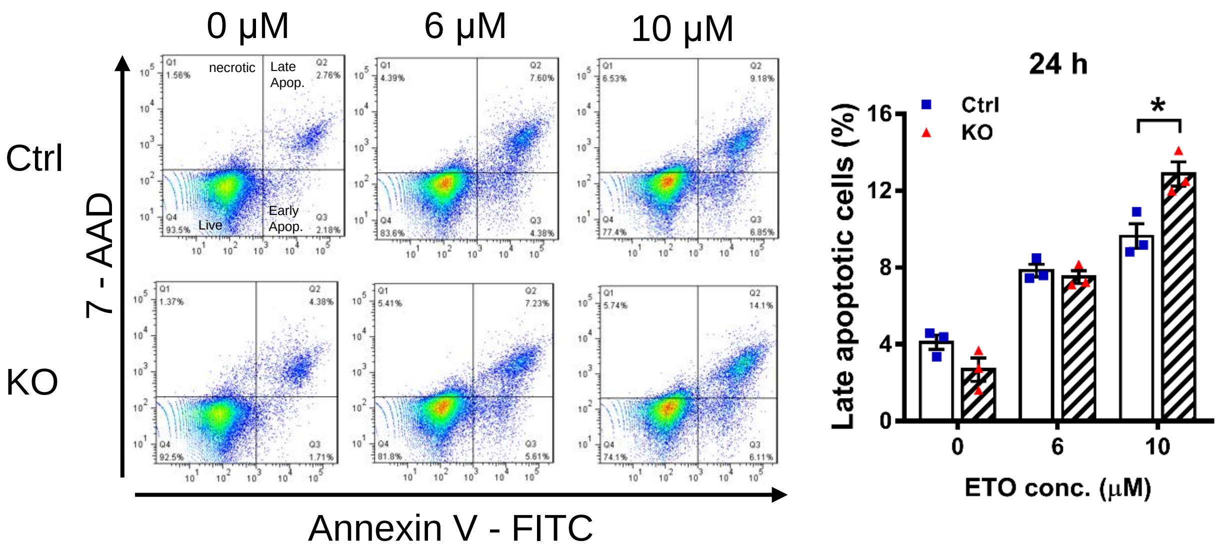

Figure S4. Apoptosis analysis of Ctrl and KO MEFs treated with ETO for 24 h via flow cytometry.
Scatter plots of Annexin V-FITC and 7-AAD stained Ctrl and KO MEFs treated with 0, 6 or 10 μM ETO for 24 h. The cells are classified as "live" (bottom left), "early apoptotic" (bottom right), and "late apoptotic" (top right). Quantitative analysis of late apoptotic cells is shown in the right panel. Results shown are the average of three independent experiments. Bars represent mean ± SEM. *$p < 0.05$ compared to Ctrl cells using Student's *t*-test.

Figure S5

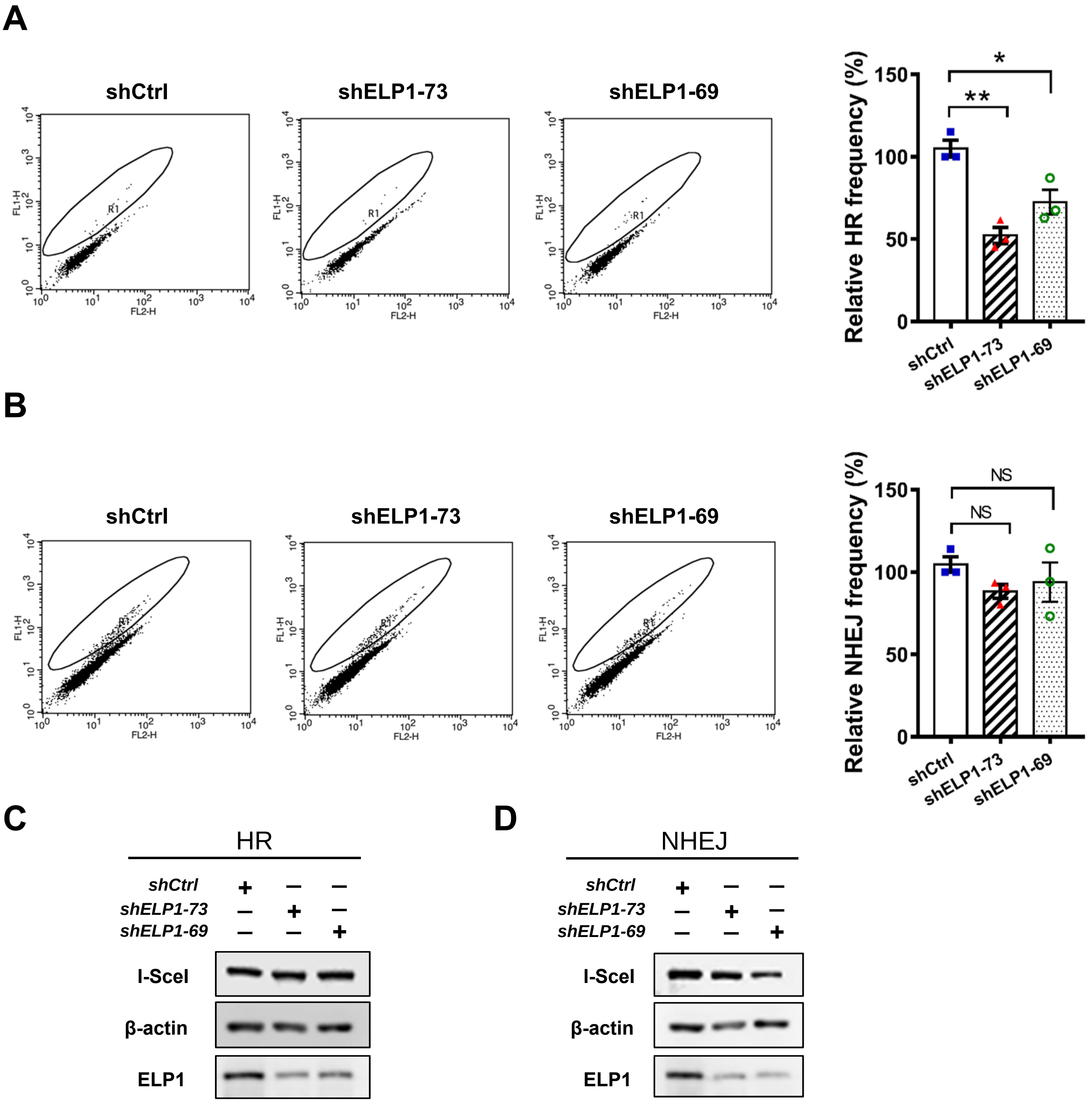

Figure S5. *Elp1* deficiency impairs homology-directed DNA repair.
(**A, B**) Determination of relative HR and NHEJ efficiency using the DR-GFP (**A**) or EJ5-GFP (**B**) reporter assay, respectively. U2OS cells were infected with Ctrl (shCtrl) or two independent *Elp1* shRNAs (shELP1-73, shELP1-69) for 48 h prior to I-SceI transfection. One day after I-SceI expression, GFP-positive cells were analyzed using flow cytometry. Relative HR or NHEJ frequencies were normalized to the percentage of GFP-positive cells in the shCtrl group. The frequency in the shCtrl group was set as 100 %. Data are presented as mean ± SEM. Results shown are the average of three independent experiments. (**C, D**) Expression levels of I-SceI, ELP1 and β-actin were examined in DR-GFP (**C**) and EJ5-GFP (**D**) reporter cell lines by immunoblotting. *p < 0.05, **p < 0.01 compared to shCtrl cells using Student's *t*-test. NS, not-significant.

Figure S6

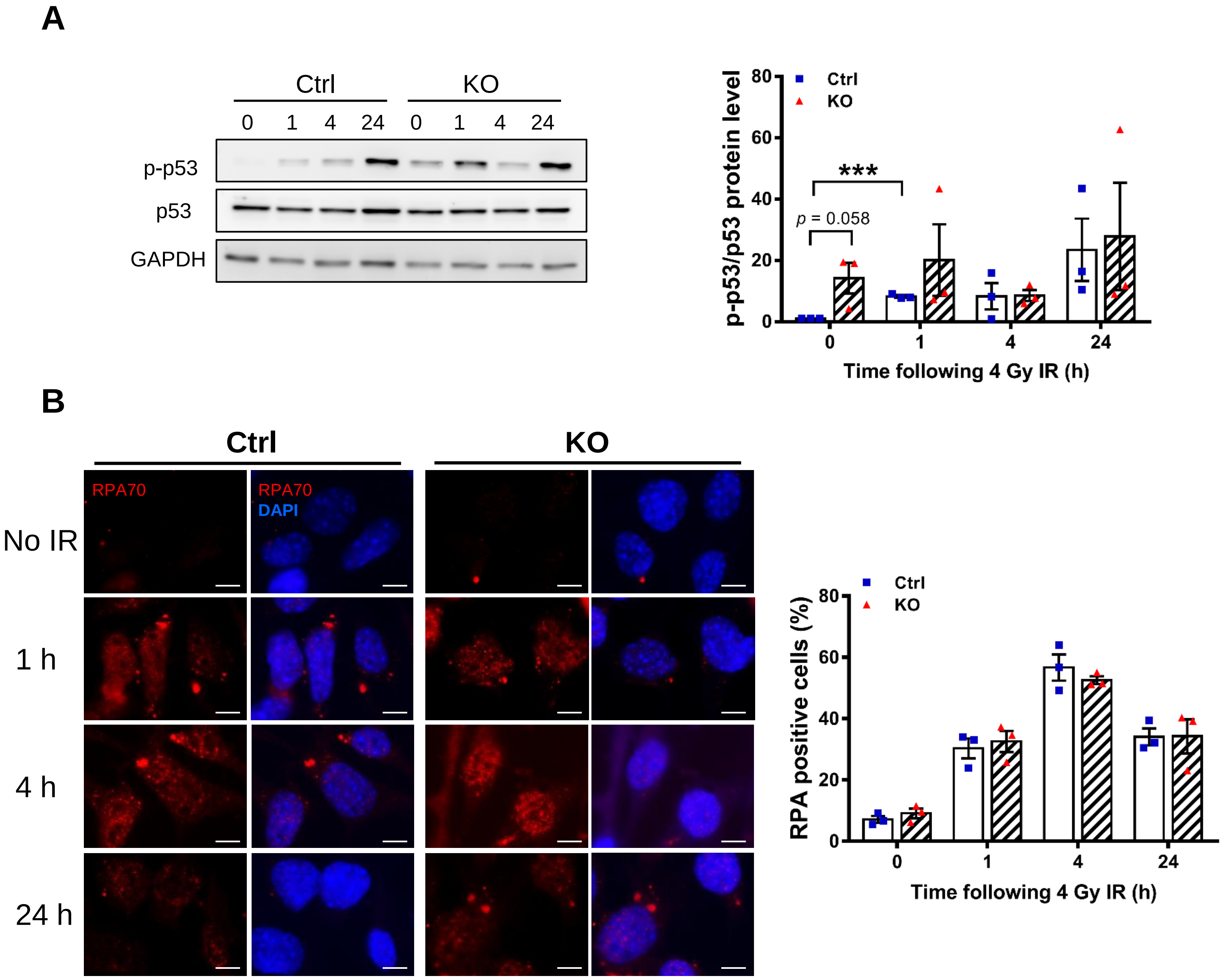

Figure S6. The ratio of phospho-p53/p53 protein level and the number of RPA foci were not significantly affected in the absence of *Elp1*.

(**A**) Western blotting analysis and quantitative analysis of phospho-p53 (Ser15) and p53 expression in Ctrl and KO MEFs. GAPDH served as a loading control. All experiments were repeated at least three times. (**B**) Representative images of RPA foci and quantification of the number of RPA foci per cell in Ctrl and KO MEFs without irradiation (0 h) or 1, 4 or 24 h post-irradiation. At least 200 cells were counted for each independent experiment, and three independent experiments were performed. Results are presented as means ± SEM. Student's *t*-test: ***$p < 0.001$. Scale bars, 10 μm.

Figure S7

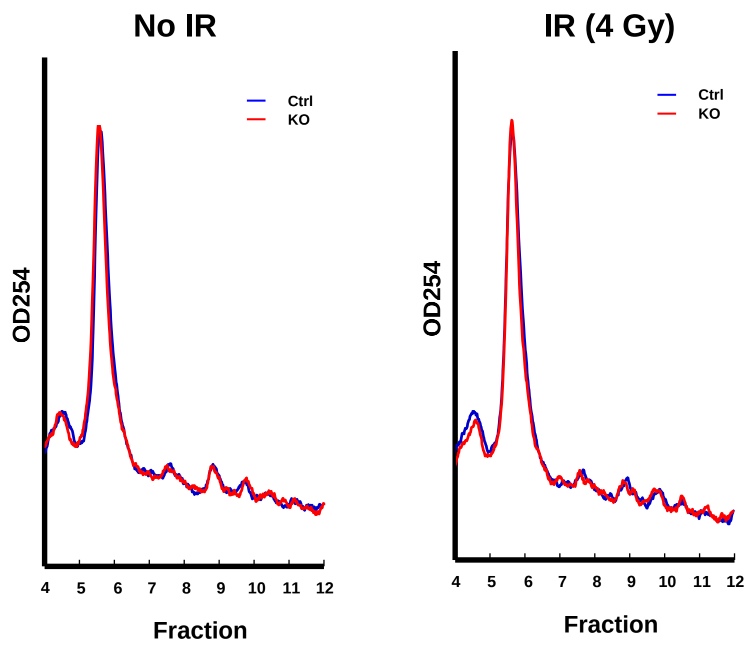

Figure S7. Part of polysome profiling of Ctrl and KO MEFs shown in Figure 5**F**. Polysomes from Ctrl (blue) and KO (red) MEFs without irradiation (0 h) (left panel) or 4 h post-irradiation (4 Gy) (right panel) were fractionated by sucrose gradient centrifugation for polysome profile analyses. Fraction #4 to #12 was shown.

Figure S8

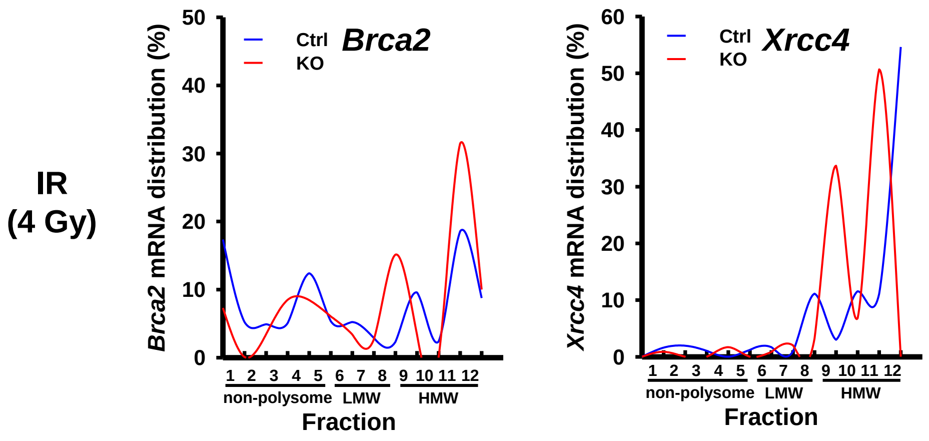

Figure S8. qRT-PCR analysis of *Brca2* and *Xrcc4* mRNA in polysomal fractions shown in Figure 5**F**. The translational activity associated with each fraction is indicated as: non-polysome (not translated); LMW, low-molecular-weight polysomes (moderately translated); and HMW, high-molecular-weight polysomes (actively translated).

Figure S9

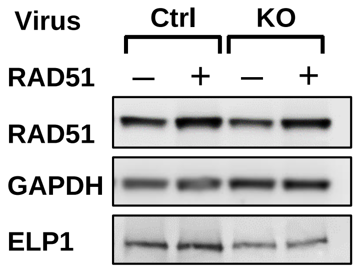

Figure S9. Detection of RAD51, GAPDH and ELP1 by Western blot of Ctrl and KO MEFs transfected with vector expressing full-length human RAD51.

Figure S10

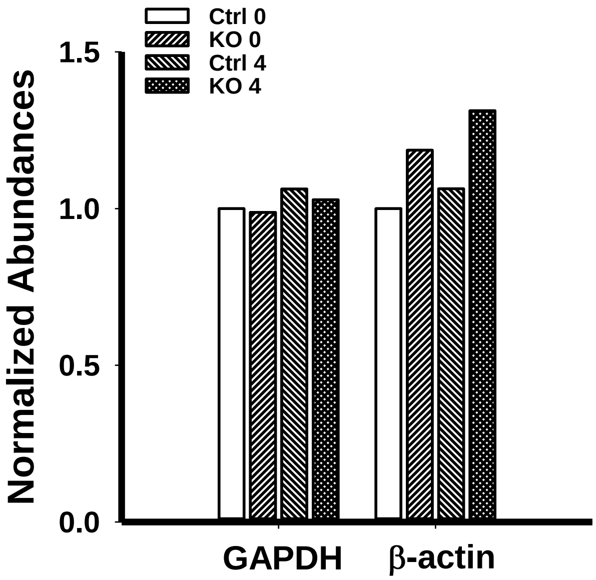

Figure S10. Normalized abundance values of GAPDH and β-actin among four groups.

Figure S11

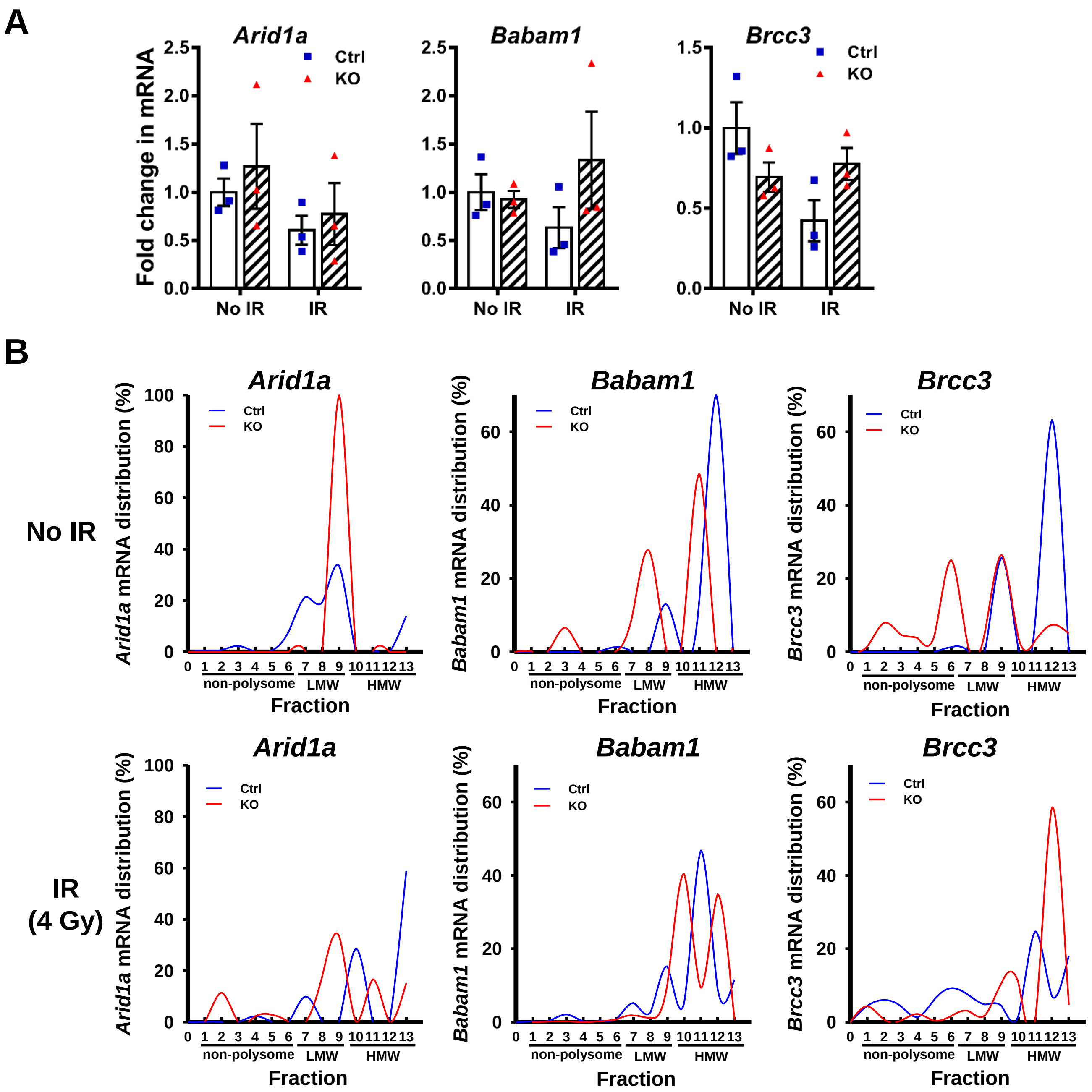

Figure S11. qRT-PCR analysis of *Arid1a*, *Babam1* and *Brcc3* mRNA in Ctrl and KO MEFs and in polysome fractions shown in Figure 5**F**.

(**A**) qRT-PCR analysis of *Arid1a*, *Babam1* and *Brcc3* mRNA in Ctrl and KO MEFs that were either non-radiated (0 h) or exposed to 4 Gy of IR. After 4 h, RNA was isolated from the cells. Data are from at least three independent experiments. Results are presented as means ± SEM. (**B**) qRT-PCR analysis of *Arid1a*, *Babam1* and *Brcc3* mRNAs in polysome fractions from Figure 5**F**. The translational activity associated with each fraction is indicated as: non-polysome (not translated); LMW- low-molecular-weight polysomes (moderately translated), or HMW- high-molecular-weight polysomes (actively translated).

Table S1 The primer sequences for qRT-PCR

Gene	Forward Primer (5'- 3')	Reverse Primer (5'- 3')
mIkbkap	TGCATTGTGGGTATTCAGGA	CAGGACTCCAGCTCATGACA
hIkbkap	ATCATCGAGCCCTGGTTTTAG	ATTGATTCTCAGCTTTCTCATGC
mRad51	GCTCCTTTACCAAGCGTCAG	CCGCCCTGAGTAGTCTGTTC
h18S/ m18S	TCGTATTGCGCCGCTAGAG	TGAAAACATTCTTGGCAAATGC
mGapdh	ATGACCACAGTCCATGCCATC	GAGCTTCCCGTTCAGCTCTG
mBrca2	GCTCCTTCTGCATGTTCTCC	TTGCCAGCACACAGATCTTC
mXrcc4	TGCCTGGACACCATTACAGA	CTCAAATCGGCCTTGAACAT

Table S2. Ingenuity disease and function analysis of mapped differentially expressed proteins between KO0 and Ctrl0 cells

Categories	Diseases or Functions Annotation	p-value	Activation z-sc	Molecules	# Molecules
Cancer,Organismal Injury and Abnormalities	Non-hematological solid tumor	2.28E-39	-0.019	A2M,AAK1,ABCF2,ABCF3,ABHD10,ABHD12,ABRAXAS2,ACAD8,ACADN	860
Cancer,Organismal Injury and Abnormalities	Nonhematologic malignant neoplasm	4.98E-39	-0.024	A2M,AAK1,ABCF2,ABCF3,ABHD10,ABHD12,ABRAXAS2,ACAD8,ACADN	858
Cancer,Organismal Injury and Abnormalities	Cancer	4.78E-37	-0.217	A2M,AAK1,ABCF2,ABCF3,ABHD10,ABHD12,ABRAXAS2,ACAD8,ACADN	866
Cancer,Organismal Injury and Abnormalities	Malignant solid tumor	7.71E-35	0.572	A2M,AAK1,ABCF2,ABCF3,ABHD10,ABHD12,ABRAXAS2,ACAD8,ACADN	861
Cancer,Organismal Injury and Abnormalities	Solid tumor	1.48E-34	0.536	A2M,AAK1,ABCF2,ABCF3,ABHD10,ABHD12,ABRAXAS2,ACAD8,ACADN	863
Cancer,Organismal Injury and Abnormalities	Non-melanoma solid tumor	4.8E-33	-0.144	A2M,AAK1,ABCF2,ABCF3,ABHD10,ABHD12,ABRAXAS2,ACAD8,ACADN	848
Cancer,Organismal Injury and Abnormalities	Extracranial solid tumor	2.11E-32	0.761	A2M,AAK1,ABCF2,ABCF3,ABHD10,ABHD12,ABRAXAS2,ACAD8,ACADN	856
Cancer,Organismal Injury and Abnormalities	Epithelial neoplasm	7.61E-31	-0.353	A2M,AAK1,ABCF2,ABCF3,ABHD10,ABHD12,ABRAXAS2,ACAD8,ACADN	838
Cancer,Organismal Injury and Abnormalities	Tumorigenesis of tissue	1.42E-30	-0.123	A2M,AAK1,ABCF2,ABCF3,ABHD10,ABHD12,ABRAXAS2,ACAD8,ACADN	839
Cancer,Organismal Injury and Abnormalities	Carcinoma	3.65E-30	-0.32	A2M,AAK1,ABCF2,ABCF3,ABHD10,ABHD12,ABRAXAS2,ACAD8,ACADN	836
Cancer,Organismal Injury and Abnormalities	Abdominal carcinoma	3.73E-24	-0.075	A2M,AAK1,ABCF2,ABCF3,ABHD12,ACAD8,ACADM,ACADS,ACAP2,ACA	779
Cancer,Organismal Injury and Abnormalities	Intraabdominal organ tumor	1.7E-23	1.277	A2M,AAK1,ABCF2,ABCF3,ABHD12,ACAD8,ACADM,ACADS,ACAP2,ACA	790
Cancer,Organismal Injury and Abnormalities	Head and neck tumor	4.03E-23	0.624	A2M,AAK1,ABCF2,ABCF3,ABHD12,ABRAXAS2,ACAD8,ACADM,ACADS	758
Cancer,Organismal Injury and Abnormalities	Abdominal neoplasm	3.01E-22	1.227	A2M,AAK1,ABCF2,ABCF3,ABHD12,ACAD8,ACADM,ACADS,ACAP2,ACA	796
Cancer,Organismal Injury and Abnormalities	Abdominal cancer	1.87E-21	1.066	A2M,AAK1,ABCF2,ABCF3,ABHD12,ACAD8,ACADM,ACADS,ACAP2,ACA	788
Cancer,Organismal Injury and Abnormalities	Head and neck cancer	7.76E-21	1.237	A2M,AAK1,ABCF2,ABCF3,ABHD12,ABRAXAS2,ACAD8,ACADM,ACADS	716
Cancer,Organismal Injury and Abnormalities	Abdominal adenocarcinoma	1.07E-20	0.87	A2M,AAK1,ABCF2,ABCF3,ABHD12,ACAD8,ACADM,ACADS,ACAP2,ACA	739
Hereditary Disorder,Neurological Disease,Organismal Injury and Abnormalitie	Familial encephalopathy	1.18E-20	-1.215	A2M,ACADM,ACAT1,ADD3,ADNP,AEBP1,AFG3L2,ALB,AMPD2,ARID1A,	184
Cancer,Organismal Injury and Abnormalities	Formation of solid tumor	1.38E-20	1.202	A2M,AAK1,ABCF2,ABCF3,ABHD12,ABRAXAS2,ACAD8,ACADM,ACADS	739
Cancer,Organismal Injury and Abnormalities	Adenocarcinoma	2.33E-20	1.143	A2M,AAK1,ABCF2,ABCF3,ABHD12,ABRAXAS2,ACAD8,ACADM,ACADS	749
Cancer,Organismal Injury and Abnormalities	Cancer of secretory structure	2.76E-20	0.56	A2M,AAK1,ABCF2,ABCF3,ABHD12,ABRAXAS2,ACAD8,ACADM,ACADS	719
Cancer,Gastrointestinal Disease,Organismal Injury and Abnormalities	Digestive system cancer	2.74E-19	1.227	A2M,AAK1,ABCF2,ABCF3,ABHD12,ACAD8,ACADM,ACADS,ACAP2,ACA	749
Cell Death and Survival	Apoptosis	2.47E-18	1.266	A2M,ABRAXAS2,ADA,ADGRL2,ADI1,ADNP,AGA,AKR1B1,AKT1S1,ALB,	274
Cancer,Gastrointestinal Disease,Organismal Injury and Abnormalities	Digestive organ tumor	2.58E-18	0.755	A2M,AAK1,ABCF2,ABCF3,ABHD12,ACAD8,ACADM,ACADS,ACAP2,ACA	755
Cancer,Organismal Injury and Abnormalities	Head and neck carcinoma	7.1E-18	0.684	A2M,AAK1,ABCF2,ABCF3,ABHD12,ABRAXAS2,ACAD8,ACADM,ACADS	687
Cancer,Organismal Injury and Abnormalities	Neck neoplasm	7.24E-18		A2M,AAK1,ABCF2,ABCF3,ABHD12,ABRAXAS2,ACAD8,ACADM,ACADS	666
Organismal Survival	Organismal death	9.88E-18	6.233	Abcb1b,ACADM,ADA,ADNP,AEBP1,AGA,ANXA1,APAF1,APOA1,APOB,A	247
Organismal Survival	Morbidity or mortality	1.11E-17	6.283	Abcb1b,ACADM,ADA,ADGRE5,ADNP,AEBP1,AGA,ANXA1,APAF1,APOA	249
Cancer,Endocrine System Disorders,Organismal Injury and Abnormalities	Thyroid gland tumor	1.43E-17		A2M,AAK1,ABCF2,ABCF3,ABHD12,ABRAXAS2,ACAD8,ACADM,ACADS	660
Cancer,Endocrine System Disorders,Organismal Injury and Abnormalities	Thyroid cancer	1.67E-17		A2M,AAK1,ABCF2,ABCF3,ABHD12,ABRAXAS2,ACAD8,ACADM,ACADS	658
Cancer,Endocrine System Disorders,Organismal Injury and Abnormalities	Thyroid carcinoma	1.95E-17		A2M,AAK1,ABCF2,ABCF3,ABHD12,ABRAXAS2,ACAD8,ACADM,ACADS	657
Cancer,Endocrine System Disorders,Organismal Injury and Abnormalities	Nonpituitary endocrine tumor	3.04E-17		A2M,AAK1,ABCF2,ABCF3,ABHD12,ABRAXAS2,ACAD8,ACADM,ACADS	664
Cancer,Gastrointestinal Disease,Organismal Injury and Abnormalities	Gastrointestinal tract cancer	9.51E-17	2.03	A2M,AAK1,ABCF2,ABCF3,ABHD12,ACAD8,ACADM,ACADS,ACAP2,ACA	700
Cancer,Endocrine System Disorders,Organismal Injury and Abnormalities	Endocrine gland tumor	1.2E-16	0.989	A2M,AAK1,ABCF2,ABCF3,ABHD12,ABRAXAS2,ACAD8,ACADM,ACADS	682
Cancer,Gastrointestinal Disease,Organismal Injury and Abnormalities	Gastrointestinal carcinoma	1.28E-16	0.275	A2M,AAK1,ABCF2,ABCF3,ABHD12,ACAD8,ACADM,ACAP2,ACAT1,ACB	680
Cell Death and Survival	Necrosis	1.63E-16	0.423	A2M,AAK1,Abcb1b,ACAT1,ADA,ADGRL2,ADI1,ADNP,AGA,AKR1B1,AKT	272
Cancer,Endocrine System Disorders,Organismal Injury and Abnormalities	Endocrine cancer	1.79E-16		A2M,AAK1,ABCF2,ABCF3,ABHD12,ABRAXAS2,ACAD8,ACADM,ACADS	675
Cancer,Gastrointestinal Disease,Organismal Injury and Abnormalities	Gastrointestinal tumor	1.79E-16	0.871	A2M,AAK1,ABCF2,ABCF3,ABHD12,ACAD8,ACADM,ACADS,ACAP2,ACA	701
Cancer,Endocrine System Disorders,Organismal Injury and Abnormalities	Endocrine carcinoma	1.88E-16		A2M,AAK1,ABCF2,ABCF3,ABHD12,ABRAXAS2,ACAD8,ACADM,ACADS	674
Cancer,Gastrointestinal Disease,Organismal Injury and Abnormalities	Large intestine neoplasm	3.14E-15	1.787	A2M,AAK1,ABCF2,ABCF3,ABHD12,ACAD8,ACADM,ACADS,ACAP2,ACA	661
Cancer,Gastrointestinal Disease,Organismal Injury and Abnormalities	Malignant neoplasm of large intestine	3.32E-15	2.925	A2M,AAK1,ABCF2,ABCF3,ABHD12,ACAD8,ACADM,ACADS,ACAP2,ACA	659
Cancer,Gastrointestinal Disease,Organismal Injury and Abnormalities	Large intestine carcinoma	6.64E-15		A2M,AAK1,ABCF2,ABCF3,ABHD12,ACAD8,ACADM,ACAP2,ACAT1,ACB	635
Cell Death and Survival	Cell death of tumor cell lines	1.38E-14	-0.417	ADGRL2,ADI1,ADNP,AGA,AKR1B1,AKT1S1,ALB,APAF1,APOB,ARAF,AF	180
Cancer,Gastrointestinal Disease,Organismal Injury and Abnormalities	Gastrointestinal adenocarcinoma	1.94E-14		A2M,AAK1,ABCF2,ABCF3,ABHD12,ACAD8,ACADM,ACAP2,ACAT1,ACB	647
Developmental Disorder,Neurological Disease	Congenital neurological disorder	3.71E-14	1.238	A2M,ACADM,ALB,AMPD2,APAF1,APOA1,APOB,ARID1A,ARL3,ARSA,AF	106
Cancer,Gastrointestinal Disease,Organismal Injury and Abnormalities	Large intestine adenocarcinoma	7.06E-14		A2M,AAK1,ABCF2,ABCF3,ABHD12,ACAD8,ACADM,ACAP2,ACAT1,ACB	627
Cell Morphology,Cellular Function and Maintenance	Autophagy	7.28E-14	-1.982	ACP2,ALB,APAF1,ARSA,ARSB,ATG4B,ATG7,BCL2L13,CAPN1,COPZ1,C	75
Inflammatory Response	Degranulation	4.57E-13	-0.289	A2M,ADGRE5,AGA,ALB,ANO6,ANXA1,APAF1,APOA1,ARMC8,ARSA,AR	79
Cancer,Organismal Injury and Abnormalities	Incidence of tumor	1.17E-12	0.652	A2M,AAK1,ABCF2,ABCF3,ABRAXAS2,ACAD8,ACADM,ACADS,ACAP2,A	646
Cellular Compromise,Inflammatory Response	Degranulation of cells	1.94E-12	-0.66	A2M,ADGRE5,AGA,ALB,ANO6,ANXA1,APAF1,APOA1,ARMC8,ARSA,AR	77
Developmental Disorder,Neurological Disease,Organismal Injury and Abnorm	Congenital encephalopathy	4.09E-12	-0.065	A2M,ACADM,ALB,AMPD2,APAF1,APOB,ARID1A,ARL3,ARSA,ARSB,ASN	76
Cancer,Organismal Injury and Abnormalities	Frequency of tumor	5.94E-12	0.67	A2M,AAK1,ABCF2,ABCF3,ABRAXAS2,ACAD8,ACADM,ACADS,ACAP2,A	632
Neurological Disease	Motor dysfunction or movement disorde	6.63E-12	2.284	A2M,ABHD12,ACADM,ACAT1,ADD3,AEBP1,AFG3L2,AGA,ALB,ARAF,AF	127
Cell Morphology,Cellular Function and Maintenance	Autophagy of cells	6.87E-12	-1.937	ALB,APAF1,ARSA,ARSB,ATG4B,ATG7,BCL2L13,CAPN1,COPZ1,CRYAE	62
Cancer,Organismal Injury and Abnormalities	Tumorigenesis of epithelial neoplasm	8.55E-12	1.091	A2M,AAK1,ABCF2,ABCF3,ABRAXAS2,ACAD8,ACADM,ACADS,ACAP2,A	626
Infectious Diseases	Viral Infection	1.08E-11	-0.271	ACAP2,ADA,ADGRE5,AFG3L2,AIDA,ALB,ANXA1,APOA1,APOB,AQR,AR	181
Cell Death and Survival	Apoptosis of tumor cell lines	1.35E-11	-0.069	ADGRL2,ADI1,AGA,AKR1B1,AKT1S1,ALB,APAF1,ARAF,ARMC10,ARSB	143
Molecular Transport	Transport of molecule	1.49E-11	0.381	A2M,Abcb1b,ACADS,ACAT1,ACBD3,ACO1,ACSL5,ADA,ADAM9,ADGRL	168
Neurological Disease	Movement Disorders	4.39E-11	1.597	A2M,ABHD12,ACADM,ACAT1,ADD3,AEBP1,AFG3L2,AGA,ALB,ARAF,AF	123
Cell Death and Survival	Cell survival	5.45E-11	-1.704	A2M,Abcb1b,ACSL5,ADNP,AGA,AK3,ALB,ANLN,APAF1,APOB,APPL1,AI	161
Cell Death and Survival	Cell viability	8.19E-11	-1.622	A2M,Abcb1b,ACSL5,ADNP,AGA,AK3,ALB,ANLN,APAF1,APOB,APPL1,AI	154
Developmental Disorder,Hereditary Disorder,Metabolic Disease,Organismal In	Lysosomal storage disease	9.82E-11	2.06	ACP2,AGA,ARSA,ARSB,CAV1,CD47,CTSA,DNAJC5,FUCA1,GBA,GLA,G	23
Protein Synthesis	Metabolism of protein	1.2E-10	0.023	AAK1,ACO1,ACY1,ADAM9,ADI1,AFG3L2,ALB,AMPD2,ANXA1,APAF1,AF	127
Cancer,Organismal Injury and Abnormalities	Malignant genitourinary solid tumor	1.93E-10	-0.009	A2M,AAK1,ABCF2,ABCF3,ABHD10,ACAD8,ACADM,ACADS,ACAP2,ACA	594

Cancer,Organismal Injury and Abnormalities	Development of malignant tumor	2.01E-10	0.917	A2M,AAK1,ABCF2,ABCF3,ABRAXAS2,ACAD8,ACADM,ACADS,ACAP2,A	619
Cancer,Organismal Injury and Abnormalities	Genitourinary tumor	2.12E-10	1.148	A2M,AAK1,ABCF2,ABCF3,ABHD10,ACAD8,ACADM,ACADS,ACAP2,ACA	601
Cancer,Organismal Injury and Abnormalities	Development of carcinoma	2.48E-10	1.597	A2M,AAK1,ABCF2,ABCF3,ABRAXAS2,ACAD8,ACADM,ACADS,ACAP2,A	614
Cellular Compromise,Inflammatory Response	Degranulation of myeloid cells	3.41E-10	0.197	ADGRE5,AGA,ALB,ANO6,ANXA1,APAF1,ARMC8,ARSA,ARSB,ATG7,AT	61
Cancer,Organismal Injury and Abnormalities	Benign Tumors	3.68E-10	-0.173	ACSF2,ADGRE5,AEBP1,ALB,ANXA1,APOA1,ARAF,ASCC3,ATG7,BID,C	106
Cancer,Organismal Injury and Abnormalities	Breast or pancreatic cancer	3.95E-10	0.232	AAK1,ABCF2,ABHD10,ACADS,ACBD3,ACO1,ACP2,ACSF2,ADAM9,ADG	396
Cancer,Neurological Disease,Organismal Injury and Abnormalities	Nervous system neoplasm	4.6E-10	0.153	A2M,AAK1,ABCF2,ABCF3,ABHD12,ACAD8,ACADM,ACADS,ACAT1,ACO	511
Cancer,Organismal Injury and Abnormalities	Benign solid tumor	4.81E-10	-0.173	ACSF2,ADGRE5,AEBP1,ALB,ANXA1,APOA1,ARAF,ASCC3,ATG7,BID,C	105
Organismal Injury and Abnormalities	Benign lesion	5.16E-10	0.398	ACSF2,ADGRE5,AEBP1,ALB,ANXA1,APOA1,ARAF,ASCC3,ATG7,BID,C	118
Cellular Compromise,Inflammatory Response	Degranulation of phagocytes	5.31E-10	-0.214	ADGRE5,AGA,ALB,ANO6,ANXA1,APAF1,ARMC8,ARSA,ARSB,ATG7,AT	60
Carbohydrate Metabolism	Metabolism of carbohydrate	6.04E-10	-0.951	Abcb1b,ABHD12,ACADM,ACP6,AGK,AKR1A1,AKR1B1,ANXA1,AP3B1,A	86
Free Radical Scavenging	Metabolism of reactive oxygen species	6.6E-10	-0.702	Abcb1b,AFG3L2,AKR1B1,ALB,ANXA1,APOA1,ARSA,ATG7,ATP5IF1,ATF	68
Cancer,Organismal Injury and Abnormalities,Reproductive System Disease	Breast cancer	6.97E-10	-0.218	AAK1,ABHD10,ACBD3,ACP2,ADAM9,ADGRL2,ADNP,AEBP1,AIDA,AKR1	245
Free Radical Scavenging	Synthesis of reactive oxygen species	9E-10	-0.873	Abcb1b,AFG3L2,AKR1B1,ALB,ANXA1,APOA1,ARSA,ATG7,ATP5IF1,ATF	65
Cellular Compromise,Inflammatory Response	Degranulation of leukocytes	9.07E-10	-0.113	ADGRE5,AGA,ALB,ANO6,ANXA1,APAF1,ARMC8,ARSA,ARSB,ATG7,AT	62
Cancer,Organismal Injury and Abnormalities,Reproductive System Disease	Mammary tumor	9.24E-10	0.433	AAK1,ABHD10,ACBD3,ACP2,ADAM9,ADGRL2,ADNP,AEBP1,AIDA,AKR1	253
Cancer,Hematological Disease,Immunological Disease,Organismal Injury and	Lymphoma	1.11E-09	0.745	ABRAXAS2,ADA,ADAM9,ALDH9A1,ANXA1,ARID1A,ASCC3,ATG7,B2M,E	147
Digestive System Development and Function,Hepatic System Development a	Morphology of liver	1.21E-09		ACP2,ADA,AEBP1,AIDA,APOA1,BPNT1,Brd4,C3,CAV1,COL3A1,CTHRC	46
Cellular Development,Cellular Growth and Proliferation	Cell proliferation of tumor cell lines	1.21E-09	-1.738	A2M,ACAT1,ACSL5,ADAM9,AGA,AGK,AKT1S1,ANXA1,APAF1,APOB,AF	178
Cellular Compromise,Inflammatory Response	Degranulation of granulocytes	1.21E-09		ADGRE5,AGA,ANO6,ANXA1,APAF1,ARMC8,ARSA,ARSB,ATG7,ATP6AF	51
Hereditary Disorder,Neurological Disease,Organismal Injury and Abnormalitie	Autosomal recessive neurological disor	1.29E-09		ABHD12,ACADM,ADD3,AFG3L2,AMPD2,ARL3,ARSA,ARSB,ASCC3,ASN	79
Cellular Compromise,Inflammatory Response	Degranulation of neutrophils	1.44E-09		ADGRE5,AGA,ANO6,ANXA1,APAF1,ARMC8,ARSA,ARSB,ATG7,ATP6AF	50
Cancer,Organismal Injury and Abnormalities	Breast or colorectal cancer	1.45E-09	1.892	A2M,AAK1,ABCF2,ABCF3,ABHD10,ACADM,ACADS,ACAP2,ACAT1,ACB	491
Cancer,Neurological Disease,Organismal Injury and Abnormalities	Central nervous system solid tumor	1.5E-09	0.365	A2M,AAK1,ABCF2,ABCF3,ABHD12,ACAD8,ACADM,ACADS,ACAT1,ACO	505
Cancer,Organismal Injury and Abnormalities,Reproductive System Disease	Breast or ovarian cancer	1.95E-09	-0.126	AAK1,ABCF2,ABHD10,ACADS,ACBD3,ACP2,ADAM9,ADGRL2,ADNP,AE	315
Cancer,Neurological Disease,Organismal Injury and Abnormalities	Central nervous system cancer	2.16E-09	1.232	A2M,AAK1,ABCF2,ABCF3,ABHD12,ACAD8,ACADM,ACADS,ACAT1,ACO	495
Cardiovascular System Development and Function,Organismal Development	Angiogenesis	3.04E-09	-0.547	ADA,ADAM9,AKR1B1,ANXA1,APOA1,APOB,ATG7,ATP1B3,ATP5IF1,C3	110
Neurological Disease,Organismal Injury and Abnormalities,Psychological Diso	Disorder of basal ganglia	3.33E-09	-0.733	A2M,ACADM,ACAT1,AEBP1,ALB,ARL3,ARSA,ATP5PO,ATP6AP2,ATP6V	87
Cell Death and Survival	Cell death of cervical cancer cell lines	3.45E-09	0.259	ADGRL2,APOB,ARAF,ARSB,ATG7,BID,COL2A1,CTNNB1,DNM1L,EGFR	51
Cellular Movement	Migration of cells	3.61E-09	-3.571	A2M,Abcb1b,ADA,ADAM9,ADGRE5,ADI1,AGK,AKT1S1,ALB,ANO6,ANXA	201
Connective Tissue Development and Function,Tissue Development	Growth of connective tissue	3.82E-09	-1.247	AEBP1,AKR1A1,ALB,ANXA1,ARAF,CAMLG,CASK,CAV1,CCN1,CCN2,C	78
Cellular Movement	Cell movement of tumor cell lines	4.11E-09	-2.678	A2M,ADAM9,ADGRE5,AGK,AKT1S1,ANXA1,APPL1,ARFGEF1,ARID1A,A	117
Cell Cycle	Cell cycle progression	4.44E-09	-1.166	ABRAXAS2,ADGRL2,ANLN,APAF1,ARAF,ARID1A,ARSB,ASNS,Brd4,CA	112
Cancer,Hematological Disease,Immunological Disease,Organismal Injury and	Non-Hodgkin lymphoma	4.52E-09		ADA,ADAM9,ANXA1,ARID1A,ASCC3,B2M,BCAT1,BCL2L13,C3,CAMLG,	127
Carbohydrate Metabolism	Quantity of carbohydrate	4.77E-09	1.356	Abcb1b,ACADM,ACP6,ADA,AIDA,ALB,APAF1,APOA1,ARSB,ATG7,ATP6	67
Cell Death and Survival	Cell viability of tumor cell lines	5.07E-09	-1.714	ACSL5,ADNP,AGA,AK3,ALB,ANLN,ARID1A,ASCC3,ASNS,ATG4B,ATG7	106
Lipid Metabolism,Small Molecule Biochemistry	Catabolism of glycolipid	5.83E-09	-2.605	ARSA,GBA,GLA,GLB1,GM2A,HEXA,HEXB,MAN2B1,NAGA,SMPD4	10
Infectious Diseases	Infection by RNA virus	6.02E-09	-1.607	ADA,ADGRE5,AFG3L2,AIDA,ALB,ANXA1,APOA1,AQR,ARID1A,ASCC3,A	112
Cellular Movement	Cell movement	6.35E-09	-3.403	A2M,Abcb1b,ADA,ADAM9,ADGRE5,ADI1,AGK,AKR1B1,AKT1S1,ALB,AN	218
Cellular Function and Maintenance	Cellular homeostasis	6.44E-09	-1.071	A2M,ACO1,ACP2,ADA,AGA,AKR1B1,ALB,ANXA1,AP3B1,APAF1,APOA1	160
Neurological Disease,Organismal Injury and Abnormalities	Brain lesion	6.83E-09	-0.014	A2M,AAK1,ABCF2,ABCF3,ABHD12,ACAD8,ACADM,ACADS,ACAT1,ACO	487
Cancer,Organismal Injury and Abnormalities	Multiple cancers	7.4E-09	-0.126	AAK1,ABCF2,ABHD10,ACADS,ACBD3,ACP2,ADAM9,ADGRL2,ADNP,AE	321
Cardiovascular System Development and Function	Development of vasculature	7.9E-09	-0.549	ADA,ADAM9,AKR1B1,ANXA1,APOA1,APOB,ATG7,ATP1B3,ATP5IF1,C3	117
Cancer,Organismal Injury and Abnormalities	Genitourinary carcinoma	8.23E-09	-0.449	A2M,AAK1,ABCF2,ABCF3,ABHD10,ACAD8,ACADM,ACADS,ACAP2,ACA	559
Lipid Metabolism,Small Molecule Biochemistry	Catabolism of lipid	1.12E-08	-2.783	ABHD12,ACADS,ACAT1,ACSL5,AP3B1,APOB,ARSA,COMT,ECI2,GBA,G	27
Cancer,Organismal Injury and Abnormalities	Anogenital cancer	1.14E-08	-0.1	A2M,AAK1,ABCF2,ABCF3,ABHD10,ACAD8,ACADM,ACADS,ACAP2,ACE	556
Cancer,Organismal Injury and Abnormalities	Grade 3-4 tumor	1.19E-08		A2M,AAK1,ABCF2,ABCF3,ABHD12,ACAD8,ACADM,ACAT1,ACOT2,ACP	418
Cancer,Neurological Disease,Organismal Injury and Abnormalities	Grade 3-4 glioma cancer	1.23E-08		A2M,AAK1,ABCF2,ABCF3,ABHD12,ACAD8,ACADM,ACAT1,ACOT2,ACP	416
Organismal Development,Organismal Injury and Abnormalities	Abnormal morphology of abdomen	1.32E-08		ADA,AEBP1,AGA,AGK,AIDA,AKR1B1,AMPD2,ARAF,ARSB,B2M,BCAT2,	96
Cardiovascular System Development and Function,Organismal Development	Vasculogenesis	1.43E-08	-1.443	ADA,ADAM9,AKR1B1,ANXA1,APOA1,APOB,ATG7,ATP1B3,ATP5IF1,C3	92
Cellular Assembly and Organization,Cellular Function and Maintenance	Organization of lysosome	1.44E-08		ACP2,AP3B1,ARSB,GAA,GBA,GRN,HEXA,HEXB,MTOR,PPT1,TMEM106	13
Organismal Development	Morphology of body cavity	1.53E-08		ACADM,ACP2,ADA,ADGRE5,AEBP1,AGA,AGK,AIDA,AKR1B1,ALB,AMP	155
Cell Death and Survival,Organismal Injury and Abnormalities	Necrosis of epithelial tissue	1.66E-08	-0.292	AAK1,APAF1,ARID1A,ATG7,ATP1B3,BID,Casp12,CAV1,CD44,COL18A1	72
Cell Morphology,Cellular Function and Maintenance	Macroautophagy	1.81E-08	-1.132	ATG4B,ATG7,EXOC1,EXOC7,GAA,GBA,HDAC6,HMOX1,IKBKG,LAMTO	25
Cellular Growth and Proliferation,Connective Tissue Development and Functio	Proliferation of connective tissue cells	1.9E-08	-1.024	AEBP1,AKR1A1,ALB,CASK,CAV1,CCN1,CCN2,CD44,CDKN2B,CNOT7,C	71
Cancer,Hematological Disease,Immunological Disease,Organismal Injury and	Waldenström macroglobulinemia	2.11E-08		ADAM9,BCAT1,CAMLG,CAV1,COMT,CTBP2,ECI2,EGFR,FADD,GTF2I,H	38
Cancer,Neurological Disease,Organismal Injury and Abnormalities	Brain tumor	2.15E-08	1.195	A2M,AAK1,ABCF2,ABCF3,ABHD12,ACAD8,ACADM,ACADS,ACAT1,ACO	481
Cellular Movement	Migration of tumor cell lines	2.18E-08	-2.883	A2M,ADAM9,ADGRE5,AGK,AKT1S1,ANXA1,APPL1,ARFGEF1,ARID1A,A	101
Cellular Movement	Invasion of tumor cell lines	2.31E-08	-2.819	A2M,ACAT1,ADAM9,ADGRE5,ADI1,AKT1S1,ANXA1,ARID1A,ATG7,CAV	97
Neurological Disease	Neurodegeneration of central nervous s	2.41E-08	1.193	AFG3L2,AGA,ARSA,C3,CAPN1,CAV1,DNAJC5,DNM1L,FUCA1,GBA,GLE	22
Neurological Disease	Neurological signs	3.14E-08	-0.296	A2M,ACADM,ACAT1,AEBP1,APOA1,ARAF,ARL3,ATP5PO,ATP6AP2,B2M	79
Cancer,Gastrointestinal Disease,Organismal Injury and Abnormalities	Development of digestive organ tumor	3.22E-08	0.845	A2M,AAK1,ABCF2,ABCF3,ACADM,ACAP2,ACAT1,ACBD3,ACO1,ACP6,A	470
Lipid Metabolism,Small Molecule Biochemistry	Synthesis of lipid	3.49E-08	-2.052	A2M,Abcb1b,ACAT1,ACBD3,ACP6,ACSL5,ADA,AGK,AKR1B1,ALB,ANXA	89
Cell Death and Survival	Cell death of connective tissue cells	3.53E-08	-0.737	APAF1,ARID1A,ATG7,ATP6AP2,BCL2L13,BID,Casp12,CCN1,CD44,COL	68
Infectious Diseases	Infection of tumor cell lines	3.59E-08	-3.146	ADGRE5,ATG7,ATP5IF1,ATP6AP2,CAPN6,CHCHD2,CNOT7,COG1,COG	60
Digestive System Development and Function,Gastrointestinal Disease,Hepati	Abnormal morphology of liver	3.85E-08		ADA,AEBP1,BPNT1,C3,COL3A1,CTHRC1,CTNNB1,CTSA,EGFR,FADS2	32

Infectious Diseases	Infection of cells	4.01E-08	-2.486	ADGRE5,AFG3L2,AQR,ARID1A,ATG7,ATP5IF1,ATP5PO,ATP6AP2,B2M	83
Cancer,Neurological Disease,Organismal Injury and Abnormalities	Glioma cancer	4.05E-08	0.464	A2M,AAK1,ABCF2,ABCF3,ABHD12,ACAD8,ACADM,ACADS,ACAT1,ACO	481
Cancer,Neurological Disease,Organismal Injury and Abnormalities	Glioma	4.25E-08	0.092	A2M,AAK1,ABCF2,ABCF3,ABHD12,ACAD8,ACADM,ACADS,ACAT1,ACO	489
Developmental Disorder,Hereditary Disorder,Metabolic Disease,Organismal In	Inborn error of lipid metabolism	4.35E-08		ACAD8,ACADM,ACADS,APOB,CPT1A,CPT2,DECR1,GM2A,HADHA,HAD	13
Cancer,Organismal Injury and Abnormalities	Pelvic tumor	4.94E-08	1.821	A2M,AAK1,ABCF2,ABCF3,ACAD8,ACADM,ACADS,ACAP2,ACO1,ACP6,	536
Cellular Assembly and Organization	Organization of organelle	5.63E-08	0.114	ACP2,ADGRL2,ADNP,AFG3L2,AP3B1,ARFGEF1,ARFIP2,ARID1A,ARSB	71
Lipid Metabolism,Small Molecule Biochemistry	Metabolism of membrane lipid derivative	5.81E-08	-2.527	Abcb1b,ABHD12,ACP6,ACSL5,AGK,ANXA1,AP3B1,APOA1,APOB,ARSA	58
Hereditary Disorder,Organismal Injury and Abnormalities,Skeletal and Muscul	Hereditary myopathy	6.67E-08		ACADM,AGK,AMPD2,APOA1,C3,CAPN1,CCN2,CHKB,CNBP,COL1A1,C	71
Cellular Movement	Invasion of cells	6.78E-08	-2.864	A2M,ACAT1,ADAM9,ADGRE5,ADI1,AKT1S1,ANXA1,ARID1A,ATG7,BCA	112
Neurological Disease	Neurodegeneration	7.15E-08	1.69	AFG3L2,AGA,ARSA,ATG7,C3,CAPN1,CASP6,CAV1,COASY,DNAJC5,DN	43
Connective Tissue Disorders,Hereditary Disorder,Organismal Injury and Abno	Hereditary connective tissue disorder	7.19E-08	0.412	AEBP1,AGK,ALB,APOA1,ATP6V1E1,C3,CASK,CAV1,CCN2,CHSY1,CNN	74
Neurological Disease,Organismal Injury and Abnormalities	Neurodegeneration of brain	7.76E-08	1.501	AFG3L2,AGA,ARSA,C3,CAPN1,CAV1,DNAJC5,DNM1L,FUCA1,GBA,GLE	21
Tissue Morphology	Quantity of cells	8.18E-08	-2.723	A2M,Abcb1b,ABRAXAS2,ACAT1,ADA,ADAM9,ADGRE5,ADNP,AGA,AGK	173
Lipid Metabolism,Molecular Transport,Small Molecule Biochemistry	Concentration of phospholipid	8.51E-08	1.02	ABHD12,ACP6,ADA,APAF1,APOA1,BID,CAV1,CDS2,CERS6,ELOVL1,GL	31
Cell Death and Survival	Cell death of breast cancer cell lines	8.59E-08	-1.713	APAF1,ATG7,B2M,BCL2L13,BID,CASP6,CAV1,CCN1,CCN2,CD44,CD47	47
Cancer,Neurological Disease,Organismal Injury and Abnormalities	Astrocytoma	8.97E-08	0.464	A2M,AAK1,ABCF2,ABCF3,ACAT1,ACOT2,ACP2,ACSF2,ACY3,ADA,ADG	384
Cancer,Organismal Injury and Abnormalities	Pelvic cancer	9.07E-08	0.257	A2M,AAK1,ABCF2,ABCF3,ACAD8,ACADM,ACADS,ACAP2,ACO1,ACP6,	529
Gene Expression	Expression of RNA	9.65E-08	-0.551	A2M,ACO1,ADA,ADNP,AEBP1,APOB,ARID1A,BID,BLOC1S2,CAND1,CA	197
Lipid Metabolism,Molecular Transport,Small Molecule Biochemistry	Accumulation of glycosphingolipid	1.04E-07	2.027	ARSA,GBA,GLA,GLB1,GM2A,GULP1,HEXA,HEXB,PHGDH	9
Connective Tissue Development and Function,Skeletal and Muscular System	Quantity of osteoclasts	1.11E-07	0.171	AKR1A1,ATG7,C3,CAPN1,CAV1,CD44,CD47,CHKB,COL1A1,CTNNB1,E	22
Cellular Function and Maintenance	Endocytosis	1.23E-07	-0.685	AAK1,ALB,ANXA1,APOA1,APOB,APPL1,ARAF,ATG7,ATP6V1E1,B2M,C	69
Cell Death and Survival	Apoptosis of cervical cancer cell lines	1.25E-07	0.193	ADGRL2,ARAF,ARSB,BID,CTNNB1,DNM1L,EGFR,EIF2AK2,ELOVL1,EX	40
Cancer,Organismal Injury and Abnormalities,Skeletal and Muscular Disorders	Smooth muscle tumor	1.26E-07		ADGRE5,ANXA1,CAPN6,CCN1,CD44,COL18A1,COL1A1,COL1A2,COL2	42
Cancer,Neurological Disease,Organismal Injury and Abnormalities	High grade astrocytoma	1.3E-07	0.464	A2M,AAK1,ABCF2,ABCF3,ACAT1,ACOT2,ACP2,ACSF2,ACY3,ADA,ADG	381
Cancer,Hematological Disease,Immunological Disease,Organismal Injury and	Tumorigenesis of T lymphocytes	1.36E-07		ADA,ANXA1,ARID1A,B2M,BCL2L13,BID,C3,CAMLG,CCN1,CD47,COL1A	60
Connective Tissue Development and Function,Skeletal and Muscular System	Size of bone	1.4E-07		ACP2,ARSB,ATG7,CAPN1,CAV1,CCN2,CD44,CD47,CHKB,CHSY1,COL1	39
Cancer,Organismal Injury and Abnormalities	Lymphoreticular neoplasm	1.46E-07	1.569	ABRAXAS2,ADA,ADAM9,ALDH9A1,ANXA1,APAF1,APOB,ARAF,ARID1A	225
Cancer,Organismal Injury and Abnormalities,Reproductive System Disease	Breast or gynecological cancer	1.5E-07	-0.05	AAK1,ABCF2,ABHD10,ACAD8,ACADM,ACADS,ACAP2,ACBD3,ACP2,AC	480
Cell Death and Survival	Cell death of colorectal cancer cell lines	1.52E-07	-0.12	APAF1,ATG7,BID,CASP6,CD44,CKAP2,COL18A1,CTBP2,CTNNB1,EGF	42
Neurological Disease	Degeneration of nervous system	1.6E-07	1.998	AFG3L2,AGA,ARSA,ATG7,C3,CAPN1,CASP6,CAV1,DNAJC5,DNM1L,FU	39
Organismal Development	Abnormal morphology of body cavity	1.77E-07		ACADM,ADA,AEBP1,AGA,AGK,AIDA,AKR1B1,AMPD2,ARAF,ARSB,ATP	135
Free Radical Scavenging	Generation of reactive oxygen species	1.79E-07	-0.58	AKR1B1,ALB,APOA1,ATP5IF1,ATP6AP2,CAV1,CCN1,CD47,CRYAB,DN	31
Connective Tissue Development and Function,Tissue Morphology	Quantity of connective tissue	1.85E-07	-1.316	ACADS,AEBP1,AIDA,AKR1A1,APOB,ATG7,C3,CAPN1,CAV1,CCN2,CD4	66
Nervous System Development and Function,Tissue Morphology	Morphology of nervous tissue	1.93E-07		A2M,AGA,APAF1,ARSA,ATG7,B2M,BLOC1S2,C3,Casp12,CAV1,CD47,C	69
Cell Death and Survival	Apoptosis of breast cancer cell lines	1.95E-07	-1.999	APAF1,ATG7,B2M,BCL2L13,BID,CASP6,CAV1,CCN1,CCN2,CD44,CKS1	41
Cell Death and Survival	Apoptosis of fibroblast cell lines	1.95E-07	-0.71	APAF1,ATP6AP2,BCL2L13,BID,CCN1,CRYAB,CTNNB1,CUL7,DAPK3,DN	41
Cellular Development,Cellular Growth and Proliferation,Connective Tissue De	Cell proliferation of fibroblasts	2.1E-07	-1.403	AEBP1,ALB,CAV1,CCN2,CD44,CDKN2B,CTNNB1,DDR2,DNM1L,EGFR,	44
Cancer,Hematological Disease,Immunological Disease,Organismal Injury and	Peripheral T-cell lymphoma	2.31E-07		ARID1A,C3,CCN1,CD47,COL1A2,COL3A1,COL6A1,COL6A2,CTNNB1,E	34
Cancer,Organismal Injury and Abnormalities	Pelvic carcinoma	2.36E-07		A2M,AAK1,ABCF2,ABCF3,ACAD8,ACADM,ACADS,ACAP2,ACP6,ACSF2	507
Free Radical Scavenging	Production of reactive oxygen species	2.51E-07	-0.916	Abcb1b,AFG3L2,AKR1B1,ANXA1,ARSA,ATG7,ATP5IF1,ATP6AP2,BID,C	48
Digestive System Development and Function	Morphology of digestive system	2.52E-07		ACP2,ADA,ADGRE5,AEBP1,AIDA,APAF1,APOA1,ARAF,ARID1A,B2M,B	74
Cellular Assembly and Organization,Cellular Function and Maintenance	Organization of cytoplasm	2.53E-07	-3.007	A2M,ACAP2,ACP2,ADI1,ADNP,AFG3L2,ANXA1,AP3B1,ARFGEF1,ARFIP	147
Hematological System Development and Function,Lymphoid Tissue Structure	Morphology of spleen	2.55E-07		ACP2,ADA,AGK,Brd4,Bst2,C3,CD47,CDKN2B,CKS1B,CTSA,DBNL,DDX5	45
Cancer,Hematological Disease,Organismal Injury and Abnormalities	Hematologic cancer of cells	2.56E-07	0.746	ABRAXAS2,ACP6,ADA,ADAM9,ADD3,ADGRL2,ALB,ALDH9A1,ANXA1,A	189
Cell Death and Survival	Cell death of fibroblast cell lines	2.77E-07	-1.911	APAF1,ARID1A,ATG7,ATP6AP2,BCL2L13,BID,Casp12,CCN1,CRYAB,CT	50
Cellular Function and Maintenance	Internalization of cells	2.85E-07	-0.687	ANXA1,APOA1,ARAF,ATG7,C3,CD44,CD47,CKB,COLEC12,CSK,CTNNE	39
Cancer,Hematological Disease,Immunological Disease,Organismal Injury and	T-cell non-Hodgkin lymphoma	2.93E-07		ADA,ANXA1,ARID1A,B2M,BCL2L13,C3,CAMLG,CCN1,CD47,COL1A1,C	59
Infectious Diseases	Replication of RNA virus	2.93E-07	1.283	ARAF,ATG4B,ATG7,ATP1B3,ATP6AP2,B2M,Bst2,Casp12,CAV1,CNOT7,	59
Cell Death and Survival,Organismal Injury and Abnormalities	Cell death of epithelial cells	3.07E-07	-0.081	AAK1,APAF1,ARID1A,ATG7,BID,Casp12,CD44,CRYAB,CTH,CTNNB1,C	60
Cancer,Hematological Disease,Immunological Disease,Organismal Injury and	Mature T-cell neoplasm	3.5E-07		ADA,ANXA1,ARID1A,B2M,BCL2L13,C3,CAMLG,CCN1,CD47,COL1A1,C	59
Molecular Transport,Protein Trafficking	Transport of protein	3.58E-07	0.053	AP3B1,APPL1,ARL3,BID,C17orf75,CAV1,COL1A1,CTSA,EIF2D,FAM91A	41
Cancer,Neurological Disease,Organismal Injury and Abnormalities	Brain glioma	3.6E-07		A2M,AAK1,ABCF2,ABCF3,ABHD12,ACAD8,ACADM,ACADS,ACAT1,ACO	469
Neurological Disease,Skeletal and Muscular Disorders	Neuromuscular disease	3.69E-07	-1.233	A2M,ACADM,ACAT1,AEBP1,AFG3L2,ALB,ANXA1,ARL3,ATP5PO,ATP6A	93
Cancer,Organismal Injury and Abnormalities	Benign connective or soft tissue neopla	4.02E-07		ADGRE5,ANXA1,CAPN6,CCN1,COL18A1,COL1A1,COL1A2,COL2A1,CC	46
Cancer,Cell Death and Survival,Organismal Injury and Abnormalities,Tumor M	Cell death of cancer cells	4.02E-07	-0.045	AKT1S1,ALB,ANXA1,APAF1,AQR,ATG7,B2M,BID,CAPN6,CASP6,CCN1,	46
Cell Morphology,Nervous System Development and Function,Tissue Morpholo	Morphology of neurons	4.3E-07		A2M,AGA,APAF1,ARSA,ATG7,B2M,BLOC1S2,C3,Casp12,CAV1,CD47,C	67
Cancer,Hematological Disease,Organismal Injury and Abnormalities	Mature T-cell or NK-cell neoplasm	4.95E-07		ADA,ANXA1,ARID1A,B2M,BCL2L13,C3,CAMLG,CCN1,CD47,COL1A1,C	60
Neurological Disease,Organismal Injury and Abnormalities,Psychological Diso	Dementia	4.98E-07		A2M,ADGRL2,ALB,APOA1,APOB,ARSA,ATP6V1E1,C3,CAPN1,CASP6,C	69
Cellular Movement	Cell movement of colorectal cancer cell	5.2E-07	-1.3	CAPN1,CAV1,CD44,CD47,CSK,CTNNB1,DOCK5,EGFR,EIF4EBP1,ENPF	26
Cancer,Cell Death and Survival,Organismal Injury and Abnormalities,Tumor M	Cell viability of cancer cells	5.3E-07	-0.48	ATG7,CCN1,CD44,CTNNB1,EGFR,EXOC2,FADD,HMOX1,JMJD6,MTA1,	19
Digestive System Development and Function,Gastrointestinal Disease	Abnormal morphology of digestive syste	5.42E-07		ADA,AEBP1,AIDA,APAF1,ARAF,B2M,BID,BPNT1,C3,CAV1,COL3A1,CTH	59
Cellular Movement	Cell movement of blood cells	5.7E-07	-0.66	Abcb1b,ADA,ADGRE5,AKR1B1,ALB,ANO6,ANXA1,APOA1,APOB,ATG7,	100
Cell Cycle	Mitosis	5.92E-07	-1.521	ABRAXAS2,ADGRL2,ANLN,APAF1,ARSB,CBX5,CCN1,CDK13,CKAP2,C	57
Metabolic Disease,Organismal Injury and Abnormalities	Amyloidosis	6.36E-07	-0.414	A2M,ADGRL2,ALB,APOA1,ATP6V1E1,B2M,C3,CAPN1,CASP6,CAV1,CC	67
Cell Death and Survival,Organismal Injury and Abnormalities,Renal and Urolo	Necrosis of kidney	6.46E-07	-0.834	AAK1,ALB,ATG7,BID,C3,Casp12,CCN2,CD44,CRYAB,CTNNB1,EGFR,E	45
Cancer,Neurological Disease,Organismal Injury and Abnormalities	Grade 4 high grade glioma	6.54E-07		A2M,AAK1,ABCF2,ABCF3,ACAT1,ACOT2,ACP2,ACSF2,ACY3,ADA,ADG	363

Cancer,Neurological Disease,Organismal Injury and Abnormalities	Grade 4 malignant glioma	6.54E-07		A2M,AAK1,ABCF2,ABCF3,ACAT1,ACOT2,ACP2,ACSF2,ACY3,ADA,ADG	363
Cancer,Neurological Disease,Organismal Injury and Abnormalities	Grade 4 astrocytoma	6.54E-07		A2M,AAK1,ABCF2,ABCF3,ACAT1,ACOT2,ACP2,ACSF2,ACY3,ADA,ADG	363
Neurological Disease,Organismal Injury and Abnormalities	Gliosis	6.6E-07	2.079	ABHD12,ACP2,AGA,APOA1,ARSA,BID,CAV1,CHSY1,COQ9,DNAJC5,FU	22
Gene Expression	Transcription of RNA	6.69E-07	-1.278	A2M,AEBP1,ARID1A,BID,BLOC1S2,CAND1,CASK,CAV1,CBX5,CCN1,C(	158
Lipid Metabolism,Small Molecule Biochemistry	Catabolism of glycosphingolipid	6.95E-07	-2.391	ARSA,GBA,GLA,GLB1,GM2A,HEXA,HEXB,SMPD4	8
Cancer,Hematological Disease,Immunological Disease,Organismal Injury and	Mature B-cell lymphoma	7.09E-07		ADA,ADAM9,ANXA1,ARID1A,B2M,BCAT1,CAMLG,CAV1,CD63,COMT,C	64
Developmental Disorder,Hereditary Disorder,Metabolic Disease,Organismal In	Inborn error of carbohydrate metabolism	7.32E-07		ARSB,ATP6AP2,C3,CCDC115,COG1,COG2,COG4,CTSA,DBNL,FUCA1,	22
Protein Synthesis	Quantity of interleukin	7.38E-07	0.741	C3,Casp12,CAV1,CD44,HDAC6,HEXB,IFI16,IFIT2,IL1RN,IL6ST,Irgm1,KR	21
Cancer,Neurological Disease,Organismal Injury and Abnormalities	Grade 1-4 astrocytoma	7.98E-07		A2M,AAK1,ABCF2,ABCF3,ACAT1,ACOT2,ACP2,ACSF2,ACY3,ADA,ADG	364
Cancer,Neurological Disease,Organismal Injury and Abnormalities	Brain astrocytoma	8.01E-07		A2M,AAK1,ABCF2,ABCF3,ACAT1,ACOT2,ACP2,ACSF2,ACY3,ADA,ADG	363
Nucleic Acid Metabolism,Small Molecule Biochemistry	Metabolism of nucleotide	8.42E-07	-0.445	Abcb1b,ACAT1,ADA,AK3,AMPD2,APOA1,ATP5PO,ATP6AP2,BPNT1,CA	51
Cellular Movement,Hematological System Development and Function,Immune	Cell movement of leukocytes	8.73E-07	-0.857	Abcb1b,ADA,ALB,ANO6,ANXA1,APOA1,ATG7,BID,C3,CAV1,CCN1,CCN	86
Cardiovascular Disease,Hereditary Disorder,Organismal Injury and Abnormali	Familial cardiovascular disease	8.78E-07	0.547	AGK,ALB,APOA1,APOB,C3,CAMLG,CAV1,CCN2,CCNH,CDK13,COG2,C	56
Organismal Development	Morphology of head	8.96E-07		ADAM9,AGA,ANXA1,APAF1,ARSA,ARSB,ATG7,B2M,BID,BLOC1S2,Brd4	97
Antimicrobial Response,Inflammatory Response	Antiviral response	8.96E-07	-2.604	ATG7,Bst2,CNOT7,DDX21,DDX58,EGFR,EIF2AK2,EXOSC4,EXOSC5,FA	29
Immunological Disease	Systemic autoimmune syndrome	9.06E-07	0.485	ACADM,ACAT1,ACO1,ACSL5,ADA,ADAM9,ADGRE5,AKR1A1,AKR1B1,A	124
Organismal Injury and Abnormalities,Tissue Morphology	Abnormal morphology of epithelial tissu	9.16E-07		ADA,ADAM9,AGA,AIDA,ARSB,BID,BPNT1,CAV1,CCN2,CD44,COL18A1,	47
Cell Death and Survival,Gastrointestinal Disease,Hepatic System Disease,Org	Necrosis of liver	9.33E-07	0.766	ADA,AGA,BID,C3,CTH,CTNNB1,DNM1L,EGFR,FADD,HADHA,HK2,HMO	32
Gene Expression	Transcription	9.36E-07	-0.893	A2M,ACO1,ADNP,AEBP1,APOB,ARID1A,BID,BLOC1S2,C3,CAND1,CAS	182
Cancer,Organismal Injury and Abnormalities	Subcutaneous tumor	9.75E-07		ADGRE5,ANXA1,CAMLG,CAPN6,CCN1,COL18A1,COL1A1,COL1A2,CO	46
Organismal Injury and Abnormalities,Reproductive System Disease	Benign uterine disease	9.98E-07		ADGRE5,ANXA1,CAPN6,CCN1,COL18A1,COL1A1,COL1A2,COL2A1,CO	35
Infectious Diseases	Replication of virus	1.02E-06	1.347	ARAF,ATG4B,ATG7,ATP1B3,ATP6AP2,B2M,Bst2,Casp12,CAV1,CNOT7,	62
Cancer,Hematological Disease,Immunological Disease,Organismal Injury and	Tumorigenesis of lymphocytes	1.03E-06	0.484	ABRAXAS2,ACP6,ADA,ADAM9,ADD3,ADGRL2,AKT1S1,ALB,ALDH9A1,A	193
Organismal Injury and Abnormalities	Organ Degeneration	1.03E-06	2.242	ABHD12,ACADM,ADAM9,AFG3L2,AGA,ARL3,ARSA,ATP6AP2,C3,CAPN	63
Cell-To-Cell Signaling and Interaction,Inflammatory Response	Immune response of tumor cell lines	1.04E-06	-0.224	ANXA1,ARAF,ATG7,CD44,CD47,CKB,CSK,CTNNB1,DNM1L,EIF4EBP1,E	29
Cancer,Organismal Injury and Abnormalities	Adenoma	1.04E-06	0.054	ACSF2,AEBP1,APOA1,ARAF,ASCC3,ATG7,BID,C3,CAV1,CD44,CDKN2I	56
Cancer,Gastrointestinal Disease,Organismal Injury and Abnormalities	Colorectal tumor	1.06E-06	1.787	A2M,ABCF2,ABCF3,ACADM,ACADS,ACAP2,ACAT1,ACBD3,ACP6,ACSL	412
Cell Death and Survival,Organismal Injury and Abnormalities,Renal and Urolo	Cell death of kidney cells	1.09E-06	-0.849	AAK1,ALB,ATG7,BID,C3,Casp12,CCN2,CD44,CRYAB,CTNNB1,EGFR,EI	43
Nucleic Acid Metabolism	Metabolism of nucleic acid component o	1.17E-06	-0.843	Abcb1b,ACAT1,ACOT2,ACSF2,ADA,AK3,AMPD2,APOA1,ATP5PO,ATP6	58
Nervous System Development and Function	Morphology of nervous system	1.21E-06		A2M,ACP2,ADAM9,AGA,ANXA1,APAF1,ARSA,ATG7,B2M,BID,BLOC1S2	94
Cellular Movement,Immune Cell Trafficking	Leukocyte migration	1.22E-06	-0.689	Abcb1b,ADA,ADGRE5,ALB,ANO6,ANXA1,APOA1,APOB,ATG7,ATP1B3,E	98
Cancer,Organismal Injury and Abnormalities	Genitourinary adenocarcinoma	1.24E-06	1.285	A2M,AAK1,ABCF2,ABCF3,ACAD8,ACADM,ACADS,ACAP2,ACAT1,ACP6	485
Inflammatory Response,Organismal Injury and Abnormalities	Inflammation of organ	1.37E-06	3.499	ADA,ALB,ANXA1,APOA1,APOB,ARFGAP3,ARID1A,ARSA,ASNS,ATG7,E	132
Connective Tissue Development and Function,Skeletal and Muscular System	Morphology of bone	1.42E-06		ACP2,APAF1,ARSB,ATG7,CAPN1,CAV1,CCN2,CD44,CD47,CETN2,CHK	59
Cellular Growth and Proliferation	Cytostasis	1.46E-06	-0.769	Abcb1b,ADA,CAV1,CCN1,COL18A1,COPZ1,COPZ2,CRABP2,CTH,CTNN	34
Cancer,Organismal Injury and Abnormalities,Reproductive System Disease	Benign neoplasm of female genital orga	1.46E-06		ADGRE5,ANXA1,CAPN6,CCN1,COL18A1,COL1A1,COL1A2,COL2A1,CO	34
Cellular Movement,Hematological System Development and Function,Immune	Cellular infiltration by macrophages	1.52E-06	1.125	ANXA1,APOA1,BID,C3,CAV1,CCN1,CCN2,CD44,CTNNB1,EGFR,GBA,H	28
Cardiovascular Disease,Hereditary Disorder,Organismal Injury and Abnormali	Familial vascular disease	1.54E-06		ALB,C3,CAV1,CCN2,CCNH,COL1A1,COL3A1,COLGALT1,DDX58,EGFR,	33
Lipid Metabolism,Small Molecule Biochemistry	Metabolism of glycolipid	1.54E-06	-2.345	Abcb1b,APOA1,ARSA,CD9,CTSA,GBA,GLA,GLB1,GM2A,GPAA1,HEXA,I	19
Organismal Development	Size of body	1.57E-06	-3.828	ADA,AEBP1,AFG3L2,ARAF,ARSB,ATG7,BCAT2,C3,CAV1,CETN2,CHTF	75
Protein Degradation,Protein Synthesis	Catabolism of protein	1.58E-06	-0.979	AAK1,ACY1,ADAM9,ADI1,AFG3L2,APAF1,APOA1,APOB,ATG4B,ATG7,A	67
Cancer,Hematological Disease,Organismal Injury and Abnormalities	Mature lymphocytic neoplasm	0.0000016		ADA,ADAM9,ADGRL2,AKT1S1,ANXA1,ARAF,ARID1A,ASCC3,ATP6V1E1	152
Lipid Metabolism,Molecular Transport,Small Molecule Biochemistry	Concentration of lipid	1.62E-06	1.221	Abcb1b,ABHD12,ACAT1,ACOT2,ACP6,ADA,AEBP1,AIDA,AKR1B1,ALB,A	85
Cell Morphology,Tissue Morphology	Morphology of epithelial cells	1.64E-06		ADA,AIDA,ARSA,BID,BPNT1,Brd4,CAV1,COL1A1,CTHRC1,CTNNB1,EG	33
Cell Death and Survival,Embryonic Development	Cell death of embryonic cell lines	1.65E-06	-1.426	AAK1,APAF1,ATG7,BID,CD44,CRYAB,CTNNB1,DAPK3,DNM1L,EGFR,E	44
Metabolic Disease,Organismal Injury and Abnormalities	Disorder of lipid metabolism	1.65E-06	0.984	ACAD8,ACADM,ACADS,ACAT1,APOA1,APOB,ARSA,ARSB,CAMLG,CAV	35
Neurological Disease,Organismal Injury and Abnormalities	Progressive encephalopathy	1.72E-06		A2M,ADGRL2,ALB,ANXA1,APOA1,ARSA,ATP6V1E1,C3,CAPN1,CASP6,	94
Cellular Development,Cellular Growth and Proliferation,Connective Tissue De	Formation of osteoclasts	1.77E-06	-0.151	CAPN1,CAV1,CD44,CD47,CD9,CTNNB1,EGFR,EIF2AK2,FHL2,ICAM1,IF	25
Neurological Disease,Organismal Injury and Abnormalities,Psychological Diso	Degenerative dementia	1.78E-06		A2M,ADGRL2,ALB,APOA1,ARSA,ATP6V1E1,C3,CAPN1,CASP6,CAV1,C	63
Lipid Metabolism,Small Molecule Biochemistry	Catabolism of sphingolipid	1.83E-06	-2.391	ACSL5,ARSA,GBA,GLA,GLB1,GM2A,HEXA,HEXB,PPT1,SMPD4	10
Hereditary Disorder,Neurological Disease,Organismal Injury and Abnormalitie	Huntington Disease	1.96E-06		A2M,ACADM,ACAT1,AEBP1,ARL3,ATP5PO,ATP6AP2,B2M,C3,CASP6,C	59
Hematological System Development and Function,Lymphoid Tissue Structure	Morphology of lymphoid organ	2.01E-06		ACP2,ADA,AGK,ARAF,ATP6AP2,Brd4,Bst2,C3,CD47,CDKN2B,CKS1B,C	52
Developmental Disorder,Neurological Disease,Organismal Injury and Abnorm	Congenital anomaly of central nervous s	2.03E-06	0.014	ACADM,AMPD2,APAF1,APOB,ARID1A,CASK,COASY,COL18A1,COL2A1	37
Lipid Metabolism,Small Molecule Biochemistry	Metabolism of glycosphingolipid	2.06E-06	-2.066	Abcb1b,APOA1,ARSA,CD9,CTSA,GBA,GLA,GLB1,GM2A,HEXA,HEXB,H	15
Cancer,Hematological Disease,Immunological Disease,Organismal Injury and	Chronic myeloid leukemia	0.0000021	0.762	ADA,ANXA1,APAF1,BCAT1,CAMLG,CD44,CKS1B,CPT1A,CSK,CTNNB1	25
Cell Morphology,Cellular Function and Maintenance	Macroautophagy of cells	2.15E-06	-1.154	ATG4B,ATG7,GAA,HMOX1,IKBKG,MAP1LC3A,MTOR,PAFAH1B2,PGAM	19
Neurological Disease,Organismal Injury and Abnormalities	Cognitive impairment	2.16E-06	1.086	ACP6,ADNP,AGA,APOA1,APOB,ARHGEF10L,ARID1A,ARSA,ASCC3,AT	76
Nervous System Development and Function,Neurological Disease,Organ Mor	Abnormal morphology of telencephalon	2.18E-06		APAF1,ARSA,BLOC1S2,CAV1,CETN2,COL18A1,DCLK1,EGFR,GM2A,H	23
Cell Death and Survival	Cell death of fibroblasts	2.26E-06	-0.328	APAF1,ATG7,BID,CTBP2,CTNNB1,DDX58,EGFR,EIF2AK2,EPHX1,FADD	33
Inflammatory Response	Immune response of cells	2.35E-06	-3.262	Abcb1b,ANXA1,APOA1,ARAF,ATG7,Brd4,C3,CAV1,CD44,CD47,CKB,CO	66
Cancer,Organismal Injury and Abnormalities,Reproductive System Disease,Sk	Uterine leiomyoma	2.41E-06		ADGRE5,ANXA1,CAPN6,CCN1,COL18A1,COL1A1,COL1A2,COL2A1,CO	33
Infectious Diseases	Infection of cervical cancer cell lines	2.41E-06	-3.376	ADGRE5,ATG7,ATP5IF1,ATP6AP2,CAPN6,COG1,COG2,COG4,COPZ1,I	47
Cancer,Organismal Injury and Abnormalities,Skeletal and Muscular Disorders	Muscle tumor	2.46E-06		ADAM9,ADGRE5,ANXA1,CAPN6,CCN1,CD44,COL18A1,COL1A1,COL1A	71
Cancer,Hematological Disease,Immunological Disease,Organismal Injury and	Neoplasia of leukocytes	2.53E-06	0.486	ABRAXAS2,ACP6,ADA,ADAM9,ADD3,ADGRL2,AKT1S1,ALB,ALDH9A1,A	198
Cardiovascular System Development and Function,Tissue Development	Development of cardiovascular tissue	2.54E-06	-0.264	APOA1,ATP1B3,ATP5IF1,C3,CAV1,CCN1,CD276,CD44,CD9,COL18A1,C	45
Cellular Movement	Chemotaxis	2.72E-06	-2.086	A2M,ANO6,ANXA1,APOA1,C3,CAV1,CCN1,CCN2,CD44,CD47,CD9,COR	60

Cancer,Organismal Injury and Abnormalities,Reproductive System Disease,S	Uterine smooth muscle tumor	2.88E-06		ADGRE5,ANXA1,CAPN6,CCN1,COL18A1,COL1A1,COL1A2,COL2A1,CC	34
Cellular Movement	Cellular infiltration	2.89E-06	0.848	ADA,AKR1B1,ALB,ANXA1,APOA1,ATG7,ATP5IF1,BID,C3,CAV1,CCN1,C	55
Tissue Development	Growth of epithelial tissue	0.0000029	-0.43	APOA1,ATG7,ATP1B3,ATP5IF1,B2M,BID,C3,CASK,CAV1,CCN1,CD276,	74
Cancer,Gastrointestinal Disease,Organismal Injury and Abnormalities	Upper gastrointestinal tract tumor	2.91E-06	0.494	A2M,AAK1,ABCF2,ABCF3,ACAP2,ACAT1,ACO1,ACSF2,ADAM9,ADD3,A	349
Neurological Disease,Organismal Injury and Abnormalities,Psychological Diso	Alzheimer disease or frontotemporal de	0.000003		A2M,ADGRL2,ALB,APOA1,ARSA,ATP6V1E1,C3,CAPN1,CASP6,CAV1,C	62
Developmental Disorder	Autism spectrum disorder or intellectual	3.13E-06		ACP6,ACSL5,ADGRL2,ADNP,APOB,ARHGEF10L,ARID1A,ASCC3,ATG7	66
Tissue Morphology	Morphology of parenchyma	3.15E-06		ADA,BPNT1,Brd4,CAV1,CTHRC1,EGFR,GRN,HADHA,HEXA,HEXB,IKBK	15
Cancer,Organismal Injury and Abnormalities,Reproductive System Disease	Breast or ovarian carcinoma	3.17E-06	-0.126	AAK1,ABCF2,ABHD10,ACADS,ACP2,ADGRL2,ADNP,AEBP1,AGK,AK3,A	258
Organismal Development	Mass of organism	0.0000032	-2.106	APOA1,ATG7,Brd4,C3,CAV1,COL18A1,COMT,CUL7,EFL1,EGFR,ELOVL	43
Cellular Development,Cellular Growth and Proliferation	Colony formation of cells	0.0000033	0.304	ANXA1,ATG7,CAPN1,CAV1,CCN1,CD44,CD9,CDKN2B,CKS1B,CNOT7,C	60
Organismal Survival	Survival of organism	3.32E-06	-1.589	Abcb1b,ABRAXAS2,ADAM9,AEBP1,AKR1A1,ANXA1,APOA1,ATG7,B2M,	80
Dermatological Diseases and Conditions,Organismal Injury and Abnormalities	Ulceration of skin	3.35E-06		ANXA1,COL18A1,COL1A1,COL1A2,COL2A1,COL3A1,COL6A1,COL6A2,	14
Cancer,Gastrointestinal Disease,Organismal Injury and Abnormalities,Tumor M	Progression of digestive organ tumor	3.45E-06	-0.64	COL1A1,COL1A2,CRYAB,DNM1L,EGFR,PTGS2,PTK2,SMAD4,STAT3	9
Cellular Movement	Homing of cells	3.75E-06	-2.037	A2M,ANO6,ANXA1,APOA1,C3,CAV1,CCN1,CCN2,CD44,CD47,CD9,COR	62
Dermatological Diseases and Conditions,Organismal Injury and Abnormalities	Psoriasis	3.75E-06		A2M,ADGRE5,ANXA1,C3,CAMLG,CAV1,CD47,CD63,CNN1,COL1A2,CR	58
Neurological Disease	Progressive neurological disorder	3.77E-06	-1.423	A2M,ADGRL2,ALB,ANXA1,APOA1,ARSA,ATP6V1E1,C3,CAPN1,CASP6,	105
Hematological Disease,Immunological Disease	Lymphoproliferative disorder	3.84E-06	0.748	ABRAXAS2,ACP6,ADA,ADAM9,ADD3,ADGRL2,AKT1S1,ALB,ALDH9A1,A	202
Cancer,Hematological Disease,Organismal Injury and Abnormalities	Neoplasia of blood cells	3.96E-06	1.112	ABRAXAS2,ACP6,ADA,ADAM9,ADD3,ADGRL2,AKT1S1,ALB,ALDH9A1,A	264
Cell-To-Cell Signaling and Interaction	Response of tumor cell lines	3.98E-06	-0.426	ANXA1,ARAF,ATG7,CD44,CD47,CKB,CSK,CTNNB1,DNM1L,EIF4EBP1,E	32
Hematological System Development and Function,Lymphoid Tissue Structure	Morphology of lymphoid tissue	0.000004		ACP2,ADA,AGK,ARAF,ATP6AP2,Brd4,Bst2,C3,CD47,CDKN2B,CKS1B,C	55
Cell Death and Survival,Gastrointestinal Disease,Hepatic System Disease,Org	Cell death of liver cells	0.000004	0.519	BID,CTH,CTNNB1,DNM1L,EGFR,FADD,HK2,HMOX1,IKBKB,IKBKG,IL1R	27
Carbohydrate Metabolism,Lipid Metabolism,Molecular Transport,Small Molecu	Concentration of phosphatidic acid	4.01E-06	1.22	ACP6,ADA,APAF1,BID,CAV1,CDS2,CERS6,ELOVL1,HINT1,HTT,KNG1,L	23
Cellular Movement	Cell movement of melanoma cell lines	4.08E-06	-0.709	CAPN1,CCN1,CCN2,CD44,CNN1,COL18A1,EGFR,EPHA2,GBP2,ITGA2,	22
Carbohydrate Metabolism	Catabolism of carbohydrate	4.08E-06		ABHD12,AKR1A1,AP3B1,ARSB,CD44,CTH,GAA,GALE,GALK1,GLB1,GM	22
Cancer,Hematological Disease,Immunological Disease,Organismal Injury and	Lymphocytic cancer	4.17E-06	0.746	ABRAXAS2,ACP6,ADA,ADAM9,ADD3,ADGRL2,AKT1S1,ALB,ALDH9A1,A	201
Lipid Metabolism,Small Molecule Biochemistry	Fatty acid metabolism	4.19E-06	-1.114	Abcb1b,ACADS,ACAT1,ACBD3,ACOT2,ACSF2,ACSL5,ADA,AEBP1,AKR	63
Cancer,Cell Death and Survival,Organismal Injury and Abnormalities,Tumor M	Apoptosis of cancer cells	4.22E-06	-0.318	AKT1S1,ALB,ANXA1,ATG7,B2M,BID,CAPN6,CASP6,CD44,CTNNB1,EGF	28
Cancer,Organismal Injury and Abnormalities,Skeletal and Muscular Disorders	Leiomyoma	4.27E-06		ADGRE5,ANXA1,CAPN6,CCN1,COL18A1,COL1A1,COL1A2,COL2A1,CC	35
Cell Death and Survival	Cell death of carcinoma cell lines	4.28E-06	-0.065	ADI1,APAF1,ATG7,BID,CASP6,CAV1,CD44,CERS6,CLPTM1L,COL18A1	45
Skeletal and Muscular System Development and Function	Morphology of muscle	4.42E-06		ACADM,ADA,ARAF,CAPN6,CAV1,CCN2,CD47,COL6A1,CRYAB,CSRP2,	56
Protein Synthesis	Quantity of cytokine	4.46E-06	0.524	BCAT2,C3,Casp12,CAV1,CD44,CERS6,EIF4EBP1,HDAC6,HEXB,IFI16,IF	30
Neurological Disease,Organismal Injury and Abnormalities,Psychological Diso	Tauopathy	4.49E-06		A2M,ADGRL2,ALB,APOA1,ARSA,ATP6V1E1,C3,CAPN1,CASP6,CAV1,C	64
Cancer,Hematological Disease,Organismal Injury and Abnormalities	Lymphocytic neoplasm	4.54E-06	0.746	ABRAXAS2,ACP6,ADA,ADAM9,ADD3,ADGRL2,AKT1S1,ALB,ALDH9A1,A	201
Cardiovascular System Development and Function,Cell-To-Cell Signaling and	Interaction of endothelial cells	4.63E-06	-0.979	ADGRE5,C3,CCN1,CD44,CD47,CD63,COL18A1,CTNNB1,DNM1L,EGFR	27
Cellular Movement,Connective Tissue Development and Function	Cell movement of fibroblast cell lines	4.63E-06	-0.715	ADI1,APPL1,CD44,COL6A1,CORO1B,CRYAB,CSK,CTNNB1,EGFR,EPH	27
Lipid Metabolism,Molecular Transport,Small Molecule Biochemistry	Accumulation of ganglioside GM2	4.63E-06		GBA,GLB1,GM2A,HEXA,HEXB	5
Cell Death and Survival	Apoptosis of carcinoma cell lines	4.71E-06	0.238	ADI1,APAF1,ATG7,BID,CAV1,CD44,CERS6,CLPTM1L,COL18A1,CTNNB	40
Lipid Metabolism,Small Molecule Biochemistry	Storage of lipid	4.73E-06	1.251	APOA1,APOB,ARSA,C3,CAV1,EIF4EBP1,GLA,GM2A,HEXA,HEXB,LIPA,	15
Cancer,Organismal Injury and Abnormalities	Growth of tumor	4.99E-06	-1.848	ACAT1,ADAM9,AKR1B1,ANXA1,APOA1,ARID1A,ATG7,BCL2L13,CAV1,C	96
Lipid Metabolism,Small Molecule Biochemistry	Lipolysis	5.08E-06	-2.751	ABHD12,ACAT1,ADA,ANXA1,APOA1,ARSA,ATG7,C3,CAV1,CERS6,COL	30
Organismal Development,Organismal Injury and Abnormalities	Abnormal morphology of head	5.24E-06		ADAM9,AGA,APAF1,ARSA,ARSB,ATG7,B2M,BID,BLOC1S2,C3,CASP6,C	84
Connective Tissue Disorders,Metabolic Disease,Organismal Injury and Abnorm	Metabolic bone disease	0.0000054		AEBP1,ALB,COL1A1,COL1A2,COL3A1,ENPP1,GLB1,HNRNPC,NFKB2,F	15
Cellular Development,Cellular Function and Maintenance,Cellular Growth and	Assembly of cells	5.56E-06	-0.557	APOA1,ATP1B3,ATP5IF1,C3,CAV1,CCN1,CD276,CD44,CD9,COL18A1,C	44
Cellular Function and Maintenance	Endocytosis by eukaryotic cells	5.56E-06	-0.818	ANXA1,APOA1,ARAF,ATG7,ATP6V1E1,C3,CAV1,CD44,CD47,CKB,CSK,	44
Cancer,Cell Death and Survival,Organismal Injury and Abnormalities,Tumor M	Necrosis of tumor	5.68E-06	0.087	AKT1S1,ALB,ANXA1,APAF1,AQR,ATG7,B2M,BID,CAPN6,CASP6,CAV1,	50
Cancer,Hematological Disease,Organismal Injury and Abnormalities	Myeloid or lymphoid neoplasm	5.73E-06	1.349	ABRAXAS2,ACP6,ADA,ADAM9,ADD3,ADGRL2,AKT1S1,ALB,ALDH9A1,A	267
Connective Tissue Development and Function,Skeletal and Muscular System	Quantity of bone tissue	5.79E-06	-0.308	AKR1A1,ATG7,C3,CAPN1,CAV1,CD44,CD47,CHKB,COL1A1,COL2A1,C	24
Nervous System Development and Function,Neurological Disease,Organ Mor	Abnormal morphology of forebrain	5.83E-06		APAF1,ARSA,B2M,BLOC1S2,CAV1,CETN2,COL18A1,COL2A1,DCLK1,E	26
Cancer,Hematological Disease,Immunological Disease,Organismal Injury and	Plasma cell neoplasm	6.07E-06		ADAM9,AKT1S1,ARID1A,ATP6V1E1,B2M,BCAT1,CAMLG,CAV1,CD44,C	66
Cancer,Hematological Disease,Organismal Injury and Abnormalities	Hematologic cancer	6.13E-06	1.421	ABRAXAS2,ACP6,ADA,ADAM9,ADD3,ADGRL2,AKT1S1,ALB,ALDH9A1,A	266
Cancer,Cell Death and Survival,Organismal Injury and Abnormalities,Tumor M	Cell death of tumor cells	6.49E-06	0.087	AKT1S1,ALB,ANXA1,APAF1,AQR,ATG7,B2M,BID,CAPN6,CASP6,CAV1,	49
Cellular Development,Cellular Growth and Proliferation	Cell proliferation of carcinoma cell lines	6.83E-06	-0.711	ADAM9,AKT1S1,ANXA1,APAF1,ARAF,ARID1A,ASNS,ATG7,BID,CAV1,C	57
Energy Production,Lipid Metabolism,Small Molecule Biochemistry	Beta-oxidation of fatty acid	6.99E-06	0.192	ACADM,ACADS,ACSL5,ATG7,BID,CPT1A,CPT2,CTNNB1,DECR1,ECI2,H	15
Cancer,Organismal Injury and Abnormalities,Renal and Urological Disease	Urinary tract cancer	7.11E-06	0.577	ACADS,ACAT1,ACP6,AEBP1,AFG3L2,AK3,AKR1E2,ALB,ANLN,APAF1,A	188
Nutritional Disease	Obesity	7.16E-06	-1.643	ABCF2,AEBP1,AIDA,AK3,ALDH9A1,APOB,ATG7,ATP6AP2,BCAT2,C3,C	62
Cellular Movement	Migration of breast cancer cell lines	7.19E-06	-1.929	ADAM9,ANXA1,ARID1A,CAV1,CCN2,CD44,CD9,CERS6,CTNNB1,DDR2	38
Connective Tissue Disorders,Organismal Injury and Abnormalities,Skeletal an	Abnormal bone density	7.29E-06	1.166	AKR1A1,B2M,CD44,CD47,COG2,COL1A2,COL3A1,CTHRC1,CTNNB1,F	28
Gastrointestinal Disease,Organismal Injury and Abnormalities	Gastric lesion	0.0000073	0.396	A2M,AAK1,ABCF2,ABCF3,ACAP2,ACAT1,ACO1,ACSF2,ADAM9,ADD3,A	283
Gene Expression	Expression of mRNA	7.41E-06	0.066	ACO1,CD47,CDKN2B,COPZ1,DAPK3,EGFR,EIF2AK2,EIF2B2,EIF4E2,EI	31
Cell Morphology,Cellular Assembly and Organization	Morphology of lysosome	7.43E-06		ACP2,AGA,AP3B1,BLOC1S2,CTSA,FUCA1,GAA,HEXA,HEXB,MAN2B1	10
Metabolic Disease,Neurological Disease,Organismal Injury and Abnormalities	Central nervous system amyloidosis	7.45E-06		A2M,ADGRL2,ALB,APOA1,ATP6V1E1,C3,CAPN1,CASP6,CAV1,CCN2,C	60
Cellular Assembly and Organization,Cellular Function and Maintenance	Organization of cytoskeleton	7.81E-06	-2.991	A2M,ACAP2,ADI1,ADNP,AFG3L2,ANXA1,ARFGEF1,ARFIP2,ARL2,ARL3	129
Small Molecule Biochemistry	Catabolism of carboxylic acid	7.87E-06		ACADS,ACAT1,AKR1A1,ARSB,CD44,ECI2,GLB1,GNS,HEXA,HEXB,HTT	14
Cancer,Hematological Disease,Immunological Disease,Organismal Injury and	B-cell lymphoma	8.27E-06		ADA,ADAM9,ANXA1,ARID1A,ASCC3,B2M,BCAT1,CAMLG,CARMIL1,CA	94
Hematological System Development and Function,Immunological Disease,Infl	Enlargement of spleen	8.32E-06		AGK,Bst2,C3,CD47,CTSA,DDX58,FADS2,GBA,GPX7,HMOX1,IKBKG,IL6	28
Cell-To-Cell Signaling and Interaction,Cellular Function and Maintenance,Infla	Phagocytosis of tumor cell lines	8.33E-06	-0.11	ANXA1,ARAF,CD44,CD47,CKB,CSK,CTNNB1,ELOVL1,ERAP1,HMOX1,I	21

Developmental Disorder,Neurological Disease,Organismal Injury and Abnorm	Congenital malformation of brain	8.35E-06	0.014	ACADM,AMPD2,APAF1,APOB,ARID1A,CASK,COASY,COL18A1,COL2A	35
Cardiovascular System Development and Function,Cell-To-Cell Signaling and	Binding of endothelial cells	8.35E-06	-1.174	ADGRE5,C3,CCN1,CD44,CD47,CD63,COL18A1,CTNNB1,DNM1L,EGFR	26
Cell Death and Survival	Cellular degradation	8.72E-06	0.781	AFG3L2,AGA,ARSA,ATG7,CASP6,CAV1,CRYAB,DNM1L,FUCA1,GPX1,	32
Cancer,Gastrointestinal Disease,Organismal Injury and Abnormalities	Stomach tumor	8.88E-06	0.396	A2M,AAK1,ABCF2,ABCF3,ACAP2,ACAT1,ACO1,ACSF2,ADAM9,ADD3,A	282
Skeletal and Muscular System Development and Function	Quantity of bone	8.98E-06	-1.381	AKR1A1,ATG7,C3,CAPN1,CAV1,CD44,CD47,CHKB,CHSY1,COL1A1,CO	30
Cancer,Gastrointestinal Disease,Organismal Injury and Abnormalities	Non-colon gastrointestinal cancer	9.02E-06	0.055	A2M,AAK1,ABCF2,ABCF3,ACAP2,ACAT1,ACO1,ACSF2,ADAM9,ADD3,A	359
Cell Morphology,Nervous System Development and Function,Neurological Dis	Abnormal morphology of neurons	9.18E-06		AGA,APAF1,ARSA,ATG7,BLOC1S2,C3,Casp12,CAV1,CD47,CETN2,CKB	51
Carbohydrate Metabolism	Glycolysis of cells	9.19E-06	-0.725	ANXA1,ATP5IF1,ATP6AP2,BCAT1,BCL2L13,CAV1,CTH,DNAJB1,EGFR,	24
Neurological Disease,Organismal Injury and Abnormalities	Neurodegeneration of cerebellum	0.0000093	0.508	AFG3L2,AGA,ARSA,C3,DNM1L,FUCA1,HEXB,HTT,MARVELD1,PPT1,Sa	11
Hematological System Development and Function,Immunological Disease,Ly	Abnormal morphology of spleen	0.0000093		AGK,Bst2,C3,CD47,CDKN2B,CTSA,DDX58,FADS2,GBA,GPX7,HELLS,H	35
Cellular Movement	Cell movement of myeloid cells	9.47E-06	-1.393	ALB,ANO6,ANXA1,APOA1,BID,C3,CAV1,CCN1,CCN2,CD44,CD47,CD9,	62
Cell Morphology,Nervous System Development and Function,Tissue Morpholo	Morphology of neurites	9.55E-06		A2M,AGA,ARSA,ATG7,C3,CAV1,CD47,CKB,CLASP2,COQ9,CTNNB1,DE	30
Nucleic Acid Metabolism,Small Molecule Biochemistry	Synthesis of purine nucleotide	9.83E-06	-0.739	Abcb1b,ACAT1,AK3,AMPD2,APOA1,ATP5PO,CAV1,COASY,DNM1L,HM	25
Cellular Movement	Cellular infiltration by blood cells	9.85E-06	0.799	ADA,AKR1B1,ANXA1,APOA1,ATG7,BID,C3,CAV1,CCN1,CCN2,CD276,C	49
Neurological Disease	Dyskinesia	9.87E-06		A2M,ACADM,ACAT1,AEBP1,ARL3,ATP5PO,ATP6AP2,B2M,C3,CASP6,C	61
Cancer,Gastrointestinal Disease,Organismal Injury and Abnormalities	Tumorigenesis of gastrointestinal tumor	9.88E-06	1.91	A2M,ABCF2,ABCF3,ACADM,ACAP2,ACAT1,ACBD3,ACP6,ADA,ADD3,A	363
Cellular Movement,Connective Tissue Development and Function	Migration of fibroblast cell lines	9.89E-06	-1.296	ADI1,APPL1,COL6A1,CORO1B,CRYAB,CSK,CTNNB1,EGFR,FHL2,IFI16	21
Cardiovascular System Development and Function,Tissue Development	Development of endothelial tissue	9.97E-06	-0.356	APOA1,ATP1B3,ATP5IF1,C3,CAV1,CCN1,CD276,CD44,CD9,COL18A1,C	43
Cellular Compromise	Fragmentation of mitochondria	0.00001	-3.095	ATG7,BID,DNM1L,HTT,IKBKB,LCLAT1,MARCHF5,OPA1,PRKCD,SHC1,	12
Cancer,Organismal Injury and Abnormalities	Lymphatic system tumor	0.0000101	0.671	ABRAXAS2,ACP6,ADA,ADAM9,ADD3,ADGRL2,AKT1S1,ALB,ALDH9A1,	202
Cancer,Gastrointestinal Disease,Organismal Injury and Abnormalities	Upper gastrointestinal carcinoma	0.0000103	-0.692	A2M,AAK1,ABCF2,ABCF3,ACAP2,ACAT1,ACO1,ACSF2,ADAM9,ADD3,A	336
Cancer,Gastrointestinal Disease,Organismal Injury and Abnormalities	Upper gastrointestinal tract cancer	0.0000107	0.055	A2M,AAK1,ABCF2,ABCF3,ACAP2,ACAT1,ACO1,ACSF2,ADAM9,ADD3,A	344
Cancer,Gastrointestinal Disease,Organismal Injury and Abnormalities	Hepatobiliary carcinoma	0.0000108	-0.273	A2M,ABCF2,ABCF3,ACBD3,ACO1,ACY1,ADA,ADAM9,ADNP,AEBP1,AG	345
Cell Morphology,Cellular Assembly and Organization,Cellular Function and M	Depolarization of mitochondria	0.0000111	-1.206	APAF1,BID,CCN1,COL1A1,DNM1L,EIF2AK2,GCLC,HTT,PTEN,TSPO,VD	11
Lipid Metabolism,Molecular Transport,Small Molecule Biochemistry	Accumulation of sphingolipid	0.0000112	2.244	ARSA,CERS6,GBA,GLA,GLB1,GM2A,GULP1,HEXA,HEXB,PHGDH	10
Cancer,Cell Death and Survival,Organismal Injury and Abnormalities,Tumor M	Apoptosis of cancer stem cells	0.0000113	-0.254	ATG7,BID,EGFR,RBPJ	4
Neurological Disease,Organismal Injury and Abnormalities	Astrocytosis	0.0000115	1.623	ACP2,AGA,APOA1,ARSA,CAV1,COQ9,FUCA1,GRN,HTT,LRPAP1,MME,	15
Infectious Diseases	Replication of Flaviviridae	0.0000115	2.541	ATG4B,ATG7,Casp12,DDX58,EIF2AK2,HMOX1,IFITM3,ISG15,MAP1LC3	15
Cell Death and Survival	Cell death of central nervous system ce	0.0000115	0.079	A2M,ADNP,ALB,APAF1,ATG7,BID,CAPN1,Casp12,CASP6,CRYAB,DNM	35
Cellular Development,Cellular Growth and Proliferation,Connective Tissue De	Development of connective tissue cells	0.0000115	-0.328	CAPN1,CAV1,CD44,CD47,CD9,CHSY1,CTNNB1,EGFR,EIF2AK2,ENPP1	36
Infectious Diseases	Infection by Retroviridae	0.0000116	-2.154	ADA,ADGRE5,AFG3L2,ALB,APOA1,AQR,ARID1A,ATG7,B2M,Bst2,C3,C	69
Cell Death and Survival,Skeletal and Muscular Disorders	Necrosis of muscle	0.0000117	0.368	APAF1,APOA1,BID,CAPN1,CAV1,CCN1,CNBP,COL1A1,COL6A1,CRYA	39
Cellular Assembly and Organization,Cellular Function and Maintenance	Microtubule dynamics	0.0000117	-3.301	A2M,ACAP2,ADI1,ADNP,AFG3L2,ARFGEF1,ARFIP2,ARL2,ARL3,ARSA,	113
Organismal Injury and Abnormalities,Reproductive System Disease	Benign pelvic disease	0.0000119		ADGRE5,ANXA1,BCAT1,CAPN6,CCN1,CCN2,CD44,COL18A1,COL1A1,	59
Cardiovascular System Development and Function	Morphology of cardiovascular system	0.000012		ACADM,ADA,APOB,ARAF,ARSB,ATG7,ATP6V1E1,CAPN1,CAV1,CCN2,	86
Developmental Disorder,Hereditary Disorder,Metabolic Disease,Organismal In	Fatty acid oxidation disorder	0.0000122		ACAD8,ACADM,ACADS,CPT1A,CPT2,HADHA,HADHB,OXCT1,SLC25A2	9
Cellular Function and Maintenance	Engulfment of tumor cell lines	0.0000123	-0.098	ANXA1,ARAF,ATP6V1E1,CAV1,CD44,CD47,CKB,CSK,CTNNB1,DNM1,E	28
Neurological Disease,Organismal Injury and Abnormalities	Syndromic encephalopathy	0.0000123		ARID1A,ARL3,ATP6AP2,CASK,CASP6,CCN2,CDK13,CHAMP1,CHKB,C	58
Cancer,Organismal Injury and Abnormalities	Development of benign tumor	0.0000123	-0.269	ACSF2,AEBP1,APOA1,ARAF,ASCC3,ATG7,BID,C3,CAV1,CD44,CDKN2	63
Cellular Movement	Cell movement of breast cancer cell line	0.0000124	-1.835	ADAM9,ANXA1,ARID1A,CAV1,CCN2,CD44,CD9,CERS6,CTNNB1,DDR2	42
Immunological Disease,Inflammatory Disease	Lichen planus	0.0000129		B2M,CALML3,CD44,COL1A1,COL3A1,COX6A1,FADS2,GRN,HLA-A,IFIT	15
Cancer,Organismal Injury and Abnormalities,Reproductive System Disease	Breast carcinoma	0.000013	-0.218	AAK1,ABHD10,ACP2,ADGRL2,ADNP,AEBP1,ANLN,ANXA1,AP3B1,APA	182
Cancer,Organismal Injury and Abnormalities,Reproductive System Disease	Genital tumor	0.000013	1.886	A2M,AAK1,ABCF2,ABCF3,ACAD8,ACADM,ACADS,ACAP2,ACO1,ACP6,	497
Tissue Development	Development of epithelial tissue	0.0000132	-0.542	APOA1,ATP1B3,ATP5IF1,C3,CAV1,CCN1,CCN2,CD276,CD44,CD9,COL	57
Endocrine System Disorders,Gastrointestinal Disease,Metabolic Disease,Orga	Experimentally-induced diabetes	0.0000133		A2M,ANXA1,C3,EGFR,GBP2,HK2,HSPB1,ICAM1,IFITM3,IL1RN,LDLR,M	17
Skeletal and Muscular System Development and Function	Strength of muscle	0.0000133	-1.136	CAV1,DNAJC5,FUCA1,GAA,GAMT,HSPB1,HTT,IKBKB,LDLR,MGAT2,Sa	14
Cell Morphology,Digestive System Development and Function,Hepatic Systen	Morphology of hepatocytes	0.0000133		ADA,BPNT1,Brd4,CTHRC1,EGFR,GRN,HADHA,HEXA,HEXB,IKBKB,LIP	14
Cell Morphology	Morphology of cellular protrusions	0.0000134		A2M,AGA,ARSA,ATG7,C3,CALML3,CAV1,CD47,CKB,CLASP2,COQ9,CT	36
Carbohydrate Metabolism,Cellular Function and Maintenance	Glycolysis of tumor cell lines	0.0000137	-1.16	ATP5IF1,CAV1,DNAJB1,EGFR,HK2,IKBKB,MTOR,PDP1,PFKM,SLC16A	10
Cell Death and Survival,Cellular Compromise,Neurological Disease,Organism	Neurodegeneration of Purkinje cells	0.0000137	0.333	AFG3L2,AGA,ARSA,DNM1L,FUCA1,HEXB,HTT,MARVELD1,PPT1,Sacs	10
Metabolic Disease,Neurological Disease,Organismal Injury and Abnormalities	Alzheimer disease	0.0000141		A2M,ADGRL2,ALB,APOA1,ATP6V1E1,C3,CAPN1,CASP6,CAV1,CCN2,C	59
Cancer,Hematological Disease,Immunological Disease,Organismal Injury and	T-cell malignant neoplasm	0.0000142		ADA,ANXA1,ARHGEF10L,ARID1A,B2M,BCL2L13,C3,CAMLG,CCN1,CD4	71
Cardiovascular System Development and Function,Cellular Development,Cell	Endothelial cell development	0.0000142	-0.557	APOA1,ATP1B3,ATP5IF1,C3,CAV1,CCN1,CD276,CD44,CD9,COL18A1,C	42
Lipid Metabolism,Small Molecule Biochemistry	Metabolism of phospholipid	0.0000142	-1.715	ABHD12,ACP6,ACSL5,AGK,ANXA1,AP3B1,APOA1,CDS2,CHKB,CTBP2,	35
Infectious Diseases	HIV infection	0.0000143	-2.275	ADA,ADGRE5,AFG3L2,ALB,APOA1,AQR,ARID1A,ATG7,B2M,C3,CAPN6	68
Connective Tissue Development and Function,Skeletal and Muscular System	Quantity of bone cells	0.0000144	-0.308	AKR1A1,ATG7,C3,CAPN1,CAV1,CD44,CD47,CHKB,COL1A1,CTNNB1,E	23
Cellular Movement	Cell movement of connective tissue cell	0.0000146	-2.312	APPL1,ATP5IF1,CAPN1,CAV1,CCN1,CCN2,CD44,COL1A1,CTNNB1,DA	30
Post-Translational Modification	Aggregation of protein	0.0000146	1.029	ATG7,CRYAB,DNAJB1,HDAC6,HSPB1,HTT,SQSTM1,SUMO1	8
Gene Expression	Transactivation	0.0000147	-1.112	APPL1,ARID1A,CAV1,CCN2,CCNH,CD44,CIAO1,CTNNB1,DDX58,DNM1	56
Cancer,Organismal Injury and Abnormalities	Growth of solid tumor	0.0000149	0.318	AKR1B1,APOA1,ATG7,BCL2L13,CAV1,CCN1,CCN2,CD44,CD47,COL18	44
Cell Morphology,Digestive System Development and Function,Gastrointestina	Abnormal morphology of hepatocytes	0.000015		ADA,BPNT1,CTHRC1,EGFR,GRN,HADHA,HEXA,HEXB,IKBKB,LIPA,MA	13
Cell Death and Survival,Skeletal and Muscular Disorders	Cell death of muscle cells	0.0000152	0.187	APAF1,APOA1,BID,CAPN1,CAV1,CCN1,CNBP,COL1A1,CRYAB,CSK,CT	38
Cellular Movement,Hematological System Development and Function,Immune	Cellular infiltration by leukocytes	0.0000163	0.672	ADA,ANXA1,APOA1,ATG7,BID,C3,CAV1,CCN1,CCN2,CD276,CD44,CD4	48
Cancer,Hematological Disease,Immunological Disease,Organismal Injury and	Angioimmunoblastic T-cell lymphoma	0.0000163		ARID1A,C3,CCN1,COL6A1,COL6A2,CTNNB1,EPHA2,GTF2I,HDAC6,IDH	21
Cancer,Gastrointestinal Disease,Organismal Injury and Abnormalities	Colorectal cancer	0.0000163	2.925	A2M,ABCF2,ABCF3,ACADM,ACADS,ACAP2,ACAT1,ACBD3,ACP6,ACSL	398
Nervous System Development and Function,Neurological Disease	Abnormal morphology of nervous syster	0.0000166		ACP2,ADAM9,AGA,APAF1,ARSA,ATG7,B2M,BID,BLOC1S2,C3,Casp12,	74

Cancer,Organismal Injury and Abnormalities,Tissue Morphology	Quantity of carcinoma	0.0000166	-2.236	ARID1A,CCN1,COL18A1,EIF4EBP1,HINT1,IKBKG,IL6ST,MFGE8,PTEN,S	10
Hereditary Disorder,Neurological Disease,Organismal Injury and Abnormalitie	Hereditary polyneuropathy	0.0000167		ABHD12,AFG3L2,AMPD2,APOA1,CAPN1,COX6A1,DNM1L,ERLIN1,HAD	22
Cell-To-Cell Signaling and Interaction	Aggregation of cells	0.0000167	-3.347	AKR1B1,ALB,ARID1A,ATG7,C3,CAPN1,CD44,CD47,CD63,CD9,Cd99,CT	38
Cancer,Gastrointestinal Disease,Organismal Injury and Abnormalities	Gastric cancer	0.000017		A2M,AAK1,ABCF2,ABCF3,ACAP2,ACAT1,ACO1,ACSF2,ADAM9,ADD3,A	279
Skeletal and Muscular System Development and Function	Grip strength	0.0000172	-1.585	CAV1,DNAJC5,FUCA1,GAA,GAMT,HSPB1,HTT,LDLR,MGAT2,Sacs,SLC	13
Cellular Movement	Invasion of fibroblast cell lines	0.0000172	-0.777	ACAT1,CAV1,CD44,CRYAB,EGFR,FBLN1,ING4,MTOR,PTK2,RND3,STA	13
Cancer,Hematological Disease,Immunological Disease,Organismal Injury and	Plasma cell dyscrasia	0.0000172		ADAM9,AKT1S1,ARID1A,ATP6V1E1,B2M,BCAT1,CAMLG,CAV1,CD44,C	60
Cancer,Organismal Injury and Abnormalities	Development of adenocarcinoma	0.0000174	1.479	AAK1,ABCF2,ABCF3,ABRAXAS2,ACAD8,ACADM,ACADS,ACAP2,ACAT	427
Cell Death and Survival,Organismal Injury and Abnormalities	Cell death of epithelial cell lines	0.0000175	-0.837	AAK1,ATG7,BID,CD44,CRYAB,CTNNB1,CUL7,DECR1,EGFR,EIF2AK2,E	35
Connective Tissue Disorders,Inflammatory Disease,Organismal Injury and Ab	Rheumatic Disease	0.0000175	0.547	ACADM,ACAT1,ACO1,ACSL5,ADA,ADAM9,ADGRE5,AKR1A1,ALB,ANXA	114
Protein Synthesis	Quantity of protein in blood	0.0000176	0.285	ADA,AIDA,APOA1,APOB,BCAT2,BPNT1,C3,Casp12,CAV1,CD44,CERS6	50
Cellular Compromise,Cellular Function and Maintenance	Endoplasmic reticulum stress response	0.0000181	-1.131	BID,Casp12,COL3A1,CTH,DDRGK1,DERL1,DNAJB1,EIF2AK2,GAA,HMC	23
Cancer,Gastrointestinal Disease,Organismal Injury and Abnormalities	Colon tumor	0.0000182	0.81	A2M,ABCF2,ABCF3,ACADM,ACAP2,ACAT1,ACBD3,ACP6,ADA,ADD3,AI	359
Cancer,Hematological Disease,Immunological Disease,Organismal Injury and	B-cell neoplasm	0.0000183		ACP6,ADA,ADAM9,ADD3,ADGRL2,AKT1S1,ALB,ANXA1,ARAF,ARHGEF	160
Neurological Disease,Organismal Injury and Abnormalities	Leukoencephalopathy	0.0000188		ALB,ARSA,ARSB,CAV1,COX15,EIF2AK2,EIF2B2,GOLPH3,MT-ND1,NDU	19
Metabolic Disease,Organismal Injury and Abnormalities	Abnormal metabolism	0.0000192	0.984	ACAD8,ACADM,ACADS,ACAT1,AKR1A1,APOA1,APOB,ARSA,ARSB,CA	41
Lipid Metabolism,Small Molecule Biochemistry	Metabolism of sphingolipid	0.0000195	-1.865	Abcb1b,ACSL5,AGK,APOA1,ARSA,CD9,CERS6,CTSA,ELOVL1,GBA,GL	22
Cell Signaling,Vitamin and Mineral Metabolism	Handling of Ca2+	0.0000195	0.218	HTT,MCU,MICU1,MICU2,PKD2	5
Gene Expression	Transactivation of RNA	0.0000195	-1.188	APPL1,ARID1A,CAV1,CCNH,CD44,CIAO1,CTNNB1,DDX58,DNM1,EIF2A	53
Developmental Disorder,Hereditary Disorder,Metabolic Disease,Organismal In	Nuclear type 1 mitochondrial complex I	0.0000196		NDUFA10,NDUFA2,NDUFB3,NDUFS3,NDUFS7,NDUFS8,NDUFV2	7
Cell Death and Survival	Cell viability of neuroblastoma cell lines	0.0000196	1.66	ALB,HK2,HMOX1,HSPB1,HTT,Nes,P4HB,PFKM,PRKCD,PRPH,PTGS2,F	13
Connective Tissue Development and Function,Skeletal and Muscular System	Volume of bone	0.00002		ATG7,CAPN1,CAV1,CCN2,CD47,CHSY1,COL1A1,COL1A2,FBLIM1,GBA	20
Cellular Assembly and Organization	Development of cytoplasm	0.0000203	-1.296	ANKRD13A,ANXA1,APOA1,APPL1,ARFGEF1,ARHGEF10L,ARL2,ASB7,	54
Cell Death and Survival	Apoptosis of colorectal cancer cell lines	0.0000203	0.861	APAF1,ATG7,BID,CD44,CKAP2,COL18A1,CTBP2,CTNNB1,EGFR,EPHB	32
Cellular Development,Cellular Growth and Proliferation,Connective Tissue De	Colony formation of fibroblast cell lines	0.0000206	0.747	CD44,CDKN2B,CNOT7,CTNNB1,EGFR,FBLN1,GOLPH3,IFI16,MAPK3,M	16
Carbohydrate Metabolism	Glycolysis	0.0000212	-0.854	ANXA1,ATP5IF1,ATP6AP2,BCAT1,BCL2L13,CAV1,CTH,DNAJB1,DNM1L	25
Cancer,Hematological Disease,Immunological Disease,Organismal Injury and	B cell cancer	0.0000212		ACP6,ADA,ADAM9,ADD3,AKT1S1,ALB,ANXA1,ARHGEF10L,ARID1A,AS	148
Cancer,Gastrointestinal Disease,Hepatic System Disease,Organismal Injury a	Hepatobiliary system cancer	0.0000215	-0.082	A2M,ABCF2,ABCF3,ACBD3,ACO1,ACY1,ADA,ADAM9,ADNP,AEBP1,AG	352
Cancer,Organismal Injury and Abnormalities	Refractory malignant tumor	0.0000215		ADA,ANXA1,ARAF,CAMLG,CSK,DDR2,EGFR,EPHA2,HDAC6,HMOX1,ID	26
Cancer,Organismal Injury and Abnormalities	Pelvic adenocarcinoma	0.0000224		A2M,AAK1,ABCF2,ABCF3,ACAD8,ACADM,ACADS,ACAP2,ACP6,ACSF2	441
Cellular Development,Cellular Growth and Proliferation,Connective Tissue De	Development of fibroblast cell lines	0.0000225	0.235	CD44,CDKN2B,CNOT7,CTNNB1,DNM1L,EGFR,FBLN1,GOLPH3,GRN,IF	18
Cell-To-Cell Signaling and Interaction,Cellular Assembly and Organization	Cell-cell contact	0.0000227	-1.275	ADAM9,ADGRE5,ADGRL2,ADNP,AFG3L2,ANXA1,AP3B1,APOB,ARSA,C	76
Lipid Metabolism,Molecular Transport,Small Molecule Biochemistry	Accumulation of lipid	0.0000228	1.35	Abcb1b,ACAT1,ACP2,AP3B1,APOA1,APOB,ARSA,ATG7,ATP5IF1,BID,C	37
Nervous System Development and Function,Organ Morphology,Organismal D	Morphology of cerebral cortex	0.0000229		APAF1,ATG7,BID,BLOC1S2,C3,CASP6,CAV1,CTNNB1,EGFR,GM2A,HD	28
Gastrointestinal Disease,Hepatic System Disease,Metabolic Disease,Organis	Microvesicular hepatic steatosis	0.0000237	1.342	ACADM,ACADS,CPT1A,CPT2,CTHRC1,DECR1,HADHA,LDLR,PC	9
Inflammatory Response	Inflammation of absolute anatomical reg	0.0000237	2.707	ADA,ALB,ANXA1,APOA1,APOB,ARID1A,ARSA,ASNS,ATG7,B2M,BID,C1	112
Cancer,Gastrointestinal Disease,Organismal Injury and Abnormalities	Gastric carcinoma	0.0000239		A2M,AAK1,ABCF2,ABCF3,ACAP2,ACAT1,ACO1,ACSF2,ADAM9,ADD3,A	269
Nucleic Acid Metabolism,Small Molecule Biochemistry	Synthesis of nucleotide	0.0000248	-0.282	Abcb1b,ACAT1,ADA,AK3,AMPD2,APOA1,ATP5PO,ATP6AP2,CAV1,COA	39
Carbohydrate Metabolism	Uptake of carbohydrate	0.0000249	0.305	A2M,ALB,APOA1,APPL1,C3,CAV1,CD44,CPT1A,CTH,CTNNB1,EGFR,EX	35
Cancer,Organismal Injury and Abnormalities,Reproductive System Disease	Breast adenocarcinoma	0.0000249	0.927	AAK1,ANXA1,APAF1,ARFGEF1,ARID1A,ATP5IF1,C3,CAPN1,CASK,CCN	81
Cancer,Organismal Injury and Abnormalities,Reproductive System Disease	Genital tract cancer	0.0000257		A2M,AAK1,ABCF2,ABCF3,ACAD8,ACADM,ACADS,ACAP2,ACO1,ACP6,	489
Neurological Disease,Organismal Injury and Abnormalities	Abnormality of cerebrum	0.0000259	0.913	AGA,APAF1,ATG7,BID,BLOC1S2,C3,CAPN1,CASP6,CAV1,DNAJC5,EGF	30
Cell Death and Survival,Gastrointestinal Disease,Hepatic System Disease,Org	Apoptosis of liver cells	0.0000262	0.746	BID,CTNNB1,EGFR,FADD,IKBKB,IKBKG,IL6ST,JUND,LDLR,MAP2K4,MT	21
Cell Cycle,Cell-To-Cell Signaling and Interaction,Cellular Growth and Prolifera	Contact growth inhibition	0.0000262	-0.009	COPZ1,COPZ2,CTH,CTNNB1,DDX58,EGFR,GBP2,IFI16,IFIH1,ING4,Irgm	21
Cell Morphology,Cellular Assembly and Organization	Abnormal morphology of lysosome	0.0000267		ACP2,AGA,BLOC1S2,CTSA,FUCA1,GAA,MAN2B1	7
Carbohydrate Metabolism,Lipid Metabolism,Molecular Transport,Small Molecu	Concentration of phosphatidylserine	0.0000267	-1.342	APAF1,BID,CAV1,PHGDH,PML,PTEN,PTGS2	7
Tissue Development	Accumulation of cells	0.0000268	-0.071	ADGRE5,ANXA1,APOA1,B2M,BID,C3,CCN1,CD44,CKS1B,COL18A1,CR	38
Protein Synthesis	Synthesis of protein	0.000027	0.331	ACO1,AMPD2,ANXA1,BPNT1,C3,CAV1,CNBP,CNOT7,CTNNB1,DAPK3,	55
Cellular Growth and Proliferation,Tissue Development	Proliferation of epithelial cells	0.0000271	-0.244	ATG7,B2M,BID,C3,CASK,CAV1,CCN1,CD44,CD9,CDKN2B,COL8A1,CRY	54
Cellular Function and Maintenance,Inflammatory Response	Phagocytosis	0.0000272	-1.004	ANXA1,APOA1,ARAF,ATG7,C3,CAV1,CD44,CD47,CKB,COLEC12,CSK,C	43
Cell Death and Survival,Cellular Compromise,Neurological Disease,Tissue Mo	Degeneration of neurons	0.0000283	1.228	AFG3L2,AGA,ARSA,ATG7,CASP6,CAV1,DNM1L,FUCA1,GPX1,GRN,HE	29
Dermatological Diseases and Conditions,Organismal Injury and Abnormalities	Burn	0.0000285		ALB,ANXA1,COL18A1,COL1A1,COL1A2,COL2A1,COL3A1,COL6A1,COL	12
Carbohydrate Metabolism	Metabolism of monosaccharide	0.0000292		AKR1A1,AKR1B1,ATG7,CPT1A,FUCA1,GALE,GALK1,GLB1,HK2,HTT,M	17
Hematological System Development and Function,Tissue Morphology	Quantity of myeloid cells	0.0000295	-0.389	ABRAXAS2,ACAT1,ADA,ADGRE5,AGK,ANXA1,APOB,B2M,BID,BRCC3,	57
Carbohydrate Metabolism	Uptake of monosaccharide	0.0000295	0.068	A2M,ALB,APOA1,APPL1,C3,CAV1,CPT1A,CTH,CTNNB1,EGFR,EXOC7,	33
Cellular Movement	Invasion of breast cancer cell lines	0.0000297	-2.414	CAV1,CBX5,CCN1,CCNH,CD44,COL2A1,CTBP2,CTNNB1,DBNL,DNM1L	39
Hematological System Development and Function,Tissue Morphology	Quantity of blood cells	0.0000306	-2.902	ABRAXAS2,ACAT1,ADA,ADGRE5,AGK,ANXA1,AP3B1,APOA1,APOB,AF	97
Cancer,Gastrointestinal Disease,Organismal Injury and Abnormalities	Gastro-esophageal carcinoma	0.0000308		A2M,AAK1,ABCF2,ABCF3,ACAP2,ACAT1,ACO1,ACSF2,ADAM9,ADD3,A	285
Cellular Movement,Connective Tissue Development and Function	Cell movement of fibroblasts	0.0000314	-2.329	APPL1,CAV1,CCN1,CD44,COL1A1,CTNNB1,DAPK3,DDR2,GNA13,HSPE	25
Cellular Development,Cellular Growth and Proliferation,Connective Tissue De	Proliferation of fibroblast cell lines	0.0000317	-0.024	A2M,ACAT1,AKR1B1,Brd4,CAV1,CD9,CDKN2B,CKS1B,COL1A1,CTNNB	44
Cancer,Gastrointestinal Disease,Organismal Injury and Abnormalities	Development of intestinal tumor	0.0000318	1.929	A2M,ABCF2,ABCF3,ACADM,ACAP2,ACAT1,ACBD3,ACP6,ADA,ADD3,AI	358
Cellular Development,Cellular Growth and Proliferation	Development of tumor cell lines	0.0000319	0.841	ANXA1,ATG7,CAPN1,CAV1,CCN1,CD44,CDKN2B,CETN2,CKS1B,CNOT	46
Hereditary Disorder,Neurological Disease,Organismal Injury and Abnormalitie	Hereditary neuropathy	0.0000323		ABHD12,AFG3L2,AMPD2,APOA1,CAPN1,CAV1,CHCHD2,COA7,COASY	34
Cancer,Hematological Disease,Immunological Disease,Organismal Injury and	B-cell non-Hodgkin lymphoma	0.0000328		ADA,ADAM9,ANXA1,ARID1A,ASCC3,B2M,BCAT1,CAMLG,CARMIL1,CA	87
Antigen Presentation,Protein Synthesis	Quantity of MHC Class I on cell surface	0.000033	-1.969	B2M,ERAP1,STAT1,TAPBP	4
Cell Morphology,Organismal Injury and Abnormalities	Blebbing of nuclear envelope	0.000033		TOR1A,TOR1AIP1,TOR1AIP2,TOR3A	4

Lipid Metabolism,Small Molecule Biochemistry	Hydrolysis of glycosylceramide	0.000033	-1.931	ARSA,GBA,HEXA,HEXB	4
Free Radical Scavenging,Molecular Transport	Quantity of reactive oxygen species	0.0000336	-0.636	ADA,ADAM9,AKR1B1,ALB,APOB,ATG7,ATP5IF1,ATP6AP2,BID,CD44,DN	26
Neurological Disease,Organismal Injury and Abnormalities	Abnormality of cerebral cortex	0.0000336	0.913	APAF1,ATG7,BID,BLOC1S2,C3,CAPN1,CASP6,CAV1,DNAJC5,EGFR,GL	29
Cancer,Cell Death and Survival,Organismal Injury and Abnormalities,Tumor M	Apoptosis of tumor cells	0.0000346	-0.056	AKT1S1,ALB,ANXA1,ATG7,B2M,BID,CAPN6,CASP6,CAV1,CD44,COL18	32
Cancer,Hematological Disease,Immunological Disease,Organismal Injury and	Refractory myeloid leukemia	0.000035		CSK,DDR2,EPHA2,IDH1,IDH2,PDGFRB,PDP1,PML,POLD1,PRIM2,PSM	14
Cell Death and Survival,Gastrointestinal Disease,Hepatic System Disease,Org	Cell death of hepatocytes	0.0000356	0.449	BID,CTH,CTNNB1,DNM1L,EGFR,FADD,HMOX1,IKBKB,IKBKG,IL1RN,LD	21
Cell-To-Cell Signaling and Interaction,Reproductive System Development and	Binding of breast cell lines	0.0000358	-1.287	CD44,CTNNB1,ITGA2,KPNA2,NAGA,PDLIM2,SERPINH1	7
Cancer,Gastrointestinal Disease,Organismal Injury and Abnormalities	Gastroesophageal cancer	0.0000361		A2M,AAK1,ABCF2,ABCF3,ACAP2,ACAT1,ACO1,ACSF2,ADAM9,ADD3,A	293
Cancer,Hematological Disease,Organismal Injury and Abnormalities	Myeloproliferative neoplasm	0.0000364	0.64	ADA,ANXA1,APAF1,ARAF,ATG7,BCAT1,CAMLG,CD44,CKS1B,CPT1A,C	32
Nervous System Development and Function,Organ Morphology,Organismal D	Morphology of brain	0.0000373		AGA,ANXA1,APAF1,ARSA,ATG7,B2M,BID,BLOC1S2,C3,CASP6,CAV1,C	52
Cancer,Organismal Injury and Abnormalities	Growth of carcinoma	0.0000382	-0.973	AKR1B1,APOA1,CAV1,CCN2,CTNNB1,EGFR,GRN,HDAC6,Ly6a (include	20
Cancer,Organismal Injury and Abnormalities	Glandular intraepithelial neoplasm	0.0000384		ANO6,APOB,ASCC3,C3,COL18A1,CTNNB1,EXOC3,FHL2,GBP2,GBP4,H	31
Cancer,Organismal Injury and Abnormalities,Reproductive System Disease	Ductal breast carcinoma	0.0000394		AAK1,ANXA1,APAF1,ARFGEF1,ARID1A,ATP5IF1,C3,CAPN1,CASK,CCN	76
Cancer,Gastrointestinal Disease,Organismal Injury and Abnormalities	Development of colorectal tumor	0.0000412	1.929	A2M,ABCF2,ABCF3,ACADM,ACAP2,ACAT1,ACBD3,ACP6,ADA,ADD3,A	357
Cancer,Hematological Disease,Organismal Injury and Abnormalities	Myelodysplastic/myeloproliferative neop	0.0000418		ANXA1,APAF1,CAMLG,CSK,DDR2,EPHA2,H2AX,IDH1,IDH2,MYH10,NC	18
Neurological Disease	Polyneuropathy	0.0000422		ABHD12,AFG3L2,AMPD2,APOA1,C3,CAPN1,COX6A1,DNM1L,ERLIN1,H	23
Cell Death and Survival	Apoptosis of lung cancer cell lines	0.0000427	0.999	APAF1,ATG7,ATP1B3,BID,CAV1,CERS6,CLPTM1L,DAPK3,DNM1,EGFR	31
Metabolic Disease,Organismal Injury and Abnormalities	Glucose metabolism disorder	0.0000428	1.615	A2M,ACO1,AEBP1,AIDA,AKR1B1,AKR1E2,ALB,ANXA1,APOA1,APOB,A	133
Cell Death and Survival	Cell death of immune cells	0.000043	-1.414	Abcb1b,ACAT1,ADA,AGA,ANXA1,APAF1,APOB,BID,C3,CAMLG,CASP6,	63
Energy Production,Lipid Metabolism,Small Molecule Biochemistry	Oxidation of fatty acid	0.000043	-0.844	ACADM,ACADS,ACSL5,APOA1,APPL1,ATG7,ATP5IF1,BID,C3,CPT1A,C	24
Cell Morphology,Organismal Injury and Abnormalities,Tissue Morphology	Abnormal morphology of epithelial cells	0.0000432		ADA,AIDA,BID,BPNT1,CAV1,CTHRC1,CTNNB1,EGFR,ELOVL1,FADD,F	25
Hereditary Disorder,Neurological Disease,Organismal Injury and Abnormalitie	Autosomal dominant encephalopathy	0.0000447		ADNP,ARID1A,CCN2,CDK13,CHAMP1,CHCHD2,COL1A1,CTNNB1,DNM	32
Cancer,Hematological Disease,Immunological Disease,Organismal Injury and	Refractory leukemia	0.0000463		ADA,ARAF,CAMLG,CSK,DDR2,EPHA2,IDH1,IDH2,PDGFRB,PDP1,PML,	17
Embryonic Development,Organismal Development,Tissue Morphology	Size of embryo	0.0000464		APOB,C3,COL2A1,CSK,CTNNB1,CUL7,DNM1L,EGFR,ERCC6L,FADD,H	33
Inflammatory Response	Inflammatory response	0.0000464	-0.381	ABHD12,ADA,ADGRE5,ANO6,ANXA1,AP3B1,APOA1,ATG7,BID,Brd4,C3	78
Skeletal and Muscular System Development and Function,Tissue Morphology	Quantity of trabecula	0.000047	-1.066	CAV1,CHSY1,COL1A2,CTHRC1,FBLIM1,IL6ST,Ly6a (includes others),PT	11
Cell-To-Cell Signaling and Interaction,Cellular Assembly and Organization	Turnover of focal adhesions	0.0000474	-1.89	APPL1,CAV1,CSK,DNM1,DOCK5,PSMD10,PTK2	7
Infectious Diseases	Replication of Hepatitis C virus	0.0000474	2.103	ATG4B,ATG7,DDX58,EIF2AK2,HMOX1,IFITM3,ISG15,MAP1LC3A,MAVS	13
Neurological Disease	Gait disturbance	0.0000474		AGA,ARSA,CAV1,COQ9,EFL1,GAA,GLB1,HELLS,HEXA,HEXB,HTT,MA	19
Cancer,Hematological Disease,Organismal Injury and Abnormalities	Chronic myeloproliferative neoplasm	0.0000474	0.277	ADA,ANXA1,APAF1,BCAT1,CAMLG,CD44,CKS1B,CPT1A,CSK,CTNNB1	27
DNA Replication, Recombination, and Repair	Metabolism of DNA	0.0000475	-1.606	APAF1,CASP6,CAV1,CD47,CRYAB,EGFR,EIF2AK2,ERCC6L,FADD,GP	41
Cancer,Hematological Disease,Immunological Disease,Organismal Injury and	Mature B cell malignant tumor	0.000048		ADA,ADAM9,AKT1S1,ANXA1,ARID1A,ASCC3,ATP6V1E1,B2M,BCAT1,C	106
Cancer,Gastrointestinal Disease,Organismal Injury and Abnormalities	Colorectal carcinoma	0.0000485		A2M,ABCF2,ABCF3,ACADM,ACAP2,ACAT1,ACBD3,ACP6,ADA,ADD3,A	356
Cardiovascular System Development and Function	Vascularization	0.0000487	-0.768	ADAM9,C3,CAV1,CCN1,CCN2,CD44,CD47,COL18A1,CRYAB,DDR2,EP	28
Cell Cycle	Senescence of cells	0.0000488	-0.742	ADD3,AKR1B1,ATG7,CAV1,CCN1,CDKN2B,CKS1B,CTNNB1,CUL7,EGF	35
Digestive System Development and Function,Hepatic System Development a	Growth of liver	0.0000489	-0.338	BID,C3,CAV1,CCN1,CCN2,COL1A1,CTNNB1,EGFR,FADD,IKBKB,IL1RN	24
Developmental Disorder	Hypoplasia of organ	0.0000519	0.457	AMPD2,CASK,CCN2,CNOT7,COASY,COL2A1,CTHRC1,DCLK1,DNM1L,	45
Inflammatory Response	Immune response of brain	0.0000521	1.259	B2M,C3,CAV1,CD276,CD44,CD47,CERS6,GRN,HLA-A,HSD17B7,IFI35,I	43
Reproductive System Development and Function	Morphology of reproductive system	0.0000532		ADA,AIDA,ALB,APAF1,APOA1,ARSA,ATG7,BCAT2,Brd4,C3,CAV1,CD44	63
Cell Cycle,Gene Expression	Binding of DNA	0.0000532	0.332	ALB,ARSB,BCAT2,Brd4,CAV1,CCN2,CTNNB1,DDX58,DNAJB1,EGFR,F	51

Table S3. Ingenuity disease and function analysis of mapped differentially expressed proteins between KO4 and Ctrl4 cells

Categories	Diseases or Functions Annotation	p-value	Activation z-s	Molecules	# Molecules
Cancer,Organismal Injury and Abnormalities	Nonhematologic malignant neoplasm	1.88E-23	1.028	A2M,AAK1,ACAD8,ACADM,ACAT2,ACOT2,ACP2,ACP6,ACSL1,ACY3,ADA,ADAM10,AD	541
Cancer,Organismal Injury and Abnormalities	Non-hematological solid tumor	1.92E-23	1.063	A2M,AAK1,ACAD8,ACADM,ACAT2,ACOT2,ACP2,ACP6,ACSL1,ACY3,ADA,ADAM10,AD	542
Cancer,Organismal Injury and Abnormalities	Cancer	8.44E-23	1.105	A2M,AAK1,ACAD8,ACADM,ACAT2,ACOT2,ACP2,ACP6,ACSL1,ACY3,ADA,ADAM10,AD	547
Cancer,Organismal Injury and Abnormalities	Non-melanoma solid tumor	1.21E-22	1.017	A2M,AAK1,ACAD8,ACADM,ACAT2,ACOT2,ACP2,ACP6,ACSL1,ACY3,ADA,ADAM10,AD	539
Cancer,Organismal Injury and Abnormalities	Malignant solid tumor	1.32E-21	1.439	A2M,AAK1,ACAD8,ACADM,ACAT2,ACOT2,ACP2,ACP6,ACSL1,ACY3,ADA,ADAM10,AD	544
Cancer,Organismal Injury and Abnormalities	Solid tumor	3.15E-21	1.775	A2M,AAK1,ACAD8,ACADM,ACAT2,ACOT2,ACP2,ACP6,ACSL1,ACY3,ADA,ADAM10,AD	545
Cancer,Organismal Injury and Abnormalities	Carcinoma	4.89E-21	0.604	A2M,AAK1,ACAD8,ACADM,ACAT2,ACOT2,ACP2,ACP6,ACSL1,ACY3,ADA,ADAM10,AD	532
Cancer,Organismal Injury and Abnormalities	Extracranial solid tumor	3.02E-20	1.648	A2M,AAK1,ACAD8,ACADM,ACAT2,ACOT2,ACP2,ACP6,ACSL1,ACY3,ADA,ADAM10,AD	541
Cancer,Organismal Injury and Abnormalities	Abdominal carcinoma	7.4E-15	0.786	A2M,AAK1,ACAD8,ACADM,ACAT2,ACP2,ACP6,ACSL1,ACY3,ADA,ADAM10,ADD3,AGK	491
Cancer,Organismal Injury and Abnormalities	Intraabdominal organ tumor	7.82E-15	1.623	A2M,AAK1,ACAD8,ACADM,ACAT2,ACP2,ACP6,ACSL1,ADA,ADAM10,ADD3,AGK,AHR,A	499
Cancer,Organismal Injury and Abnormalities	Adenocarcinoma	1.15E-14	1.511	A2M,AAK1,ACAD8,ACADM,ACAT2,ACP2,ACP6,ACSL1,ACY3,ADA,ADAM10,ADD3,AGK	478
Cancer,Organismal Injury and Abnormalities	Head and neck cancer	1.61E-14	1.446	A2M,AAK1,ACAD8,ACADM,ACAT2,ACOT2,ACP2,ACP6,ACSL1,ACY3,ADA,ADAM10,AD	456
Cancer,Organismal Injury and Abnormalities	Head and neck tumor	2.23E-14	1.062	A2M,AAK1,ACAD8,ACADM,ACAT2,ACOT2,ACP2,ACP6,ACSL1,ACY3,ADA,ADAM10,AD	478
Cancer,Organismal Injury and Abnormalities	Neck neoplasm	3.1E-14		A2M,AAK1,ACAD8,ACADM,ACAT2,ACOT2,ACP2,ACP6,ACSL1,ACY3,ADA,ADAM10,AD	430
Cancer,Organismal Injury and Abnormalities	Abdominal neoplasm	3.93E-14	1.302	A2M,AAK1,ACAD8,ACADM,ACAT2,ACP2,ACP6,ACSL1,ACY3,ADA,ADAM10,ADD3,AGK	503
Cancer,Endocrine System Disorders,Organismal Injury and Abnormalities	Thyroid cancer	5.57E-14		A2M,AAK1,ACAD8,ACADM,ACAT2,ACOT2,ACP2,ACP6,ACSL1,ACY3,ADA,ADAM10,AD	425
Cancer,Endocrine System Disorders,Organismal Injury and Abnormalities	Thyroid gland tumor	5.96E-14		A2M,AAK1,ACAD8,ACADM,ACAT2,ACOT2,ACP2,ACP6,ACSL1,ACY3,ADA,ADAM10,AD	426
Cancer,Organismal Injury and Abnormalities	Head and neck carcinoma	6.74E-14	1.446	A2M,AAK1,ACAD8,ACADM,ACAT2,ACOT2,ACP2,ACP6,ACSL1,ACY3,ADA,ADAM10,AD	442
Cancer,Endocrine System Disorders,Organismal Injury and Abnormalities	Thyroid carcinoma	8.07E-14		A2M,AAK1,ACAD8,ACADM,ACAT2,ACOT2,ACP2,ACP6,ACSL1,ACY3,ADA,ADAM10,AD	424
Cancer,Organismal Injury and Abnormalities	Abdominal cancer	1.12E-13	1.515	A2M,AAK1,ACAD8,ACADM,ACAT2,ACP2,ACP6,ACSL1,ACY3,ADA,ADAM10,ADD3,AGK	498
Cancer,Endocrine System Disorders,Organismal Injury and Abnormalities	Nonpituitary endocrine tumor	1.43E-13		A2M,AAK1,ACAD8,ACADM,ACAT2,ACOT2,ACP2,ACP6,ACSL1,ACY3,ADA,ADAM10,AD	428
Cancer,Organismal Injury and Abnormalities	Abdominal adenocarcinoma	6.23E-13	1.342	A2M,AAK1,ACAD8,ACADM,ACAT2,ACP2,ACP6,ACSL1,ACY3,ADA,ADAM10,ADD3,AGK	466
Cancer,Organismal Injury and Abnormalities	Cancer of secretory structure	1.34E-12		A2M,AAK1,ACAD8,ACADM,ACAT2,ACOT2,ACP2,ACP6,ACSL1,ACY3,ADA,ADAM10,AD	453
Cancer,Endocrine System Disorders,Organismal Injury and Abnormalities	Endocrine gland tumor	1.72E-12	0.816	A2M,AAK1,ACAD8,ACADM,ACAT2,ACOT2,ACP2,ACP6,ACSL1,ACY3,ADA,ADAM10,AD	437
Cancer,Endocrine System Disorders,Organismal Injury and Abnormalities	Endocrine cancer	1.74E-12		A2M,AAK1,ACAD8,ACADM,ACAT2,ACOT2,ACP2,ACP6,ACSL1,ACY3,ADA,ADAM10,AD	433
Cancer,Endocrine System Disorders,Organismal Injury and Abnormalities	Endocrine carcinoma	2.31E-12		A2M,AAK1,ACAD8,ACADM,ACAT2,ACOT2,ACP2,ACP6,ACSL1,ACY3,ADA,ADAM10,AD	432
Cancer,Gastrointestinal Disease,Organismal Injury and Abnormalities	Digestive system cancer	5.96E-12	2.081	A2M,AAK1,ACAD8,ACADM,ACAT2,ACP2,ACP6,ACSL1,ADA,ADAM10,ADD3,AGK,AHR,A	472
Infectious Diseases	Viral Infection	1.48E-11	-0.898	ACSL1,ADA,ADAM10,AHR,AKT2,ALB,ANXA2,ANXA6,APOA1,ARAF,ARFIP2,ARID1A,AR	127
Developmental Disorder,Hereditary Disorder,Organismal Injury and Abnormali	Autosomal dominant Emery-Dreifuss musc	1.7E-11		CAV2,COL1A1,COL1A2,COL3A1,COL5A1,COL5A2,COL6A2,CSRP2,DAB2,KDELR2,LOX	15
Cancer,Gastrointestinal Disease,Organismal Injury and Abnormalities	Digestive organ tumor	2.07E-11	1.854	A2M,AAK1,ACAD8,ACADM,ACAT2,ACP2,ACP6,ACSL1,ADA,ADAM10,ADD3,AGK,AHR,A	476
Cancer,Organismal Injury and Abnormalities	Formation of solid tumor	5.15E-11	1.273	A2M,AAK1,ACAD8,ACADM,ACOT2,ACP2,ACP6,ACSL1,ACY3,ADA,ADAM10,ADD3,AGK	460
Organismal Injury and Abnormalities	Benign lesion	5.92E-11	0.784	ACSL1,AHR,ALB,ALCAM,ALG2,ANKH,ANXA2,APOA1,ARAF,ASCC3,ASH2L,BCL2L1,BL	87
Dermatological Diseases and Conditions,Inflammatory Disease,Inflammatory I	Juvenile dermatomyositis	8.23E-11		CAV2,COL1A1,COL1A2,COL3A1,COL5A1,COL5A2,COL6A2,CSRP2,DAB2,KDELR2,LOX	14
Cancer,Gastrointestinal Disease,Organismal Injury and Abnormalities	Gastrointestinal tumor	1.13E-10	1.781	A2M,AAK1,ACAD8,ACADM,ACP2,ACP6,ACSL1,ADA,ADAM10,ADD3,AGK,AHR,AKT1S1	443
Cancer,Gastrointestinal Disease,Organismal Injury and Abnormalities	Gastrointestinal tract cancer	1.8E-10	2.576	A2M,AAK1,ACAD8,ACADM,ACP2,ACP6,ACSL1,ADA,ADAM10,ADD3,AGK,AHR,AKT1S1	441
Dermatological Diseases and Conditions,Inflammatory Disease,Inflammatory I	Dermatomyositis	3.62E-10		C3,CAV2,COL1A1,COL1A2,COL3A1,COL5A1,COL5A2,COL6A2,CSRP2,DAB2,GBP2,IFI	19
Cancer,Organismal Injury and Abnormalities	Benign solid tumor	3.69E-10	1.437	ACSL1,AHR,ALB,ALCAM,ALG2,ANKH,ANXA2,APOA1,ARAF,ASCC3,ASH2L,BCL2L1,C3	76
Cancer,Gastrointestinal Disease,Organismal Injury and Abnormalities	Intestinal tumor	3.86E-10	1.891	A2M,AAK1,ACAD8,ACADM,ACP2,ACP6,ACSL1,ADA,ADAM10,ADD3,AGK,AHR,AKT1S1	419
Cancer,Gastrointestinal Disease,Organismal Injury and Abnormalities	Large intestine neoplasm	5.38E-10	2.396	A2M,AAK1,ACAD8,ACADM,ACP2,ACP6,ACSL1,ADA,ADAM10,ADD3,AGK,AHR,AKT1S1	418
Inflammatory Disease,Inflammatory Response,Skeletal and Muscular Disorde	Inflammation of muscle	6.1E-10		APOA1,C3,CAV2,COL1A1,COL1A2,COL3A1,COL5A1,COL5A2,COL6A2,CSRP2,DAB2,F	23
Cancer,Gastrointestinal Disease,Organismal Injury and Abnormalities	Gastrointestinal carcinoma	8.33E-10	1.452	A2M,AAK1,ACAD8,ACADM,ACP2,ACP6,ACSL1,ADA,ADAM10,ADD3,AGK,AHR,AKT1S1	426
Cancer,Gastrointestinal Disease,Organismal Injury and Abnormalities	Malignant neoplasm of large intestine	8.49E-10	2.412	A2M,AAK1,ACAD8,ACADM,ACP2,ACP6,ACSL1,ADA,ADAM10,ADD3,AGK,AHR,AKT1S1	416
Organismal Survival	Organismal death	2.67E-09	2.712	ACACA,ACADM,ADA,ADAM10,AHR,AKT2,ALG2,ANKH,ANXA2,APOA1,ARAF,ARID1A,A	148
Organismal Survival	Morbidity or mortality	3.29E-09	2.543	ACACA,ACADM,ADA,ADAM10,AHR,AKT2,ALG2,ANKH,ANXA2,APOA1,ARAF,ARID1A,A	149
Cancer,Gastrointestinal Disease,Organismal Injury and Abnormalities	Large intestine carcinoma	3.43E-09		A2M,AAK1,ACAD8,ACADM,ACP2,ACP6,ACSL1,ADA,ADAM10,ADD3,AGK,AHR,AKT1S1	399
Cancer,Hematological Disease,Immunological Disease,Organismal Injury and	Lymphoma	3.6E-09	0.718	ADA,AHR,AKT2,ANXA11,ANXA2,ANXA4,ANXA6,ARHGAP17,ARID1A,ASCC3,BCAT1,BC	102
Cancer,Gastrointestinal Disease,Organismal Injury and Abnormalities	Large intestine adenocarcinoma	4.78E-09		A2M,AAK1,ACAD8,ACADM,ACP2,ACP6,ACSL1,ADA,ADAM10,ADD3,AGK,AHR,AKT1S1	396
Cancer,Organismal Injury and Abnormalities	Breast or colorectal cancer	5.65E-09	2.184	A2M,AAK1,ACADM,ACP2,ACP6,ACSL1,ADA,ADAM10,ADD3,AKT2,ALB,ALCAM,ANAPC	323
Cancer,Gastrointestinal Disease,Organismal Injury and Abnormalities	Gastrointestinal adenocarcinoma	9.17E-09		A2M,AAK1,ACAD8,ACADM,ACP2,ACP6,ACSL1,ADA,ADAM10,ADD3,AGK,AHR,AKT1S1	406
Connective Tissue Disorders,Dermatological Diseases and Conditions,Develo	Autosomal dominant Ehlers-Danlos syndro	9.23E-09		ALB,COL1A1,COL1A2,COL5A1,COL5A2	5
Cell Death and Survival	Apoptosis	1.12E-08	0.583	A2M,ACACA,ACSL1,ADA,ADAM10,AHR,AKT1S1,AKT2,ALB,ALCAM,ANXA11,ANXA2,AN	161
Cancer,Hematological Disease,Immunological Disease,Organismal Injury and	Non-Hodgkin lymphoma	1.88E-08	0.152	ADA,AHR,AKT2,ANXA11,ANXA2,ANXA4,ANXA6,ARHGAP17,ARID1A,ASCC3,BCAT1,BC	88
Skeletal and Muscular Disorders	Laminopathy	2E-08		CAV2,CCN2,COL1A1,COL1A2,COL3A1,COL5A1,COL5A2,COL6A2,CSRP2,DAB2,KDELI	16
Protein Synthesis	Metabolism of protein	2.52E-08	2.088	AAK1,ADAM10,AHR,ALB,ANAPC5,APOA1,ATG4B,BCL2L1,C3,CAMLG,CAPN1,Casp12,C	84
Dermatological Diseases and Conditions,Organismal Injury and Abnormalities	Burn	3.08E-08		ALB,COL12A1,COL18A1,COL1A1,COL1A2,COL3A1,COL5A1,COL5A2,COL6A2,CYP51A	13
Cellular Function and Maintenance	Cellular homeostasis	7.39E-08	-0.99	A2M,ACP2,ACSL1,ADA,ADAM10,AHR,AKT2,ALB,ANXA2,ANXA6,APOA1,ARFGEF1,ARI	108
Cellular Assembly and Organization	Development of cytoplasm	7.75E-08	-0.036	ACSL1,ANKRD13A,APOA1,ARFGEF1,ATG4B,BCL2L1,CAPN1,CARMIL1,CCN2,CHCHD	45
Cell Death and Survival	Necrosis	8.38E-08	0.813	A2M,AAK1,ACACA,ACSL1,ADA,ADAM10,AHR,AKT1S1,AKT2,ALB,ALCAM,ANXA2,APOA	160
Cell Morphology,Cellular Assembly and Organization	Morphology of collagen fibrils	8.73E-08		COL3A1,COL5A1,COL5A2,LOX,P3H4,PCOLCE,PLOD1	7
Cell Morphology,Cellular Assembly and Organization	Morphology of fibrils	1.02E-07		COL3A1,COL5A1,COL5A2,ITM2B,LOX,P3H4,PCOLCE,PLOD1	8
Dermatological Diseases and Conditions,Organismal Injury and Abnormalities	Ulceration of skin	1.09E-07		AHR,COL12A1,COL18A1,COL1A1,COL1A2,COL3A1,COL5A1,COL5A2,COL6A2,FADS2,	13
Infectious Diseases	Infection by RNA virus	1.11E-07	-1.704	ACSL1,ADA,ADAM10,ALB,ANXA2,APOA1,ARID1A,ARPC1A,ASCC3,ATP6AP2,ATP6V0C	76
Cardiovascular System Development and Function,Organismal Development	Angiogenesis	1.13E-07	-1.487	ADA,AHR,ALCAM,ANXA2,APOA1,C3,CAPN1,CAV2,CCN2,CD276,CD63,CD82,CD9,CDK	74
Connective Tissue Disorders,Metabolic Disease,Organismal Injury and Abnorr	Metabolic bone disease	1.2E-07		ALB,CLCN5,COL1A1,COL1A2,COL3A1,COL5A1,COL5A2,ENPP1,FDPS,GLB1,P3H1,PLC	14
Organismal Injury and Abnormalities,Reproductive System Disease	Benign uterine disease	1.35E-07		AHR,ANXA2,ASH2L,BCL2L1,CAPN6,CLCN5,COL12A1,COL18A1,COL1A1,COL1A2,COL	28
Cellular Compromise,Inflammatory Response	Degranulation of cells	1.37E-07	0.652	A2M,ADAM10,ALB,ANXA2,APOA1,ARMC8,ATP6AP2,ATP6V0C,C3,CAPN1,CD109,CD63	47

Cancer,Organismal Injury and Abnormalities,Reproductive System Disease,Sk	Uterine smooth muscle tumor	1.43E-07		AHR,ANXA2,ASH2L,CAPN6,CLCN5,COL12A1,COL18A1,COL1A1,COL1A2,COL3A1,COL	28
Cancer,Organismal Injury and Abnormalities	Benign connective or soft tissue neoplasm	1.59E-07		AHR,ANXA2,ASH2L,CAPN6,CLCN5,COL12A1,COL18A1,COL1A1,COL1A2,COL3A1,COL	35
Cancer,Organismal Injury and Abnormalities,Reproductive System Disease,Sk	Uterine leiomyoma	1.69E-07		AHR,ANXA2,ASH2L,CAPN6,CLCN5,COL12A1,COL18A1,COL1A1,COL1A2,COL3A1,COL	27
Cellular Movement	Cell movement of tumor cell lines	2.09E-07	-0.217	A2M,ADAM10,AGK,AHR,AKT1S1,AKT2,ALCAM,ANXA2,ARFGEF1,ARID1A,ASH2L,BCKD	78
Dermatological Diseases and Conditions,Organismal Injury and Abnormalities	Psoriasis	2.33E-07		A2M,ADAM10,ANXA2,ARPC1A,BCL2L1,C3,CAMLG,CD63,CNN1,COL1A2,CRABP2,CRIP	45
Cellular Assembly and Organization	Organization of organelle	2.46E-07	0	ACP2,ADAM10,ANXA2,ARFGEF1,ARFIP2,ARHGAP17,ARID1A,ATL2,CAMK1,CARMIL1,(	50
Amino Acid Metabolism,Small Molecule Biochemistry	Catabolism of amino acids	2.66E-07		ACAD8,ALDH4A1,BCAT1,BCKDHA,BCKDK,BLMH,CTH,FAH,GCSH,HIBADH,HIBCH,HSD	15
Cancer,Organismal Injury and Abnormalities	Anogenital cancer	2.8E-07	-0.179	A2M,AAK1,ACAD8,ACADM,ACP2,ACP6,ACSL1,ACY3,ADAM10,ADD3,AGK,AHR,AKT2,A	359
Cellular Assembly and Organization,Cellular Function and Maintenance	Organization of collagen fibrils	2.81E-07		ANXA2,COL1A1,COL1A2,COL3A1,COL5A1,COL5A2,DDR2,LOX,P3H1,P3H4	10
Cell Morphology,Cellular Function and Maintenance	Autophagy	3.06E-07	-0.138	ACP2,ACSL1,AKT2,ALB,ATG4B,ATP6V0C,BCKDHA,BCL2L1,BCL2L13,CAMK1,CAPN1,(	43
Cellular Development,Cellular Growth and Proliferation	Cell proliferation of tumor cell lines	3.67E-07	-0.76	A2M,ACACA,ADAM10,AGK,AHR,AKT1S1,AKT2,ALCAM,ANXA2,ANXA6,ARAF,ARID1A,A	115
Cardiovascular Disease,Hereditary Disorder,Organismal Injury and Abnormalit	Familial vascular disease	3.98E-07		ALB,C3,CCN2,COL1A1,COL3A1,COL5A1,COL5A2,DDX58,ELMO2,ENPP1,F2,F5,IFIH1,I(	26
Cardiovascular System Development and Function,Organismal Development	Vasculogenesis	4.17E-07	-1.963	ADA,AHR,ALCAM,ANXA2,APOA1,C3,CAPN1,CAV2,CCN2,CD276,CD63,CD82,CD9,CDK	62
Amino Acid Metabolism,Small Molecule Biochemistry	Catabolism of branched chain amino acids	4.79E-07		ACAD8,BCAT1,BCKDHA,BCKDK,HIBADH,HIBCH,HSD17B10	7
Developmental Disorder,Hereditary Disorder,Organismal Injury and Abnormalit	Duchenne muscular dystrophy	5.57E-07		CAV2,CCN2,COL1A1,COL1A2,COL3A1,COL5A1,COL5A2,COL6A2,CSRP2,DAB2,HLA-A	16
Cancer,Hematological Disease,Organismal Injury and Abnormalities	Hematologic cancer of cells	6.18E-07	0.786	ACP6,ADA,ADAM10,ADD3,AHR,AKT2,ALB,ANKH,ANXA11,ANXA2,ANXA4,ANXA6,ARAF	128
Cardiovascular System Development and Function	Development of vasculature	6.57E-07	-1.487	ADA,AHR,ALCAM,ANXA2,APOA1,C3,CAPN1,CAV2,CCN2,CD276,CD63,CD82,CD9,CDK	77
Cell Death and Survival	Cell death of tumor cell lines	6.76E-07	0.725	ACACA,ADAM10,AHR,AKT1S1,AKT2,ALB,ALCAM,ANXA2,ARAF,BCAS2,BCL2L1,BCL2L	104
Cancer,Organismal Injury and Abnormalities	Urogenital cancer	6.78E-07	0.329	A2M,AAK1,ACAD8,ACADM,ACP2,ACP6,ACSL1,ACY3,ADAM10,ADD3,AGK,AHR,AKR1E	375
Cardiovascular Disease,Hematological Disease,Organismal Injury and Abnorm	Thrombus	7.65E-07	1.944	ACACA,ADA,AHR,AKT2,ALB,ANXA2,BCL2L1,C3,CAMLG,CAPN1,CCN2,COL1A1,F2,F5,(	23
Cancer,Organismal Injury and Abnormalities	Incidence of tumor	8.27E-07	1.161	A2M,AAK1,ACAD8,ACADM,ACP6,ACSL1,ADA,ADAM10,ADD3,AGK,AHR,AKT2,ALB,ALC	401
Cancer,Organismal Injury and Abnormalities	Genitourinary tumor	8.64E-07	0.474	A2M,AAK1,ACAD8,ACADM,ACP2,ACP6,ACSL1,ACY3,ADAM10,ADD3,AGK,AHR,AKR1E	379
Cell Morphology,Cellular Assembly and Organization	Abnormal morphology of collagen fibrils	9.39E-07		COL3A1,COL5A1,COL5A2,LOX,P3H4,PLOD1	6
Dermatological Diseases and Conditions,Organismal Injury and Abnormalities	Ulcer of lower extremity	1.04E-06		COL12A1,COL18A1,COL1A1,COL1A2,COL3A1,COL5A1,COL5A2,COL6A2,PTGS1,SERP	10
Cancer,Organismal Injury and Abnormalities	Malignant genitourinary solid tumor	1.04E-06	0.329	A2M,AAK1,ACAD8,ACADM,ACP2,ACP6,ACSL1,ACY3,ADAM10,ADD3,AGK,AHR,AKR1E	374
Inflammatory Response,Organismal Injury and Abnormalities	Inflammation of organ	1.06E-06	2.044	ADA,AHR,AKT2,ALB,ALCAM,ANXA4,APOA1,ARFGAP3,ARID1A,ATRX,BCL2L1,BLMH,C	92
Cancer,Hematological Disease,Immunological Disease,Organismal Injury and	Lymphocytic cancer	1.11E-06	0.817	ACP6,ADA,ADAM10,ADD3,AHR,AKT1S1,AKT2,ALB,ANKH,ANXA11,ANXA2,ANXA4,ANX	139
Hereditary Disorder,Neurological Disease,Organismal Injury and Abnormalities	Familial encephalopathy	1.15E-06		A2M,ACADM,ACSL1,ADAM10,ADD3,ALB,ANXA11,ARID1A,ARL3,ARMCX3,ASCC3,ATP(	95
Cancer,Organismal Injury and Abnormalities,Reproductive System Disease	Breast or gynecological cancer	1.17E-06	0.218	AAK1,ACAD8,ACADM,ACP2,ACSL1,ADAM10,ADD3,AGK,AHR,AKT2,ALB,ALCAM,ALDH	312
Immunological Disease	Systemic autoimmune syndrome	1.19E-06	1.455	ACADM,ACSL1,ADA,ADAM10,ALB,ANAPC5,APOA1,ATRX,BCL2L1,C3,C9orf78,CAMLG,	86
Cancer,Hematological Disease,Organismal Injury and Abnormalities	Lymphocytic neoplasm	1.2E-06	0.817	ACP6,ADA,ADAM10,ADD3,AHR,AKT1S1,AKT2,ALB,ANKH,ANXA11,ANXA2,ANXA4,ANX	139
Cancer,Hematological Disease,Immunological Disease,Organismal Injury and	Tumorigenesis of lymphocytes	1.48E-06	0.786	ACP6,ADA,ADAM10,ADD3,AHR,AKT1S1,AKT2,ALB,ANKH,ANXA11,ANXA2,ANXA4,ANX	131
Cell Morphology,Cellular Function and Maintenance	Autophagy of cells	1.62E-06	-0.057	AKT2,ALB,ATG4B,BCKDHA,BCL2L1,BCL2L13,CAMK1,CAPN1,CRYAB,DAPK3,DNAJA3,	36
Cardiovascular System Development and Function,Cell-To-Cell Signaling and	Binding of endothelial cells	1.66E-06	-0.52	ADAM10,AKT2,ALCAM,ANXA2,C3,CD63,COL18A1,F2,F5,IGFBP7,ITGA2,ITGA6,KNG1,K	21
Cancer,Organismal Injury and Abnormalities	Lymphatic system tumor	1.99E-06	0.541	ACP6,ADA,ADAM10,ADD3,AHR,AKT1S1,AKT2,ALB,ANKH,ANXA11,ANXA2,ANXA4,ANX	140
Cancer,Organismal Injury and Abnormalities	Frequency of tumor	2.05E-06	1.072	A2M,AAK1,ACAD8,ACADM,ACP6,ACSL1,ADA,ADAM10,ADD3,AGK,AHR,AKT2,ALB,ALC	392
Carbohydrate Metabolism	Quantity of carbohydrate	2.06E-06	1.256	ACACA,ACADM,ACP6,ACSL1,ADA,AKT2,ALB,APOA1,ATP6AP2,C3,CCN2,CDS2,CLCN5	43
Cancer,Hematological Disease,Immunological Disease,Organismal Injury and	Neoplasia of leukocytes	2.07E-06	1.333	ACP6,ADA,ADAM10,ADD3,AHR,AKT1S1,AKT2,ALB,ANKH,ANXA11,ANXA2,ANXA4,ANX	135
Cancer,Hematological Disease,Immunological Disease,Organismal Injury and	T-cell non-Hodgkin lymphoma	2.08E-06		ADA,ARID1A,BCL2L1,BCL2L13,C3,CAMLG,CAV2,COL12A1,COL1A1,COL1A2,COL3A1,(	41
Cancer,Organismal Injury and Abnormalities	Lymphoreticular neoplasm	2.16E-06	0.856	ACSL1,ADA,AHR,AKT2,ALDH4A1,ANXA11,ANXA2,ANXA4,ANXA6,ARAF,ARHGAP17,AF	148
Infectious Diseases	Replication of virus	2.24E-06	0.201	ADAM10,AHR,ANXA6,ARAF,ATG4B,ATP6AP2,ATP6V0C,ATRX,BCL2L1,Casp12,CNOT7	44
Cancer,Hematological Disease,Organismal Injury and Abnormalities	Mature lymphocytic neoplasm	2.37E-06		ADA,AHR,AKT1S1,ANKH,ANXA11,ANXA2,ANXA4,ANXA6,ARAF,ARHGAP17,ARID1A,AS	104
Cancer,Organismal Injury and Abnormalities	Subcutaneous tumor	2.64E-06		AHR,ANXA2,ASH2L,CAMLG,CAPN6,CLCN5,COL12A1,COL18A1,COL1A1,COL1A2,COL	33
Cancer,Organismal Injury and Abnormalities	Advanced malignant tumor	2.73E-06	-0.192	ADAM10,AKT2,ALCAM,ANXA2,APOA1,ARAF,ARID1A,ASH2L,ATRX,BCAT1,BCL2L1,C3,	76
Cancer,Organismal Injury and Abnormalities	Metastasis	2.95E-06	-0.325	ADAM10,AKT2,ALCAM,ANXA2,APOA1,ARAF,ARID1A,ASH2L,ATRX,BCL2L1,C3,CAMLG	71
Cancer,Hematological Disease,Immunological Disease,Organismal Injury and	Waldenström macroglobulinemia	3.13E-06		ANXA11,ANXA2,ANXA4,ANXA6,BCAT1,CAMLG,CDC27,CHAF1A,CREBBP,ECI2,FADD,I	25
Connective Tissue Disorders,Developmental Disorder,Hereditary Disorder,Org	Autosomal skeletal dysplasia	3.63E-06		ANKH,COL1A1,COL1A2,COL3A1,COL5A1,COL5A2,DDX58,FZD2,GPX4,IFIH1,P3H1,SEF	12
Organismal Injury and Abnormalities	Ulcer	3.68E-06		AHR,COL12A1,COL18A1,COL1A1,COL1A2,COL3A1,COL5A1,COL5A2,COL6A2,F2,FADS	15
Connective Tissue Disorders,Developmental Disorder,Hereditary Disorder,Me	Osteogenesis imperfecta type III	3.77E-06		COL1A1,COL1A2,COL5A2,P3H1,SERPINF1	5
Cancer,Organismal Injury and Abnormalities	Tumorigenesis of epithelial neoplasm	4.09E-06	1.596	A2M,AAK1,ACAD8,ACADM,ACP6,ACSL1,ADA,ADAM10,ADD3,AGK,AHR,AKT2,ALB,ALC	387
Cancer,Hematological Disease,Organismal Injury and Abnormalities	Hematopoietic neoplasm	4.13E-06	1.528	ACP6,ACSL1,ADA,ADAM10,ADD3,AHR,AKT1S1,AKT2,ALB,ALDH4A1,ANKH,ANXA11,AI	181
Protein Degradation,Protein Synthesis	Catabolism of protein	4.19E-06	0.436	AAK1,ADAM10,AHR,ANAPC5,APOA1,ATG4B,BCL2L1,CAMLG,CAPN1,Casp12,CDC27,(	47
Cancer,Organismal Injury and Abnormalities,Reproductive System Disease	Breast cancer	4.19E-06	0.218	AAK1,ACP2,AKT2,ANAPC5,ANXA11,APOA1,ARAF,ARFGEF1,ARID1A,ASH2L,ATAD1,A1	152
Connective Tissue Disorders,Organismal Injury and Abnormalities,Skeletal an	Advanced Dupuytren contracture	4.26E-06		COL12A1,COL18A1,COL1A1,COL1A2,COL3A1,COL5A1,COL5A2,COL6A2	8
Cancer,Organismal Injury and Abnormalities,Reproductive System Disease	Peyronie disease	4.26E-06		COL12A1,COL18A1,COL1A1,COL1A2,COL3A1,COL5A1,COL5A2,COL6A2	8
Connective Tissue Disorders,Developmental Disorder,Hereditary Disorder,Org	Autosomal dominant skeletal dysplasia	4.43E-06		ANKH,COL1A1,COL1A2,COL3A1,COL5A1,COL5A2,DDX58,FZD2,IFIH1,P3H1,SERPINF1	11
Cancer,Hematological Disease,Organismal Injury and Abnormalities	Hematologic cancer	4.44E-06	1.639	ACP6,ACSL1,ADA,ADAM10,ADD3,AHR,AKT1S1,AKT2,ALB,ALDH4A1,ANKH,ANXA11,AI	179
Cancer,Organismal Injury and Abnormalities	Breast or pancreatic cancer	4.71E-06	0.6	AAK1,ACAT2,ACP2,ADAM10,AGK,AHR,AKT2,ALB,ALCAM,ANAPC5,ANXA11,APOA1,AF	246
Cancer,Organismal Injury and Abnormalities	Pelvic tumor	4.91E-06	0.199	A2M,AAK1,ACAD8,ACADM,ACP6,ACSL1,ACY3,ADAM10,ADD3,AGK,AHR,AKR1E2,AKT	342
Infectious Diseases	Replication of RNA virus	5.02E-06	0.38	ADAM10,ANXA6,ARAF,ATG4B,ATP6AP2,ATP6V0C,BCL2L1,Casp12,CNOT7,DDX58,DL(	40
Cancer,Organismal Injury and Abnormalities	Adenoma	5.39E-06	1.026	ACSL1,AHR,ALG2,ANXA2,APOA1,ARAF,ASCC3,BCL2L1,C3,CCNB1,CDC27,CDKN2B,C	39
Drug Metabolism,Lipid Metabolism,Molecular Transport,Small Molecule Bioche	Release of epoprostenol	5.4E-06	1.951	F2,KNG1,PTGS1,SERPINC1	4
Connective Tissue Disorders,Developmental Disorder,Hereditary Disorder,Me	Osteogenesis imperfecta	5.41E-06		COL1A1,COL1A2,COL5A2,FDPS,P3H1,SERPINF1,SPARC	7
Cellular Compromise,Inflammatory Response	Degranulation of granulocytes	5.53E-06		ADAM10,ANXA2,ARMC8,ATP6AP2,ATP6V0C,C3,CAPN1,CD63,CDA,CTSA,F2,FTL,FUC	31
Cancer,Organismal Injury and Abnormalities	Development of malignant tumor	5.56E-06	1.357	A2M,AAK1,ACAD8,ACADM,ACP6,ACSL1,ADA,ADAM10,ADD3,AGK,AHR,AKT2,ALB,ALC	386
Cancer,Hematological Disease,Organismal Injury and Abnormalities	Myeloid or lymphoid neoplasm	5.57E-06	1.159	ACP6,ACSL1,ADA,ADAM10,ADD3,AHR,AKT1S1,AKT2,ALB,ALDH4A1,ANKH,ANXA11,AI	179
Cardiovascular Disease	Aneurysm	5.69E-06		COL1A1,COL3A1,COL5A1,COL5A2,FTL,IGFBP7,IL1RN,LOX,PIK3C2A,PTGS1,S100A4,S	13
Cellular Compromise,Inflammatory Response	Degranulation of phagocytes	5.7E-06	0.726	ADAM10,ALB,ANXA2,ARMC8,ATP6AP2,ATP6V0C,C3,CAPN1,CD63,CD9,CDA,CTSA,F2	36

Connective Tissue Disorders,Developmental Disorder,Hereditary Disorder,Org	Familial skeletal dysplasia	5.79E-06		ALB,ANKH,BPNT2,CCN2,COL1A1,COL1A2,COL3A1,COL5A1,COL5A2,DDR2,DDX58,FD	21
Cancer,Organismal Injury and Abnormalities	Pelvic adenocarcinoma	5.81E-06		A2M,AAK1,ACAD8,ACADM,ACP6,ACSL1,ACY3,ADAM10,ADD3,AGK,AHR,AKT2,ALB,AL	293
Cancer,Organismal Injury and Abnormalities	Genitourinary adenocarcinoma	5.91E-06	1.698	A2M,AAK1,ACAD8,ACADM,ACP6,ACSL1,ACY3,ADAM10,ADD3,AGK,AHR,AKT2,ALB,AL	315
Cellular Movement	Cell movement	6.21E-06	-1.178	A2M,ACACA,ADA,ADAM10,AGK,AHR,AKT1S1,AKT2,ALB,ALCAM,ANXA2,APOA1,ARFG	137
Cancer,Organismal Injury and Abnormalities,Respiratory Disease	Lung carcinoma	6.32E-06	1.673	AAK1,ACAD8,ACAT2,ACOT2,ADAM10,ADD3,AGK,AHR,AKT2,ALCAM,ANAPC5,ANKRD1	204
Cancer,Organismal Injury and Abnormalities	Genitourinary carcinoma	6.38E-06	0.059	A2M,AAK1,ACAD8,ACADM,ACP2,ACP6,ACSL1,ACY3,ADAM10,ADD3,AGK,AHR,AKR1E	353
Dermatological Diseases and Conditions,Endocrine System Disorders,Gastroi	Diabetic foot ulcer disorder	6.57E-06		COL12A1,COL18A1,COL1A1,COL1A2,COL3A1,COL5A1,COL5A2,COL6A2,SERPINC1	9
Cancer,Hematological Disease,Organismal Injury and Abnormalities	Neoplasia of blood cells	6.62E-06	1.459	ACP6,ACSL1,ADA,ADAM10,ADD3,AHR,AKT1S1,AKT2,ALB,ALDH4A1,ANKH,ANXA11,AN	176
Skeletal and Muscular System Development and Function	Morphology of muscle	6.87E-06		ACADM,ADA,AHR,AKT2,ARAF,ATP6V0C,BCL2L1,CAPN6,CAV2,CCN2,CDK9,COL12A1,	40
Organismal Development	Morphology of body cavity	7.23E-06		ACADM,ACP2,ACSL1,ADA,ADAM10,AGK,AHR,AKT2,ALB,APOA1,ARAF,ARID1A,ATP6A	98
Lipid Metabolism,Small Molecule Biochemistry	Synthesis of lipid	7.36E-06	-0.977	A2M,ACACA,ACAT2,ACP6,ACSL1,ADA,AGK,AHR,AKT2,ALB,ALG2,APOA1,BCL2L1,C3,(	57
Cellular Compromise,Inflammatory Response	Degranulation of myeloid cells	7.74E-06	0.8	ADAM10,ALB,ANXA2,ARMC8,ATP6AP2,ATP6V0C,C3,CAPN1,CD63,CD9,CDA,CTSA,F2	36
Cancer,Hematological Disease,Immunological Disease,Organismal Injury and	Peripheral T-cell lymphoma	7.84E-06		ARID1A,C3,CAV2,COL12A1,COL1A2,COL3A1,COL6A2,CREBBP,HDAC6,IFIT3,IGFBP7,I	23
Cancer,Organismal Injury and Abnormalities,Reproductive System Disease	Mammary tumor	7.87E-06	0.749	AAK1,ACP2,AKT2,ANAPC5,ANXA11,APOA1,ARAF,ARFGEF1,ARID1A,ASH2L,ATAD1,A	156
Developmental Disorder	Thoracic hypoplasia	7.96E-06	-1.108	CCN2,CYP51A1,FADD,FBLN1,GNAQ,HELLS,ITGA6,JMJD6,LOX,MECOM,NBN,PBRM1,I	17
Cancer,Organismal Injury and Abnormalities	Pelvic carcinoma	8.32E-06		A2M,AAK1,ACAD8,ACADM,ACP6,ACSL1,ACY3,ADAM10,ADD3,AGK,AHR,AKR1E2,AKT	325
Cancer,Organismal Injury and Abnormalities	Development of carcinoma	8.71E-06	1.894	A2M,AAK1,ACAD8,ACADM,ACP6,ACSL1,ADA,ADAM10,ADD3,AGK,AHR,AKT2,ALB,ALC	382
Cancer,Hematological Disease,Immunological Disease,Organismal Injury and	T-cell malignant neoplasm	8.74E-06		ADA,ARID1A,ATRX,BCL2L1,BCL2L13,C3,CAMLG,CAV2,COL12A1,COL1A1,COL1A2,CO	51
Cellular Compromise,Inflammatory Response	Degranulation of neutrophils	8.98E-06		ADAM10,ANXA2,ARMC8,ATP6AP2,ATP6V0C,C3,CAPN1,CD63,CDA,CTSA,FTL,FUCA1,	30
Cancer,Organismal Injury and Abnormalities	Pelvic cancer	9.18E-06		A2M,AAK1,ACAD8,ACADM,ACP6,ACSL1,ACY3,ADAM10,ADD3,AGK,AHR,AKR1E2,AKT	337
Hereditary Disorder,Organismal Injury and Abnormalities,Skeletal and Muscula	Autosomal dominant myopathy	1.02E-05		CAV2,COL1A1,COL1A2,COL3A1,COL5A1,COL5A2,COL6A2,CRYAB,CSRP2,DAB2,DDR	19
Cellular Assembly and Organization,Cellular Function and Maintenance,Tissue	Formation of actin filaments	1.03E-05	0.395	APOA1,ARFGEF1,CAPN1,CARMIL1,CCN2,CHCHD2,CLASP2,COL18A1,DAB2,DLG1,F2	28
Cellular Assembly and Organization,Tissue Development	Formation of filaments	1.04E-05	-0.201	A2M,APOA1,ARFGEF1,CAPN1,CARMIL1,CCN2,CHCHD2,CLASP2,COL18A1,COL5A1,C	35
Connective Tissue Disorders,Dermatological Diseases and Conditions,Develo	Ehlers-Danlos syndrome	1.09E-05		ALB,COL1A1,COL1A2,COL3A1,COL5A1,COL5A2,PLOD1	7
Cardiovascular Disease,Hereditary Disorder,Organismal Injury and Abnormalit	Familial cardiovascular disease	1.13E-05	-0.318	AGK,ALB,APOA1,C3,CAMLG,CCN2,COL1A1,COL1A2,COL3A1,COL5A1,COL5A2,COX1	38
Embryonic Development,Organismal Development	Development of body trunk	1.16E-05	0.752	ACADM,ADA,ADAM10,AHR,AKT2,ALB,ANXA2,ANXA4,APOA1,ARID1A,ARL3,BCL2L1,B	76
Gene Expression	Expression of RNA	1.17E-05	0.193	A2M,ADA,AHR,AKT2,ANXA4,ARID1A,ARMCX3,ASH2L,ATRX,BCL2L1,CAMK1,CC2D1B,	126
Connective Tissue Disorders,Developmental Disorder,Hereditary Disorder,Me	Familial osteogenesis imperfecta	1.21E-05		COL1A1,COL1A2,COL5A2,P3H1,SERPINF1,SPARC	6
Cancer,Organismal Injury and Abnormalities	Development of benign tumor	1.24E-05	1.091	ACSL1,AHR,ALG2,ANXA2,APOA1,ARAF,ASCC3,BCL2L1,C3,CCNB1,CDC27,CDKN2B,C	45
Cancer,Hematological Disease,Organismal Injury and Abnormalities	Mature T-cell or NK-cell neoplasm	1.26E-05		ADA,ARID1A,BCL2L1,BCL2L13,C3,CAMLG,CAV2,COL12A1,COL1A1,COL1A2,COL3A1,(	40
Cell Morphology,Cellular Assembly and Organization	Morphology of filaments	1.26E-05		COL3A1,COL5A1,COL5A2,CRYAB,ITM2B,KIF4A,LOX,P3H4,PCOLCE,PLOD1,ROCK1	11
Lipid Metabolism,Small Molecule Biochemistry	Metabolism of membrane lipid derivative	0.000013	-1.672	ACACA,ACAT2,ACP6,ACSL1,AGK,APOA1,BCL2L1,CD82,CD9,CDS2,CTSA,CYP51A1,F2	37
Infectious Diseases	Dengue hemorrhagic fever	1.36E-05		ALB,C3,F2,F5,IL1RN,SERPINC1,THBD	7
Infectious Diseases	Infection by coronavirus	1.48E-05	1	ACSL1,ADA,ALB,APOA1,ASCC3,C3,CAMLG,CAV2,CD9,DAB2,DDR2,F2,F5,HBA1/HBA2	28
Connective Tissue Disorders,Dermatological Diseases and Conditions,Develo	Ehlers-Danlos syndrome type VIIA	1.52E-05		COL1A1,COL5A1,COL5A2	3
Cancer,Hematological Disease,Immunological Disease,Organismal Injury and	Plasma cell neoplasm	1.54E-05		AKT1S1,ANXA11,ANXA2,ANXA4,ANXA6,ARID1A,BCAT1,BCL2L1,CAMLG,CCNB1,CDC2	46
Cell Death and Survival	Apoptosis of tumor cell lines	1.67E-05	0.511	ACACA,AHR,AKT1S1,AKT2,ALB,ALCAM,ANXA2,ARAF,BCAS2,BCL2L1,BCL2L13,C3,CA	82
Cellular Movement	Cell movement of melanoma cell lines	1.73E-05	-0.569	ALCAM,CAPN1,CCN2,CD82,CNN1,COL18A1,GBP2,ITGA2,ITGB5,KNG1,LAMA5,LGALS	16
Infectious Diseases	Infection by SARS coronavirus	1.74E-05		ACSL1,ADA,ALB,APOA1,ASCC3,C3,CAMLG,DDR2,F2,F5,HBA1/HBA2,IFIH1,IFIT3,IL1RN	25
Cancer,Organismal Injury and Abnormalities,Reproductive System Disease	Breast or ovarian cancer	1.76E-05	0.218	AAK1,ACP2,ADAM10,AGK,AKT2,ALB,ALCAM,ANAPC5,ANXA11,APOA1,ARAF,ARFGEF	194
Cancer,Hematological Disease,Immunological Disease,Organismal Injury and	Low-grade lymphoma	0.000018		ANXA11,ANXA2,ANXA4,ANXA6,ASCC3,BCAT1,BZW2,CAMLG,CARMIL1,CDC27,CHAF1	46
Cancer,Organismal Injury and Abnormalities,Respiratory Disease	Non-small cell lung carcinoma	1.82E-05	2	AAK1,ACOT2,ADAM10,AGK,AHR,AKT2,ALCAM,ANAPC5,ANKRD13A,ANXA2,ANXA6,AF	138
Organismal Development	Morphology of head	1.83E-05		AHR,AKT2,ANKH,ATRX,BPNT2,Brd4,C3,CCN2,COL18A1,COL1A1,COL5A2,CREBBP,CT	64
Carbohydrate Metabolism	Quantity of monosaccharide	2.04E-05	0.621	ACADM,ACSL1,AKT2,APOA1,ATP6AP2,C3,CLCN5,CPE,CREBBP,CTH,CTHRC1,DCXR,	29
Cellular Movement	Migration of cells	2.13E-05	-1.207	A2M,ACACA,ADA,ADAM10,AGK,AHR,AKT1S1,AKT2,ALB,ALCAM,ANXA2,APOA1,ARFG	123
Cellular Compromise,Inflammatory Response	Degranulation of blood platelets	2.17E-05		A2M,ALB,APOA1,CD109,CD63,CD9,F2,F5,KNG1,LGALS3BP,QSOX1,SPARC,TF	13
Cancer,Organismal Injury and Abnormalities	Thoracic neoplasm	0.000022	1.855	AAK1,ACAD8,ACAT2,ACOT2,ADAM10,ADD3,AGK,AHR,AKT2,ALCAM,ANAPC5,ANKRD1	210
Cancer,Organismal Injury and Abnormalities	Invasive tumor	2.42E-05	-0.273	ADAM10,AKT2,ALCAM,ANXA2,APOA1,ARAF,ARID1A,ASH2L,ATRX,BCL2L1,C3,CAMLG	78
Cancer,Organismal Injury and Abnormalities,Respiratory Disease	Lung tumor	2.46E-05	1.855	AAK1,ACAD8,ACAT2,ACOT2,ADAM10,ADD3,AGK,AHR,AKT2,ALCAM,ANAPC5,ANKRD1	209
Cancer,Organismal Injury and Abnormalities,Reproductive System Disease	Female genital tract serous carcinoma	2.58E-05		ANXA2,ARID1A,ATRX,CCNB1,CDA,CNN1,COL3A1,COL5A1,COL6A2,CRABP1,DAB2,DD	31
Cancer,Organismal Injury and Abnormalities	Pelvic serous carcinoma	2.58E-05		ANXA2,ARID1A,ATRX,CCNB1,CDA,CNN1,COL3A1,COL5A1,COL6A2,CRABP1,DAB2,DD	31
Hematological System Development and Function,Lymphoid Tissue Structure	Morphology of spleen	2.64E-05		ACP2,ADA,AGK,AHR,BCL2L1,Brd4,C3,CDKN2B,CREBBP,CTSA,DDX58,EFEMP1,FADD	29
Cancer,Hematological Disease,Immunological Disease,Organismal Injury and	B-cell lymphoma	2.68E-05	0	ADA,AHR,ANXA11,ANXA2,ANXA4,ANXA6,ARHGAP17,ARID1A,ASCC3,BCAT1,BCL2L1,	64
Neurological Disease	Motor dysfunction or movement disorder	2.68E-05	3.028	A2M,ACADM,ADD3,AHR,ALB,ANKH,ANXA2,ARAF,ARL3,ARMCX3,ATAD1,ATP6AP2,BC	71
Hereditary Disorder,Organismal Injury and Abnormalities	X-linked hereditary disease	0.000027		ATP6AP2,ATRX,C3,CARMIL1,CAV2,CCN2,CLCN5,COL1A1,COL1A2,COL3A1,COL5A1,(	33
Infectious Diseases	COVID-19	2.98E-05		ACSL1,ADA,ALB,APOA1,ASCC3,C3,CAMLG,DDR2,F2,F5,HBA1/HBA2,IFIH1,IFIT3,IL1RN	24
Cellular Movement	Migration of tumor cell lines	0.00003	-0.192	A2M,ADAM10,AGK,AHR,AKT1S1,AKT2,ALCAM,ANXA2,ARFGEF1,ARID1A,ASH2L,BCKI	62
Cancer,Hematological Disease,Immunological Disease,Organismal Injury and	Mature T-cell neoplasm	3.03E-05		ADA,ARID1A,BCL2L1,BCL2L13,C3,CAMLG,CAV2,COL12A1,COL1A1,COL1A2,COL3A1,(	38
Cardiovascular Disease	Vascular lesion	3.11E-05	0.244	ACAT2,AHR,APOA1,COL18A1,COL1A1,COL3A1,COL5A1,COL5A2,F5,FTL,IGFBP7,IL1R	24
Cancer,Organismal Injury and Abnormalities	Intrathoracic malignant tumor	3.12E-05	1.909	AAK1,ACAD8,ACAT2,ACOT2,ADAM10,ADD3,AGK,AHR,AKT2,ALCAM,ANAPC5,ANKRD1	207
Cancer,Gastrointestinal Disease,Organismal Injury and Abnormalities	Colorectal tumor	3.15E-05	2.396	A2M,ACADM,ACP6,ACSL1,ADA,ADAM10,ADD3,AKT2,ALB,ALCAM,ANAPC5,ANXA11,A	264
Cell Death and Survival	Cell survival	3.16E-05	-1.59	A2M,ACACA,AHR,AKT2,ALB,ALCAM,ARID1A,ASCC3,ATG4B,ATRX,BCKDK,BCL2L1,Br	93
Organismal Injury and Abnormalities,Reproductive System Disease	Benign pelvic disease	3.35E-05		AHR,ANXA2,ASH2L,BCAT1,BCL2L1,CAPN6,CAV2,CCN2,CLCN5,COL12A1,COL18A1,C	41
Protein Synthesis	Quantity of interleukin	3.35E-05	0.39	C3,Casp12,HDAC6,IFI16,IL1RN,KRT1,LDLR,LGALS3BP,PLCD1,SCARB1,SLC33A1,STIN	14
Lipid Metabolism,Small Molecule Biochemistry,Vitamin and Mineral Metabolisr	Synthesis of sterol	3.79E-05	0	ACACA,ACAT2,APOA1,CYP51A1,FDPS,HMGCS1,LDLR,MSMO1,MVK,NSDHL,PMVK,SI	12
Lipid Metabolism,Small Molecule Biochemistry,Vitamin and Mineral Metabolisr	Synthesis of cholesterol	3.83E-05		ACACA,ACAT2,APOA1,CYP51A1,FDPS,HMGCS1,LDLR,MSMO1,MVK,NSDHL,PMVK	11
Cancer,Organismal Injury and Abnormalities,Respiratory Disease	Lung cancer	3.84E-05	1.909	AAK1,ACAD8,ACAT2,ACOT2,ADAM10,ADD3,AGK,AHR,AKT2,ALCAM,ANAPC5,ANKRD1	206
Organismal Development	Abnormal morphology of body cavity	4.06E-05		ACADM,ACSL1,ADA,ADAM10,AGK,AHR,AKT2,ARAF,BCL2L1,C3,CAV2,CCN2,CD9,CDK	85

Cancer,Organismal Injury and Abnormalities,Reproductive System Disease	Genital tumor	4.19E-05	-0.271	A2M,AAK1,ACAD8,ACADM,ACP6,ACSL1,ACY3,ADAM10,ADD3,AGK,AHR,AKT2,ALB,AL	322
Endocrine System Disorders,Gastrointestinal Disease,Metabolic Disease,Orga	Non-insulin-dependent diabetes mellitus	4.21E-05		ACACA,AKR1E2,AKT2,ALB,ANAPC5,ANXA11,ANXA2,BCL2L1,CCN2,COL1A1,COL1A2,(	33
Connective Tissue Disorders,Dermatological Diseases and Conditions,Develo	Classic Ehlers-Danlos syndrome	4.28E-05		COL1A1,COL1A2,COL5A1,COL5A2	4
Free Radical Scavenging	Synthesis of reactive oxygen species	4.29E-05	-0.777	AHR,AKT2,ALB,ANXA2,APOA1,ATP6AP2,BCL2L1,C3,COL18A1,CRYAB,CTH,F2,FADD,I	37
Metabolic Disease,Organismal Injury and Abnormalities	Glucose metabolism disorder	0.000043	2.418	A2M,ACACA,ACAT2,ACSL1,AKR1E2,AKT2,ALB,ANAPC5,ANXA11,ANXA2,APOA1,APO(	91
Connective Tissue Disorders,Developmental Disorder,Hereditary Disorder,Org	Marfan syndrome	0.000043		APOA1,CNN1,COL1A2,COL3A1,COL5A1,COL5A2,TGFBR2	7
Cell-To-Cell Signaling and Interaction,Cellular Assembly and Organization,Cel	Formation of focal adhesions	4.47E-05	1.429	CAPN1,CCN2,CHCHD2,CLASP2,COL18A1,DAPK3,F2,ITGA2,KNG1,MAP4K4,PEAK1,PH	16
Organismal Survival	Survival of organism	0.000046	-0.811	ACSL1,AKT2,APOA1,BABAM1,BCL2L1,BLMH,BRCC3,C3,CCN2,CDA,COL18A1,COL3A1	53
Cellular Assembly and Organization,Tissue Development	Fibrogenesis	0.000047	-0.406	A2M,APOA1,ARFGEF1,CAPN1,CARMIL1,CCN2,CHCHD2,CLASP2,COL18A1,COL5A1,C	36
Cancer,Organismal Injury and Abnormalities	Multiple cancers	5.01E-05	0.218	AAK1,ACP2,ADAM10,AGK,AKT1S1,AKT2,ALB,ALCAM,ANAPC5,ANXA11,APOA1,ARAF,	197
Infectious Diseases	Replication of Paramyxoviridae	0.000051	1.178	BCL2L1,CNOT7,HDAC6,IFIH1,RAB9A,STING1,UBE3C	7
Cancer,Organismal Injury and Abnormalities,Reproductive System Disease	Female genital tract adenocarcinoma	5.11E-05		AAK1,ACAD8,ACADM,ACSL1,ADD3,AGK,AHR,AKT2,ALB,ALDH4A1,ALG2,ANKH,ANXA1	234
Cancer,Organismal Injury and Abnormalities	Advanced malignant solid tumor	5.25E-05	0.715	AKT2,ANXA2,APOA1,ARAF,ARID1A,ATRX,BCL2L1,C3,CAMLG,CCN2,CD9,COL18A1,CF	51
Cellular Assembly and Organization,Cellular Function and Maintenance,Tissue	Formation of actin stress fibers	5.25E-05	0.41	APOA1,CAPN1,CARMIL1,CCN2,CHCHD2,CLASP2,COL18A1,DAB2,F2,GNAQ,HDAC6,IT	22
Cardiovascular Disease,Hereditary Disorder,Organismal Injury and Abnormaliti	Familial thoracic aortic aneurysms and diss	5.37E-05		COL1A1,COL3A1,COL5A1,COL5A2,LOX,TGFBR2	6
Cancer,Organismal Injury and Abnormalities	Serous adenocarcinoma	5.51E-05		AKT2,ALB,ANXA2,ARID1A,ATRX,C3,CCNB1,CDA,CDC27,CNN1,COL18A1,COL3A1,COI	60
Molecular Transport,Protein Synthesis,Protein Trafficking	Localization of protein	5.77E-05		AAK1,AKT2,ANXA2,CAMK1,CLASP2,DLG1,EXOC5,FTL,HLA-A,LAMA5,LDLR,MAPRE3,N	23
Lipid Metabolism,Molecular Transport,Small Molecule Biochemistry	Depletion of cholesterol	5.96E-05		APOA1,LDLR,SCARB1	3
Cancer,Organismal Injury and Abnormalities	Mesenchymal tumor	6.29E-05		ATRX,COL12A1,COL18A1,COL1A1,COL1A2,COL3A1,COL5A1,COL5A2,COL6A2,CRYAI	17
Cardiovascular Disease,Hematological Disease,Organismal Injury and Abnorm	Thrombosis of vein	6.33E-05		ADA,ALB,CCN2,F2,F5,LGALS3BP,PTGS1,SERPINC1,THBD	9
Cancer,Organismal Injury and Abnormalities,Respiratory Disease	Respiratory system tumor	6.48E-05	1.751	AAK1,ACAD8,ACAT2,ACOT2,ADAM10,ADD3,AGK,AHR,AKT2,ALB,ALCAM,ANAPC5,ANI	220
Cardiovascular Disease,Organismal Injury and Abnormalities	Arterial aneurysm	6.49E-05		COL1A1,COL3A1,COL5A1,COL5A2,FTL,IGFBP7,LOX,PIK3C2A,S100A4,TGFBR2	10
Hematological System Development and Function,Lymphoid Tissue Structure	Morphology of lymphoid organ	6.49E-05		ACP2,ADA,AGK,AHR,ARAF,ATP6AP2,BCL2L1,Brd4,C3,CDKN2B,CREBBP,CTSA,DDX58	34
Organismal Survival	Perinatal death	6.66E-05	0.609	ADA,ARID1A,BLMH,BPNT2,CCN2,COL12A1,COL5A2,CREBBP,DERL2,DLG1,EIF4E2,F2	33
Free Radical Scavenging	Metabolism of reactive oxygen species	6.76E-05	-0.737	AHR,AKT2,ALB,ANXA2,APOA1,ATP6AP2,BCL2L1,C3,CCN2,COL18A1,CRYAB,CTH,F2,I	38
Cancer,Organismal Injury and Abnormalities,Reproductive System Disease	Genital tract cancer	6.82E-05		A2M,AAK1,ACAD8,ACADM,ACP6,ACSL1,ACY3,ADAM10,ADD3,AGK,AHR,AKT2,ALB,AL	317
Connective Tissue Disorders,Hereditary Disorder,Organismal Injury and Abno	Hereditary connective tissue disorder	7.05E-05		AGK,AKT2,ALB,ANKH,APOA1,BPNT2,C3,CCN2,CLCN5,CNN1,COL12A1,COL1A1,COL1	45
Gene Expression	Transcription of RNA	7.39E-05	-0.129	A2M,AHR,AKT2,ANXA4,ARID1A,ARMCX3,ASH2L,ATRX,BCL2L1,CAMK1,CC2D1B,CCN2	100
Hereditary Disorder,Organismal Injury and Abnormalities,Skeletal and Muscula	Hereditary myopathy	7.48E-05		ACADM,AGK,APOA1,BCL2L1,C3,CAPN1,CAV2,CCN2,COL12A1,COL1A1,COL1A2,COL3	43
Cancer,Neurological Disease,Organismal Injury and Abnormalities	Anaplastic glioma	0.000075		AKT2,ALCAM,ARID1A,ATRX,BCL2L1,BCL2L13,COL1A1,COL1A2,COL3A1,CREBBP,CSI	33
Cancer,Hematological Disease,Immunological Disease,Organismal Injury and	B-cell neoplasm	7.73E-05	0.447	ACP6,ADA,ADAM10,ADD3,AHR,AKT1S1,ALB,ANKH,ANXA11,ANXA2,ANXA4,ANXA6,AR	106
Cardiovascular System Development and Function,Cell-To-Cell Signaling and	Adhesion of endothelial cells	7.95E-05	-0.36	ADAM10,AKT2,ALCAM,CD63,COL18A1,F2,IGFBP7,ITGA6,MYADM,RICTOR,SPARC,ST/	15
Tissue Development	Organization of extracellular matrix	8.01E-05		A2M,ADAM10,CAPN1,COL18A1,COL1A1,COL1A2,COL3A1,COL5A1,COL5A2,COL6A2,D	19
Skeletal and Muscular Disorders,Skeletal and Muscular System Development	Abnormal morphology of muscle	0.000083		ACADM,ARAF,CAPN6,CAV2,COL12A1,CSRP2,DHTKD1,DLG1,ENPP1,FADD,IFRD1,JM.	23
Connective Tissue Disorders,Inflammatory Disease,Organismal Injury and Abi	Rheumatic Disease	8.46E-05	0.603	ACADM,ACSL1,ADA,ADAM10,AHR,ALB,ANKH,APOA1,ATRX,BCL2L1,C3,C9orf78,CAMK	76
Molecular Transport	Transport of molecule	8.51E-05	0.799	A2M,ACACA,ACAT2,ACSL1,ADA,AGK,AKT2,ALB,ANKH,ANXA2,ANXA6,APOA1,ARL3,A	94
Inflammatory Response,Neurological Disease	Inflammation of central nervous system	8.92E-05	2.573	AHR,ALCAM,BCL2L1,C3,CD276,CYP51A1,F5,FDPS,GPX4,GSDMD,HLA-A,HMGCS1,IFI	32
Dermatological Diseases and Conditions,Developmental Disorder,Organismal	Congenital anomaly of skin	8.93E-05		ADAM10,ALB,COL1A1,COL1A2,COL3A1,COL5A1,COL5A2,GNAQ,ITGA6,KRT5,MECOM	16
Inflammatory Response	Inflammation of absolute anatomical region	9.01E-05	1.902	ADA,AHR,AKT2,ALB,ALCAM,ANXA4,APOA1,ARID1A,ATRX,BCL2L1,C3,CAMLG,CD276,	75
Cancer,Hematological Disease,Immunological Disease,Organismal Injury and	Mature B cell malignant tumor	9.39E-05		ADA,AHR,AKT1S1,ANXA11,ANXA2,ANXA4,ANXA6,ARHGAP17,ARID1A,ASCC3,BCAT1,	72
Cellular Movement	Invasion of cells	9.45E-05	-0.29	A2M,ADAM10,AHR,AKT1S1,AKT2,ALCAM,ANXA2,ARID1A,ASH2L,ATP6V0C,BCAT1,BC	68
Cancer,Hematological Disease,Immunological Disease,Organismal Injury and	B cell cancer	9.68E-05	0.447	ACP6,ADA,ADAM10,ADD3,AHR,AKT1S1,ALB,ANXA11,ANXA2,ANXA4,ANXA6,ARHGAP	98
Endocrine System Disorders,Gastrointestinal Disease,Metabolic Disease,Orga	Experimentally-induced diabetes	9.78E-05		A2M,ANXA2,C3,GBP2,HK2,IL1RN,LDLR,PDLIM4,PPT2,SCARB1,SMDT1,TF	12
Developmental Disorder,Hereditary Disorder,Organismal Injury and Abnormali	Progressive muscular dystrophy	0.000101		CAV2,CCN2,COL1A1,COL1A2,COL3A1,COL5A1,COL5A2,COL6A2,CSRP2,DAB2,HLA-A	17
Cardiovascular Disease,Organismal Injury and Abnormalities	Aortic dilatation	0.000104		COL1A1,COL3A1,COL5A1,COL5A2,FTL,GLA,LOX,PIK3C2A,S100A4,TGFBR2	10
Hematological Disease,Hereditary Disorder,Organismal Injury and Abnormaliti	Hereditary thrombophilia	0.000108		F2,F5,SERPINC1,THBD	4
Cancer,Gastrointestinal Disease,Organismal Injury and Abnormalities	Colorectal cancer	0.000108	2.412	A2M,ACADM,ACP6,ACSL1,ADA,ADAM10,ADD3,ALB,ALCAM,ANAPC5,ANXA11,ANXA2,	257
Cellular Assembly and Organization,Cellular Function and Maintenance	Organization of cytoplasm	0.00011	-0.062	A2M,ACACA,ACP2,ADAM10,AHR,ALCAM,ARFGEF1,ARFIP2,ARHGAP17,ARL3,ARPC1A	91
Hematological System Development and Function	Hemostasis	0.000122	0.33	A2M,AKT2,ANXA2,C3,CAPN1,CARMIL1,COL1A1,COL1A2,F2,F5,GNAQ,ITGA2,ITPR3,KI	25
Cellular Movement	Cell movement of colorectal cancer cell line	0.000124	0.97	AKT2,BCKDK,BCL2L1,CAPN1,CD82,EFEMP1,ENPP1,F2,ITGA2,ITGA6,NINJ1,PLCD1,PT	16
Developmental Disorder,Hereditary Disorder,Organismal Injury and Abnormali	Dystrophy of muscle	0.000127		C3,CAV2,CCN2,COL12A1,COL1A1,COL1A2,COL3A1,COL5A1,COL5A2,COL6A2,CSRP2	23
Dermatological Diseases and Conditions,Organismal Injury and Abnormalities	Abnormality of skin morphology	0.000143	0	AHR,C3,COL1A1,COL1A2,COL3A1,COL5A1,CREBBP,CTSA,CYP51A1,DDX58,ENPP1,F	27
Hematological System Development and Function,Organismal Functions	Coagulation of blood	0.000146	-0.585	A2M,ANXA2,C3,CAPN1,CARMIL1,COL1A1,COL1A2,F2,F5,GNAQ,ITGA2,ITPR3,KDM1A,	22
Cardiovascular Disease,Hematological Disease,Hereditary Disorder,Organism	Familial venous thrombosis	0.000146		F2,F5,SERPINC1	3
Connective Tissue Disorders,Organismal Injury and Abnormalities,Skeletal an	Degeneration of intervertebral disc	0.000146		CCN2,NFIX,SPARC	3
Cellular Assembly and Organization	Formation of cytoskeleton	0.000146	0.123	APOA1,ARFGEF1,CAPN1,CARMIL1,CCN2,CHCHD2,CLASP2,COL18A1,DAB2,DLG1,F2	30
Cancer,Hematological Disease,Immunological Disease,Organismal Injury and	Plasma cell dyscrasia	0.00015		AKT1S1,ANXA11,ANXA2,ANXA4,ANXA6,ARID1A,BCAT1,CAMLG,CDC27,CDKN2B,CHA	40
Gene Expression	Transcription	0.000153	0.146	A2M,AHR,AKT2,ANXA4,ARID1A,ARMCX3,ASH2L,ATRX,BCL2L1,C3,CAMK1,CC2D1B,C(	114
Cancer,Organismal Injury and Abnormalities,Reproductive System Disease	Breast adenocarcinoma	0.000153	1.387	AAK1,AKT2,ANAPC5,ARFGEF1,ARID1A,ASH2L,ATRX,BCL2L1,C3,CAPN1,CD109,CDC2	54
Cancer,Organismal Injury and Abnormalities,Reproductive System Disease	Breast carcinoma	0.000154	0.218	AAK1,ACP2,AKT2,ANAPC5,ANXA11,ARAF,ARFGEF1,ARID1A,ASH2L,ATAD1,ATP6V0C	118
Cell Death and Survival	Cell viability	0.000154	-1.275	A2M,ACACA,AHR,AKT2,ALB,ALCAM,ARID1A,ASCC3,ATG4B,ATRX,BCKDK,BCL2L1,C3	86
Cancer,Hematological Disease,Immunological Disease,Organismal Injury and	Mature B-cell neoplasm	0.000156		ADA,AHR,AKT1S1,ANKH,ANXA11,ANXA2,ANXA4,ANXA6,ARAF,ARHGAP17,ARID1A,AS	84
Energy Production,Lipid Metabolism,Small Molecule Biochemistry	Oxidation of fatty acid	0.000158	0.219	ACACA,ACADM,ACSL1,AKT2,APOA1,C3,CPT2,CROT,ECI2,HADH,KDM1A,MTOR,PTGS	17
Connective Tissue Disorders,Developmental Disorder,Hereditary Disorder,Org	Classic Marfan syndrome	0.000158		COL3A1,COL5A1,COL5A2,TGFBR2	4
Cell Morphology	Shape change of neuroglia	0.000158		CD9,F2,ITGA6,ROCK1	4
Connective Tissue Development and Function,Skeletal and Muscular System	Size of bone	0.000159		ACP2,AHR,ANKH,CAPN1,CCN2,COL1A1,COL1A2,CTHRC1,DDR2,GNAQ,IFIH1,IFRD1,I	23
Neurological Disease	Movement Disorders	0.000161	2.418	A2M,ACADM,ADD3,AHR,ALB,ANKH,ANXA2,ARAF,ARL3,ARMCX3,ATAD1,ATP6AP2,BC	67
Embryonic Development,Organ Development,Organismal Development,Respi	Formation of lung	0.000165	-0.896	ADA,CAV2,CCN2,CD9,COL3A1,CREBBP,DLG1,FBLN1,FZD2,JMJD6,LAMA5,LOX,NFIB,I	23

Lipid Metabolism,Molecular Transport,Small Molecule Biochemistry	Concentration of arachidonic acid	0.000169	0.274	ACOT2,ELOVL5,F2,FADS2,KNG1,PTGS1	6
Carbohydrate Metabolism,Molecular Transport,Small Molecule Biochemistry	Concentration of D-glucose	0.000174	0.968	ACADM,ACSL1,AKT2,APOA1,ATP6AP2,C3,CLCN5,CPE,CREBBP,CTHRC1,DCXR,DHTI	26
Dermatological Diseases and Conditions,Inflammatory Disease,Inflammatory I	Dermatitis	0.000175	1.661	ADA,AHR,ARFGAP3,BCL2L1,BLMH,C3,CAMLG,CYP51A1,FADS2,GRSF1,HDAC6,HLA-A	30
Cancer,Hematological Disease,Immunological Disease,Organismal Injury and	B-cell non-Hodgkin lymphoma	0.000175		ADA,AHR,ANXA11,ANXA2,ANXA4,ANXA6,ARHGAP17,ARID1A,ASCC3,BCAT1,BZW2,C/	58
Cancer,Organismal Injury and Abnormalities	Metastatic solid tumor	0.000179	0.715	AKT2,ANXA2,APOA1,ARAF,ARID1A,ATRX,BCL2L1,C3,CAMLG,CCN2,CD9,COL18A1,CF	44
Developmental Disorder	Hypoplasia of organ	0.000179	-0.242	AHR,ATRX,CCN2,CNOT7,CTHRC1,CYP51A1,DLG1,FADD,FBLN1,GNAQ,HELLS,HS2ST	31
Neurological Disease,Organismal Injury and Abnormalities	Progressive encephalopathy	0.000182		A2M,ADAM10,ALB,ALG2,ANXA11,ANXA2,APOA1,BCL2L1,C3,CAPN1,CCN2,CCNB1,CH	59
Cancer,Organismal Injury and Abnormalities	Fibroma	0.000185		COL12A1,COL18A1,COL1A1,COL1A2,COL3A1,COL5A1,COL5A2,COL6A2,DDR2,MTOR	12
Cancer,Organismal Injury and Abnormalities,Reproductive System Disease	Female genital neoplasm	0.000186		AAK1,ACAD8,ACADM,ACSL1,ADAM10,ADD3,AGK,AHR,AKT2,ALB,ALCAM,ALDH4A1,AI	260
Cardiovascular System Development and Function	Morphology of cardiovascular system	0.000191		ACADM,ACSL1,ADA,ADAM10,AHR,ARAF,BCL2L1,CAPN1,CCN2,CDK9,CDS2,CNN1,CC	56
Skeletal and Muscular System Development and Function	Grip strength	0.000196	-1.667	ACSL1,COL12A1,CPE,CREBBP,FUCA1,LDLR,Sacs,SLC33A1,USP19	9
Hematological System Development and Function,Lymphoid Tissue Structure	Morphology of lymphoid tissue	0.000207		ACP2,ADA,AGK,AHR,ARAF,ATP6AP2,BCL2L1,Brd4,C3,CDKN2B,CREBBP,CTSA,CYP51	35
Cellular Movement,Hematological System Development and Function,Immune	Cell movement of leukocytes	0.000214	0.082	ADA,ADAM10,AHR,AKT2,ALB,ALCAM,ANXA2,APOA1,C3,CCN2,CD276,CD63,CD9,COL	53
Cardiovascular Disease,Hereditary Disorder,Organismal Injury and Abnormalit	Familial aortic aneurysm	0.000214		COL3A1,COL5A1,COL5A2,LOX,TGFBR2	5
Cancer,Organismal Injury and Abnormalities	Fibrous tissue tumor	0.000218		COL12A1,COL18A1,COL1A1,COL1A2,COL3A1,COL5A1,COL5A2,COL6A2,DDR2,MTOR	15
Cancer,Neurological Disease,Organismal Injury and Abnormalities	Glioblastoma	0.000218		AKT2,ALCAM,ARID1A,ATRX,BCL2L1,BCL2L13,COL1A1,COL1A2,COL3A1,CREBBP,CSI	31
Free Radical Scavenging	Generation of reactive oxygen species	0.000219	-0.88	ALB,APOA1,ATP6AP2,BCL2L1,CRYAB,F2,HBA1/HBA2,HSD17B10,ITGA6,ITM2B,KNG1,	18
Cancer,Organismal Injury and Abnormalities,Reproductive System Disease	Development of genital tumor	0.00022		AAK1,ACAD8,ACADM,ACSL1,ADAM10,ADD3,AGK,AHR,AKT2,ALB,ALDH4A1,ALG2,ANK	254
Cardiovascular Disease,Organismal Injury and Abnormalities	Thoracic aortic aneurysms and dissections	0.000221		COL1A1,COL3A1,COL5A1,COL5A2,LOX,S100A4,TGFBR2	7
Lipid Metabolism,Small Molecule Biochemistry	Fatty acid metabolism	0.000223	-0.437	ACACA,ACOT2,ACSL1,ADA,ALB,ANXA6,APOA1,CAV2,CD82,CD9,CPT2,CROT,ECI2,EL	40
Drug Metabolism	Catabolism of xenobiotic	0.000224		ACSL1,CRYZ,GSTM1,GSTM5	4
Energy Production,Lipid Metabolism,Small Molecule Biochemistry	Oxidation of lipid	0.000233	-0.282	ACACA,ACADM,ACSL1,AKT2,APOA1,C3,CPT2,CROT,ECI2,HADH,HSD17B10,KDM1A,M	19
Cardiovascular Disease,Organismal Injury and Abnormalities	Peripheral arterial disease	0.000233		ANXA2,APOA1,COL1A1,COL1A2,CTSA,DAB2,DNAJB1,ERAP1,F2,HBA1/HBA2,HIBCH,M	19
Developmental Disorder,Hereditary Disorder,Ophthalmic Disease,Organismal	Familial congenital cataract	0.000235		AGK,CRYAB,FTL,MSMO1,PIK3C2A,SLC33A1	6
Cancer,Organismal Injury and Abnormalities,Reproductive System Disease	Tumorigenesis of reproductive tract	0.000235		AAK1,ACAD8,ACADM,ACSL1,ADAM10,ADD3,AGK,AHR,AKT2,ALB,ALCAM,ALDH4A1,AI	259
Cardiovascular System Development and Function	Morphology of vessel component	0.000235		COL3A1,ENPP1,IL1RN,LDLR,LOX,LTBP1,PLOD1,ROCK1,TAB1	9
Cancer,Organismal Injury and Abnormalities	Development of adenocarcinoma	0.000249	2.008	AAK1,ACAD8,ACADM,ACP6,ACSL1,ADD3,AGK,AHR,AKT2,ALB,ALDH4A1,ALG2,ANAP(	273
Cellular Movement	Migration of epithelial cells	0.000251	0.184	ADAM10,AHR,AKT2,ANXA2,CCN2,CD82,CD9,ELMO2,ITGA2,MAP4K4,MTOR,SERPINF1	13
Cardiovascular System Development and Function,Organismal Development	Formation of blood vessel	0.000255	0.447	APOA1,CCN2,CDKN2B,CREBBP,CTH,IFI16,LTBP1,PIK3C2A,RAB9A,SERPINF1,SNX17,	15
Cellular Movement	Invasion of tumor cell lines	0.00026	-0.445	A2M,ADAM10,AHR,AKT1S1,AKT2,ALCAM,ARID1A,ASH2L,BCKDK,BCL2L1,CCN2,CD82	56
Cardiovascular Disease,Hematological Disease,Organismal Injury and Abnorm	Venous thromboembolism	0.000265		ALB,F2,F5,PTGS1,SERPINC1	5
Developmental Disorder,Hereditary Disorder,Metabolic Disease,Organismal In	Autosomal recessive inborn error of metab	0.000267		ADA,ALB,COL1A2,COL3A1,CYP51A1,FDPS,IFRD1,IL1RN,P3H1,PLOD1,SERPINF1,SPA	13
Endocrine System Disorders,Gastrointestinal Disease,Metabolic Disease,Orga	Diabetes mellitus	0.000267	0.928	A2M,ACACA,ACSL1,AKR1E2,AKT2,ALB,ANAPC5,ANXA11,ANXA2,APOA1,APOO,BCL2I	75
Cancer,Organismal Injury and Abnormalities	Blastoma	0.000267		AKT2,ALCAM,ARID1A,ATRX,BCL2L1,BCL2L13,CDC27,COL1A1,COL1A2,COL3A1,CREE	32
Cancer,Organismal Injury and Abnormalities	Connective or soft tissue tumor	0.000268	1.481	AHR,ANXA2,ARFGEF1,ARID1A,ASH2L,ATP6AP2,ATRX,BCL2L1,CAPN6,CDC27,CDKN2	87
Cellular Assembly and Organization,Cellular Function and Maintenance	Formation of ribosome	0.000274		DHX29,LONP1,MALSU1,MRPL14,MTOR,PES1	6
Cell-To-Cell Signaling and Interaction	Interaction of tumor cell lines	0.000276	-1.016	ADAM10,ADD3,ALCAM,ANXA2,ARID1A,CD82,CD9,DAB2,EFEMP1,F2,HLA-A,ITGA2,ITG	30
Cardiovascular Disease,Organismal Injury and Abnormalities	Ischemia of heart	0.000276		ADA,AHR,ALB,APOA1,BCL2L1,C3,CAMLG,F2,F5,GSTM1,IL1RN,MME,PTGS1,SERPINC	16
Cell Death and Survival	Apoptosis of pancreatic cancer cell lines	0.000283	0.772	ACACA,ALB,BCL2L1,C3,CPE,CTH,DDX58,EFEMP1,EXOC2,FADD,MTOR,NMI,S100A4	13
Cardiovascular System Development and Function,Organismal Development	Development of artery	0.000283		CDKN2B,COL18A1,CTH,IFI16,LDLR,LOX,LTBP1,RAB9A,RBPJ,SNX17,TAB1,TGFBR2,TU	13
Cardiovascular Disease,Hematological Disease,Organismal Injury and Abnorm	Thromboembolism	0.000283		ADA,ALB,F2,F5,GNAQ,PTGS1,SERPINC1	7
Cancer,Endocrine System Disorders,Organismal Injury and Abnormalities,Rep	Serous ovarian adenocarcinoma	0.000286		ARID1A,ATRX,COL5A1,COL6A2,DAB2,DHTKD1,GSTM5,LOX,MECOM,MTOR,PAPSS2,F	19
Cardiovascular Disease,Organismal Injury and Abnormalities,Tissue Morpholo	Area of fatty lesion	0.000287		LDLR,PON3,TGFBR2	3
Organismal Injury and Abnormalities,Reproductive System Disease	Placental insufficiency	0.000287		F2,PTGS1,SERPINC1	3
Cancer,Dermatological Diseases and Conditions,Hereditary Disorder,Organisn	Familial porokeratosis	0.000287		FDPS,MVK,PMVK	3
Cardiovascular Disease,Hematological Disease,Neurological Disease,Organis	Cerebral venous thrombosis	0.000287		F2,F5,SERPINC1	3
Connective Tissue Disorders,Hematological Disease,Organismal Injury and Al	Heparin-induced thrombocytopenia	0.000287		F2,PTGS1,SERPINC1	3
Cardiovascular Disease,Organismal Injury and Abnormalities,Tissue Morpholo	Surface area of atherosclerotic lesion	0.000287		ACAT2,APOA1,LDLR	3
Organismal Injury and Abnormalities,Reproductive System Disease	Abnormal growth in endometrium	0.000302		AAK1,ACAD8,ACADM,ACSL1,ADD3,AGK,AHR,AKT2,ALB,ALDH4A1,ALG2,ANKH,ANXA1	213
Cell-To-Cell Signaling and Interaction,Hematological System Development an	Aggregation of blood platelets	0.000303	-1.491	AKT2,ALB,C3,CAPN1,CD63,CD9,DAB2,F2,FADS2,GNAQ,ITGA2,KNG1,LOX,PTGS1,SEF	17
Organismal Development	Formation of vessel	0.000304	0.447	AHR,APOA1,CCN2,CDKN2B,CREBBP,CTH,IFI16,LTBP1,PIK3C2A,RAB9A,SERPINF1,SN	16
Cancer,Organismal Injury and Abnormalities,Reproductive System Disease	Female genital tract cancer	0.000305		AAK1,ACAD8,ACADM,ACSL1,ADAM10,ADD3,AGK,AHR,AKT2,ALB,ALCAM,ALDH4A1,AI	254
Cardiovascular Disease,Organismal Injury and Abnormalities	Aortic aneurysm	0.000307		COL1A1,COL3A1,COL5A1,COL5A2,FTL,LOX,PIK3C2A,S100A4,TGFBR2	9
Lipid Metabolism,Molecular Transport,Small Molecule Biochemistry	Uptake of cholesterol ester	0.000308	-0.8	A2M,APOA1,LRPAP1,SCARB1	4
Lipid Metabolism,Molecular Transport,Small Molecule Biochemistry	Concentration of eicosanoid	0.000311	1.212	ACOT2,AHR,ELOVL5,F2,FADS2,GNAQ,IL1RN,KNG1,LDLR,LRPAP1,PTGS1,TGFBR2	12
Inflammatory Response	Immune response of brain	0.000311	2.88	AHR,ALCAM,BCL2L1,C3,CD276,CYP51A1,FDPS,GSDMD,HLA-A,HMGCS1,IFI35,IL1RN,	29
Cancer,Organismal Injury and Abnormalities,Reproductive System Disease	Breast or ovarian carcinoma	0.000313	0.218	AAK1,ACP2,AGK,AKT2,ALB,ANAPC5,ANXA11,ARAF,ARFGEF1,ARID1A,ASH2L,ATAD1,	162
Cancer,Organismal Injury and Abnormalities,Skeletal and Muscular Disorders	Muscle tumor	0.000318		AHR,ANXA2,ASH2L,CAPN6,CDC27,CLCN5,CLPP,COL12A1,COL18A1,COL1A1,COL1A2	44
Organismal Injury and Abnormalities,Reproductive System Disease	Abnormality of endometrium	0.00032		AAK1,ACAD8,ACADM,ACSL1,ADD3,AGK,AHR,AKT2,ALB,ALDH4A1,ALG2,ANKH,ANXA1	213
Cancer,Organismal Injury and Abnormalities,Reproductive System Disease	Endometrial cancer	0.000324		AAK1,ACAD8,ACADM,ACSL1,ADD3,AGK,AHR,AKT2,ALB,ALDH4A1,ALG2,ANKH,ANXA1	212
Cancer,Neurological Disease,Organismal Injury and Abnormalities	Grade 3-4 glioma cancer	0.000326		A2M,AAK1,ACAD8,ACADM,ACOT2,ACP2,ACSL1,ACY3,ADA,ADAM10,AHR,AKT2,ALB,A	252
Connective Tissue Development and Function,Skeletal and Muscular System	Morphology of bone	0.000327		ACP2,AHR,ANKH,BPNT2,CAPN1,CCN2,COL12A1,COL18A1,COL1A1,COL1A2,CREBBP	36
Cancer,Organismal Injury and Abnormalities	Metastasis of cells	0.000331	-0.178	AKT2,ALCAM,ARID1A,ASH2L,CD82,CNN1,COL18A1,CRYAB,CTHRC1,DDR2,EFEMP1,F	28
Skeletal and Muscular Disorders	Abnormality of limb	0.000338	0.816	BPNT2,CCN2,CD276,COL12A1,COL18A1,COL1A1,COL1A2,COL3A1,COL5A1,COL5A2,(	26
Developmental Disorder,Neurological Disease	Congenital neurological disorder	0.000344	0.93	A2M,ACADM,ADAM10,ALB,ALG2,APOA1,ARID1A,ARL3,CCN2,CLPP,COL18A1,COL1A1	51
Cellular Movement	Cell movement of breast cancer cell lines	0.000351	-1.027	ADAM10,AHR,ANXA2,ARID1A,CCN2,CD9,DDR2,DNAJA3,HSBP1,ITGA2,ITGA6,ITGB5,J	27
Developmental Disorder	Hypoplasia	0.000365	-0.029	AHR,ATRX,BPNT2,CCN2,CNOT7,CTHRC1,CYP51A1,DLG1,FADD,FADS2,FBLN1,GNAQ	33
Cancer,Gastrointestinal Disease,Hepatic System Disease,Organismal Injury a	Bile duct adenocarcinoma	0.000369		ALB,ANXA2,ARID1A,CDC27,COL18A1,COL6A2,CRLF3,HDAC6,HLA-A,KNG1,MYH14,MY	20

Organismal Injury and Abnormalities	Organ Degeneration	0.000378	1.846	ACADM,AHR,AKT2,ARL3,ATP6AP2,BCL2L1,C3,CAPN1,CCN2,CD9,CNOT7,COL18A1,D	38
Cancer,Organismal Injury and Abnormalities	Metastasis of melanoma cell lines	0.000386	0.52	AKT2,CNN1,COL18A1,CRYAB,GRSF1,ITGA2,LAMA5,NME1,PTPN14,SERPINF1	10
Neurological Disease	Neurodegeneration	0.000393	2.272	BCL2L1,C3,CAPN1,CREBBP,DHTKD1,F2,FTL,FUCA1,GLB1,GPX4,HDAC6,HIBCH,IL1RN	24
Lipid Metabolism,Small Molecule Biochemistry	Metabolism of glycosphingolipid	0.000396	-1.718	APOA1,CD82,CD9,CTSA,GLA,GLB1,GM2A,SMPD4,SUMF2	9
Lipid Metabolism,Small Molecule Biochemistry,Vitamin and Mineral Metabolism	Metabolism of cholesterol	0.000398	-0.497	ACACA,ACAT2,APOA1,CYP51A1,FDPS,HMGCS1,LDLR,MSMO1,MVK,NSDHL,PMVK,SC	12
Cell Morphology,Cellular Function and Maintenance	Macroautophagy	0.000398	1.007	ATG4B,HDAC6,MTOR,PIK3C2A,POLDIP2,QSOX1,RAB9A,ROCK1,SCOC,STING1,UBXN	13
Cardiovascular System Development and Function,Tissue Morphology	Morphology of artery	0.000421		AHR,COL3A1,COL5A1,JMJD6,KNG1,LDLR,LOX,LTBP1,PLOD1,PTGS1,RBPJ,TAB1,Tms	13
Cardiovascular Disease,Organismal Injury and Abnormalities	Disorder of coronary artery	0.000421		ACAT2,ADA,AHR,APOA1,COL5A2,ENPP1,F2,FADS1,HBA1/HBA2,KDM1A,LDLR,LOX,LF	23
Cell Death and Survival,Organismal Injury and Abnormalities	Necrosis of epithelial tissue	0.000425	0.407	AAK1,AHR,ARID1A,ATRX,BCL2L1,Casp12,COL18A1,CRYAB,CTH,DAB2,F2,FADD,FAH,	40
Cardiovascular Disease,Organismal Injury and Abnormalities	Coronary occlusion	0.00043		ACAT2,ADA,APOA1,COL5A2,ENPP1,F2,FADS1,HBA1/HBA2,KDM1A,LDLR,LOX,LRPAP	18
Cancer,Gastrointestinal Disease,Hepatic System Disease,Organismal Injury a	Biliary tract adenocarcinoma	0.000433		ALB,ANXA2,ARID1A,ATRX,CDC27,COL12A1,COL18A1,COL6A2,CRLF3,HDAC6,HLA-A,	22
Cardiovascular Disease,Organismal Injury and Abnormalities	Formation of blood clot	0.000445		AKT2,ANXA2,C3,F2,PIK3C2A,SERPINC1,THBD,TWF2	8
Cellular Movement	Migration of melanoma cell lines	0.000448	-0.304	ALCAM,CCN2,CD82,COL18A1,ITGA2,ITGB5,LAMA5,LGALS3BP,MAP4K4,NME1,PTPN1	12
Lipid Metabolism,Small Molecule Biochemistry	Esterification of lipid	0.000451	-1.476	ACAT2,ACSL1,APOA1,C3,F2,LDLR,SCARB1	7
Skeletal and Muscular Disorders	Progressive myopathy	0.00046		CAV2,CCN2,COL1A1,COL1A2,COL3A1,COL5A1,COL5A2,COL6A2,CSRP2,DAB2,DHTK	20
Cancer,Organismal Injury and Abnormalities,Respiratory Disease	Development of lung tumor	0.000466	2.157	AAK1,AGK,AHR,AKT2,ANAPC5,ANKRD13A,ARAF,ARFGEF1,ARID1A,ATRX,BCKDHA,B	103
Neurological Disease	Progressive neurological disorder	0.000475	-0.747	A2M,ADAM10,ALB,ALCAM,ALG2,ANXA11,ANXA2,APOA1,BCL2L1,C3,CAPN1,CCN2,CC	65
Hematological System Development and Function,Immunological Disease,Lyn	Abnormal morphology of spleen	0.000479		AGK,AHR,C3,CDKN2B,CREBBP,CTSA,DDX58,EFEMP1,FADS2,GIT2,GPX7,Hbb-b1,HEL	22
Infectious Diseases	Infection of cells	0.000486	-1.929	ADAM10,ANXA2,ARID1A,ARPC1A,ATP6AP2,ATP6V0C,C3,CAMK1,CAPN6,CAV2,CHAF	47
Gastrointestinal Disease,Hepatic System Disease,Metabolic Disease,Organism	Microvesicular hepatic steatosis	0.000488	2	ACADM,ACSL1,AHR,CPT2,CTHRC1,LDLR	6
Cell Cycle,Cellular Movement	Delay in cytokinesis	0.000493		CCNB1,FADD,MYH14	3
Organ Morphology	Regeneration of intestine	0.000493		ELP3,HGFAC,TGFBR2	3
Developmental Disorder	Growth failure or short stature	0.000496	1.36	ACACA,AGK,AHR,ANKH,ATRX,BCL2L1,BPNT2,CCN2,CDKN2B,COL1A1,CREBBP,DDR2	41
Skeletal and Muscular System Development and Function	Contraction of smooth muscle	0.000505	0.97	ADA,C3,CNN1,DAPK3,ITGA2,KNG1,PIK3C2A,PTGS1,ROCK1	9
Respiratory System Development and Function	Respiratory system development	0.000506	-0.478	ADA,AHR,CAV2,CCN2,CD9,COL3A1,CREBBP,DLG1,FBLN1,FZD2,JMJD6,LAMA5,LOX,N	24
Cancer,Organismal Injury and Abnormalities	Visceral metastasis	0.000524	0.314	AKT2,ANXA2,CAMLG,CCN2,CD9,CRABP2,CRYZ,CYP51A1,DDR2,EFEMP1,ELP3,F2,FD	29
Digestive System Development and Function	Morphology of digestive system	0.000528		ACP2,ADA,AHR,AKT2,ANKH,APOA1,ARAF,ARID1A,Brd4,C3,COL3A1,CREBBP,CTHRC	43
Protein Synthesis	Metabolism of cellular protein	0.000529		ADAM10,ALB,APOA1,C3,F2,F5,IGFBP7,ITIH2,ITM2B,KNG1,LAMB2,LTBP1,QSOX1,SER	15
Cell Cycle	M phase	0.000533	0.369	BCL2L1,CCNB1,CDC27,CKAP2,DCTN3,FADD,GOLGA2,KIF20A,KIF4A,KLHL22,MAPRE3	19
Cardiovascular Disease,Hematological Disease,Organismal Injury and Abnorm	Deep vein thrombosis	0.000538		F2,F5,PTGS1,SERPINC1	4
Cardiovascular Disease,Connective Tissue Disorders,Developmental Disorder	Loeys-Dietz syndrome	0.000538		COL3A1,COL5A1,COL5A2,TGFBR2	4
Lipid Metabolism,Molecular Transport,Small Molecule Biochemistry	Secretion of phospholipid	0.000538		ACAT2,AGK,APOA1,LDLR	4
Cancer,Endocrine System Disorders,Organismal Injury and Abnormalities	Benign neoplasm of endocrine gland	0.000553		ACSL1,ALG2,ANKH,ANXA2,APOA1,ARAF,C3,COL1A1,COL1A2,COL6A2,EXOC2,IFIT1B	19
Lipid Metabolism,Small Molecule Biochemistry	Conversion of fatty acid	0.00056	-0.406	ACACA,ACSL1,ALB,CROT,FADS1,MAP4K4,PTGS1	7
Embryonic Development,Organismal Development	Development of head	0.000564	-0.243	ADAM10,ALG2,ARID1A,ARL3,ATP6AP2,ATRX,C3,CLCN5,COL18A1,COL1A1,COL3A1,C	59
Cancer,Organismal Injury and Abnormalities,Reproductive System Disease	Endometrial carcinoma	0.000565		AAK1,ACAD8,ACADM,ACSL1,ADD3,AGK,AHR,AKT2,ALB,ALDH4A1,ALG2,ANKH,ANXA1	204
Respiratory System Development and Function	Compliance of respiratory system	0.000565		ADA,GIT2,Gm15807/Hmgn5,PTGS1,SELENON	5
Cell Morphology,Cellular Movement	Cell spreading	0.000581	0.363	A2M,ALB,C3,CD9,CRYAB,DAB2,EXOC5,F2,GBP2,GIT2,ITGA2,ITGA6,KNG1,MAPRE3,M	21
Post-Translational Modification	Phosphorylation of protein	0.000596	0.956	AAK1,ADAM10,AHR,AKT2,ANXA2,APOA1,ARAF,BCKDK,Brd4,C3,CAMK1,CCN2,CCNB1	42
Cardiovascular System Development and Function,Tissue Development	Development of cardiovascular tissue	0.000604	-2.327	ANXA2,APOA1,C3,CAV2,CD276,CD9,COL18A1,COL1A1,COL1A2,CTH,DAB2,DLG1,ERA	27
Organismal Injury and Abnormalities	Fibrosis	0.00061	0.473	ACADM,ACSL1,ADA,ADAM10,AHR,AKT2,ALB,APOA1,ARHGAP17,BCL2L1,BLMH,Brd4,C	45
Embryonic Development,Organismal Development	Development of body axis	0.000612	-0.243	ADAM10,ALG2,ARID1A,ARL3,ATP6AP2,ATRX,C3,CLCN5,COL18A1,COL1A1,COL3A1,C	62
Connective Tissue Disorders,Hematological Disease,Organismal Injury and Al	Hemoglobin H disease	0.000614		ATRX,HBA1/HBA2	2
Cardiovascular Disease,Hematological Disease,Neurological Disease,Organis	Idiopathic cerebral vein thrombosis	0.000614		F2,F5	2
Connective Tissue Disorders,Dermatological Diseases and Conditions,Develo	Ehlers-Danlos syndrome type II	0.000614		COL5A1,COL5A2	2
Cell Cycle	Replication of Toxoplasma gondii ME49	0.000614		GBP2,STING1	2
Cell Death and Survival,Nervous System Development and Function	Cell viability of dorsal root ganglion cells	0.000614		A2M,LRPAP1	2
Infectious Diseases	Internalization of Human rhinovirus 2	0.000614		LDLR,LRPAP1	2
Connective Tissue Disorders,Developmental Disorder,Hereditary Disorder,Me	Osteogenesis imperfecta type IVB	0.000614		COL1A1,COL1A2	2
Connective Tissue Disorders,Dermatological Diseases and Conditions,Develo	OI/EDS combined syndrome	0.000614		COL1A1,COL1A2	2
Cardiovascular Disease,Hematological Disease,Organismal Injury and Abnorm	Central venous catheter-associated thromb	0.000614		F2,F5	2
Cardiovascular Disease,Connective Tissue Disorders,Developmental Disorder	Singleton-Merten syndrome	0.000614		DDX58,IFIH1	2
Connective Tissue Disorders,Dermatological Diseases and Conditions,Develo	Ehlers-Danlos syndrome type VIIB	0.000614		COL1A1,COL1A2	2
Cellular Growth and Proliferation	Inhibition of carcinoma cell lines	0.000614		BCL2L1,IGFBP7	2
Cellular Assembly and Organization,Cellular Function and Maintenance	Formation of monosome	0.000614		MALSU1,MRPL14	2
Cell Morphology,Cellular Assembly and Organization	Morphology of recycling endosomes	0.000614		ANXA2,EXOC5	2
Cardiovascular Disease,Organismal Injury and Abnormalities	Fibrosis of portal vein	0.000614		AHR,IL1RN	2
Connective Tissue Disorders,Developmental Disorder,Hereditary Disorder,Me	Dominant perinatal lethal osteogenesis imp	0.000614		COL1A1,COL1A2	2
Hereditary Disorder,Organismal Injury and Abnormalities	Turner syndrome	0.00063		ARMCX3,CLCN5,FAM50A,GLA,ITGB5,LAMB2,LTBP1,MAGED1,MED14,MOSPD2,RP2	11
Inflammatory Disease,Inflammatory Response,Neurological Disease,Organism	Encephalitis	0.000644	2.705	AHR,ALCAM,BCL2L1,C3,CD276,CYP51A1,FDPS,GSDMD,HLA-A,HMGCS1,IFI35,IL1RN,	28
Cancer,Organismal Injury and Abnormalities,Reproductive System Disease	Uterine tumor	0.000645		AAK1,ACAD8,ACADM,ACSL1,ADAM10,ADD3,AGK,AHR,AKT2,ALB,ALDH4A1,ALG2,ANK	225
Cancer,Organismal Injury and Abnormalities,Reproductive System Disease	Prostatic tumor	0.000651	0	A2M,AAK1,ACAD8,ACADM,ACP6,ACY3,ADAM10,ADD3,AKT2,ALB,ALDH4A1,ANAPC5,A	176
Cancer,Gastrointestinal Disease,Organismal Injury and Abnormalities	Hepatobiliary carcinoma	0.000655	0.102	A2M,ACSL1,ADA,ADAM10,AGK,AHR,ALB,ALCAM,ALDH4A1,ANAPC5,ANXA2,APOA1,A	217
Skeletal and Muscular System Development and Function	Muscle contraction	0.000664	0.726	ADA,ANXA6,C3,CCN2,CNN1,CRYAB,DAPK3,GNAQ,HSBP1,IFRD1,ITGA2,ITGB5,KNG1,	19
Cellular Assembly and Organization,Cellular Function and Maintenance	Formation of vesicles	0.000664	0.629	ANXA2,ATG4B,C3,CAV2,CDC27,DNAJA3,MTOR,MYH14,RAB11A,RAB34,ROCK1,VPS2	14
Lipid Metabolism,Small Molecule Biochemistry	Synthesis of thromboxane	0.000669		ACACA,F2,LDLR,PTGS1,TGFBR2	5
Cancer,Gastrointestinal Disease,Organismal Injury and Abnormalities	Colorectal carcinoma	0.000674		A2M,ACADM,ACP6,ACSL1,ADA,ADAM10,ADD3,ALB,ANAPC5,ANXA11,ANXA4,ARAF,AI	227
Neurological Disease,Organismal Injury and Abnormalities,Psychological Diso	Tauopathy	0.000685		A2M,ADAM10,ALB,ALG2,APOA1,BCL2L1,C3,CAPN1,CCN2,CCNB1,CD63,CD9,CNN1,CI	39

Connective Tissue Disorders,Developmental Disorder,Organismal Injury and A	Kyphosis	0.000688	2.333	ACP2,CLCN5,COL12A1,COL5A2,EFEMP1,HELLS,MTOR,NFIX,SELENON	9
Protein Synthesis	Termination of translation of protein	0.000688		MRPL14,MRPL16,MRPL2,MRPL24,MRPS16,MRPS17,MRPS23,MRPS24,MRPS25	9
Amino Acid Metabolism,Small Molecule Biochemistry	Catabolism of sulfur amino acid	0.000689		BLMH,CTH,GCSH,MPST	4
Cell Morphology,Cellular Function and Maintenance	Autophagy of tumor cell lines	0.000695	-0.017	AKT2,ALB,BCL2L1,CAMK1,CAPN1,CRYAB,DNAJA3,EXOC2,FADD,HDAC6,HK2,MTOR,I	15
Embryonic Development,Organismal Development	Gastrulation	0.000697		ARID1A,CLASP2,DAB2,KDM1A,LAMA5,MTOR,PHLDB2,TGFBR2	8
Cancer,Gastrointestinal Disease,Organismal Injury and Abnormalities	Tumorigenesis of gastrointestinal tumor	0.000697		A2M,ACADM,ACP6,ACSL1,ADA,ADAM10,ADD3,ALB,ANAPC5,ANXA11,ANXA4,ARAF,AI	228
Cancer,Organismal Injury and Abnormalities,Reproductive System Disease	Prostate cancer	0.000702		A2M,AAK1,ACAD8,ACADM,ACP6,ACY3,ADAM10,ADD3,AKT2,ALB,ALDH4A1,ANAPC5,A	175
Cancer,Endocrine System Disorders,Organismal Injury and Abnormalities,Rep	Ovarian serous tumor	0.000704		AKT2,ALB,ARID1A,ATRX,C3,CDC27,COL18A1,COL3A1,COL5A1,COL6A2,DAB2,DHTKD	47
Cardiovascular System Development and Function,Cell-To-Cell Signaling and	Binding of vascular endothelial cells	0.000707	-1.137	ADAM10,ALCAM,CD63,F2,IGFBP7,ITGA6,KNG1,KRT1,RICTOR,SPARC,STAT6,STX4	12
Cell Morphology,Cellular Function and Maintenance	Macroautophagy of cells	0.000711	1	ATG4B,MTOR,PIK3C2A,POLDIP2,QSOX1,RAB9A,ROCK1,SCOC,STING1,UBXN6,VPS2	11
Cancer,Organismal Injury and Abnormalities	Growth of carcinoma	0.000714	-1.555	ADAM10,APOA1,CCN2,FNTB,HDAC6,Ly6a (includes others),MME,MTOR,RBPJ,STAT5A	13
Protein Synthesis	Quantity of cytokine	0.000714	1.187	AKT2,C3,Casp12,CPE,HDAC6,IFI16,IL1RN,KRT1,LDLR,LGALS3BP,PLCD1,PLSCR3,SC	18
Drug Metabolism,Protein Synthesis	Metabolism of glutathione	0.000719		CTH,Gm15807/Hmgn5,GPX4,GSTM1,GSTM5,Gstt3	6
Cancer,Gastrointestinal Disease,Organismal Injury and Abnormalities	Colorectal adenocarcinoma	0.000721		A2M,ACADM,ACP6,ACSL1,ADA,ADAM10,ADD3,ALB,ANAPC5,ANXA11,ARAF,ARFGAP	222
Cancer,Gastrointestinal Disease,Organismal Injury and Abnormalities	Development of digestive organ tumor	0.000727	1.703	A2M,AAK1,ACADM,ACP6,ACSL1,ADA,ADAM10,ADD3,AHR,AKT2,ALB,ALCAM,ANAPC5	285
Cellular Movement	Cell movement of epithelial cells	0.000731	-0.173	ADAM10,AHR,AKT2,ANXA2,CCN2,CD82,CD9,ELMO2,ITGA2,MAP4K4,MTOR,SERPINF1	14
Cancer,Organismal Injury and Abnormalities	Advanced extracranial solid tumor	0.000744	0.2	AKT2,ANXA2,ARAF,ARID1A,BCL2L1,CAMLG,CCN2,CD9,CRABP2,CRYZ,DAB2,DDR2,D	39
Cellular Assembly and Organization,Cellular Function and Maintenance	Organization of cytoskeleton	0.000744	-0.062	A2M,ACACA,ADAM10,AHR,ALCAM,ARFGEF1,ARFIP2,ARHGAP17,ARL3,ARPC1A,ATR	80
Endocrine System Disorders,Gastrointestinal Disease,Inflammatory Disease,I	Inflammation of pancreas	0.000751	0.684	AHR,ALB,ANXA4,APOA1,F2,F5,MME,NINJ1,NMI,PTGS1,RIDA,STXBP3,TGFBR2	13
Cardiovascular Disease,Cardiovascular System Development and Function,O	Abnormal morphology of artery	0.000755		AHR,COL3A1,COL5A1,JMJD6,LDLR,LOX,LTBP1,PLOD1,RBPJ,TAB1,Tmsb4x (includes c	11
Organismal Development,Organismal Injury and Abnormalities	Abnormal morphology of abdomen	0.000755		ADA,AGK,AHR,AKT2,ARAF,C3,CD9,CDKN2B,COL18A1,COL3A1,CREBBP,CTHRC1,CT	53
Cancer,Endocrine System Disorders,Organismal Injury and Abnormalities	Aldosterone producing adrenocortical aden	0.000759		ACSL1,ALG2,COL1A1,COL1A2,COL6A2,PAPSS2,PCOLCE,SCARB1	8
Organ Morphology,Renal and Urological System Development and Function	Quantity of kidney	0.000762	1.276	CNN1,DLG1,FBLN1,HDAC6,MTOR,PBX1,SPARC	7
Cellular Movement,Hematological System Development and Function,Immune	Cellular infiltration by leukocytes	0.000768	0.263	ADA,AHR,ANXA2,APOA1,C3,CCN2,CD276,COMMD5,CRYAB,F2,GSDMD,HLA-A,IL1RN,	30
Cardiovascular Disease,Gastrointestinal Disease,Hematological Disease,Hep	Portal vein thrombosis	0.000775		F2,F5,SERPINC1	3
Respiratory Disease	Abnormal lung capacity	0.000775		DERL2,Gm15807/Hmgn5,SELENON	3
Carbohydrate Metabolism,Molecular Transport,Small Molecule Biochemistry	Quantity of proteoglycan	0.000775		CCN2,F2,TGFBR2	3
Cancer,Neurological Disease,Organismal Injury and Abnormalities	Central nervous system solid tumor	0.000793	-0.16	A2M,AAK1,ACAD8,ACADM,ACOT2,ACP2,ACP6,ACSL1,ACY3,ADA,ADAM10,AHR,AKR1	302
Amino Acid Metabolism,Small Molecule Biochemistry	Metabolism of amino acids	0.0008		ACAD8,ALDH4A1,BCAT1,BCKDHA,BCKDK,BLMH,CTH,FAH,GCSH,HIBADH,HIBCH,HSD	16
Cell Death and Survival	Apoptosis of central nervous system cells	0.000802	0.139	A2M,BCL2L1,CAPN1,Casp12,CCNB1,CREBBP,CRYAB,F2,FADD,HK2,LRPAP1,PRPH,R	14
Cardiovascular Disease,Hematological Disease,Organismal Injury and Abnorn	Thrombosis of artery	0.000812		ANXA2,CAPN1,F2,PRCP,SERPINC1,THBD	6
Developmental Disorder	Intrauterine growth retardation	0.000841		AGK,CDKN2B,F2,F5,PTGS1,RBPJ,SERPINC1	7
Tissue Development	Accumulation of cells	0.000842	-1.065	AHR,APOA1,C3,COL18A1,CRYAB,F2,FADD,GNAQ,HS2ST1,IFI16,IL1RN,ITGA2,ITGA6,L	24
Cancer,Neurological Disease,Organismal Injury and Abnormalities	Central nervous system cancer	0.000845		A2M,AAK1,ACAD8,ACADM,ACOT2,ACP2,ACP6,ACSL1,ACY3,ADA,ADAM10,AHR,AKR1	296
Cardiovascular System Development and Function,Cellular Development,Cell	Endothelial cell development	0.00085	-2.247	ANXA2,APOA1,C3,CAV2,CD276,CD9,COL18A1,COL1A1,COL1A2,DAB2,DLG1,ERAP1,F	26
Neurological Disease,Skeletal and Muscular Disorders	Neuromuscular disease	0.000853	0.555	A2M,ACADM,ADAM10,ALB,ALCAM,ALG2,ANXA2,ARL3,ARMCX3,ATP6AP2,BCL2L1,C3,	54
Cardiovascular System Development and Function	Morphology of vessel	0.000855		AHR,CNN1,COL3A1,COL5A1,CREBBP,CRYAB,CYP51A1,ENPP1,FBLN1,IL1RN,JMJD6,I	22
Organ Development,Organ Morphology,Skeletal and Muscular System Develo	Mass of skeletal muscle	0.000858		COL12A1,EFEMP1,IFRD1,MTOR,PUS1,Sacs,SELENON,STAT5A,USP19	9
Cell Death and Survival,Organismal Injury and Abnormalities	Cell death of epithelial cells	0.000859	0.06	AAK1,AHR,ARID1A,ATRX,BCL2L1,Casp12,CRYAB,CTH,DAB2,F2,FADD,FAH,GSDMD,H	34
Endocrine System Disorders,Metabolic Disease,Organismal Injury and Abnorr	Insulin resistance	0.000866	1.487	ACACA,ACAT2,ACSL1,AKT2,APOA1,C3,CLPP,COL5A2,CPE,CTHRC1,ENPP1,F2,LDLR,	24
Cellular Function and Maintenance,Molecular Transport	Flux of chloride	0.000869	-1	ADA,ARFGEF1,CLCN5,SLC12A2	4
Cancer,Organismal Injury and Abnormalities,Reproductive System Disease	Male genital neoplasm	0.000878	0	A2M,AAK1,ACAD8,ACADM,ACP6,ACY3,ADAM10,ADD3,AKT2,ALB,ALDH4A1,ANAPC5,A	179
Organismal Injury and Abnormalities,Tissue Morphology	Abnormal morphology of epithelial tissue	0.00088		ADA,AHR,CAV2,CCN2,COL18A1,CTHRC1,DLG1,FADD,FADS2,FAH,FBLN1,ITGA6,KRT	27
Cancer,Organismal Injury and Abnormalities,Reproductive System Disease	Malignant neoplasm of male genital organ	0.000889		A2M,AAK1,ACAD8,ACADM,ACP6,ACY3,ADAM10,ADD3,AKT2,ALB,ALDH4A1,ANAPC5,A	177
Metabolic Disease,Organismal Injury and Abnormalities,Renal and Urological	Aciduria	0.000896	1.387	BCKDHA,CLCN5,DHTKD1,FAH,HADH,MVK,PCCA,PCCB	8
Cell-To-Cell Signaling and Interaction,Hematological System Development an	Binding of professional phagocytic cells	0.000904	0.351	A2M,ADAM10,AKT2,ALCAM,APOA1,C3,F2,ITGA6,JMJD6,KNG1,ROCK1,SCARB1,STAT	16
Cell Cycle,Cell-To-Cell Signaling and Interaction,Cellular Growth and Prolifera	Contact growth inhibition of tumor cell lines	0.000921	-0.701	CTH,DDX58,IFI16,IFIH1,LTBP1,NME1,RSU1,SMARCE1,STAT6	9
Cardiovascular Disease,Organismal Injury and Abnormalities,Skeletal and Mu	Acute myocardial infarction	0.000937		APOA1,C3,CAMLG,F2,GSTM1,MME,PTGS1,SERPINC1,TF,TUBA1C	10
Inflammatory Response	Inflammation of secretory structure	0.000963	1.052	AHR,ALB,ANXA4,APOA1,F2,F5,MME,NINJ1,NMI,PTGS1,RIDA,SLC33A1,STXBP3,TGFB	14
Lipid Metabolism,Molecular Transport,Small Molecule Biochemistry	Concentration of fatty acid	0.000967	0.364	ACACA,ACOT2,ACSL1,AHR,BCL2L1,C3,ELOVL5,F2,FADS2,GNAQ,HADH,IL1RN,KNG1,	22
Developmental Disorder,Embryonic Development,Organismal Survival	Death of embryo	0.000975	0.577	ATP6V0C,Brd4,CHAF1A,DNAJB1,FADD,GPX4,HK2,HSBP1,INTS1,NBN,PES1,RBPJ	12
Cancer,Organismal Injury and Abnormalities	Embryonal tumor	0.000977		AKT2,ALCAM,ARID1A,ARMCX3,ATRX,BCL2L1,BCL2L13,C3,CDC27,CDKN2B,COL1A1,(	43
Infectious Diseases	HIV infection	0.000988	-1.717	ADA,ADAM10,ALB,ANXA2,APOA1,ARID1A,ARPC1A,ATP6V0C,BCL2L1,C3,CAPN6,CAV	42
Organismal Development	Length of absolute anatomical region	0.000989		ARID1A,CCN2,COL1A2,IFIH1,ITGA2,LAMB2,P3H1,S100A4,STAT6	9
Organ Development,Skeletal and Muscular System Development and Functio	Function of skeletal muscle	0.00101		AKT2,CAV2,COL12A1,EFEMP1,IFRD1,MTOR,PUS1,Sacs,SELENON,STAT5A,USP19	11
Cancer,Gastrointestinal Disease,Organismal Injury and Abnormalities	Colon carcinoma	0.00101		A2M,ACADM,ACP6,ACSL1,ADA,ADD3,ALB,ANAPC5,ANXA11,ANXA4,ARAF,ARFGAP3,	216
Skeletal and Muscular Disorders	Abnormality of lower limb	0.00102	0.816	BPNT2,CCN2,CD276,COL12A1,CTH,CYP51A1,DHTKD1,FDPS,HDAC6,HELLS,IL1RN,LD	15
Cardiovascular Disease,Developmental Disorder,Organismal Injury and Abnor	Hypoplasia of heart ventricle	0.00103	0	CYP51A1,FADD,MECOM,PBRM1,RBPJ,TAB1	6
Developmental Disorder,Hereditary Disorder,Metabolic Disease,Organismal In	Inborn error of carbohydrate metabolism	0.00103		ALG2,ATP6AP2,C3,CCDC115,COG5,CTSA,FCSK,FUCA1,GALE,GLB1,PTGS1,TMEM16	12
Cardiovascular Disease,Organismal Injury and Abnormalities	Coronary artery disease	0.00103		ACAT2,ADA,APOA1,COL5A2,F2,FADS1,HBA1/HBA2,KDM1A,LDLR,LOX,LRPAP1,MECO	17
Tissue Development	Development of epithelial tissue	0.00104	-2.192	AHR,ANXA2,APOA1,ATRX,C3,CAV2,CCN2,CD276,CD9,COL18A1,COL1A1,COL1A2,DA	35
Neurological Disease	Degeneration of nervous system	0.00105	2.137	BCL2L1,C3,CAPN1,CREBBP,DHTKD1,F2,FUCA1,GLB1,GPX4,HDAC6,IL1RN,LRPAP1,M	21
Metabolic Disease,Organismal Injury and Abnormalities	Disorder of lipid metabolism	0.00105	-0.125	ACAD8,ACADM,ACAT2,AKT2,APOA1,CAMLG,CCN2,CPT2,DAB2,F2,GLA,GM2A,HSD17	20
Cancer,Organismal Injury and Abnormalities,Reproductive System Disease	Ductal breast carcinoma	0.00106		AAK1,AKT2,ANAPC5,ARFGEF1,ARID1A,ASH2L,ATRX,BCL2L1,C3,CAPN1,CD109,CDC2	48
Cardiovascular System Development and Function,Cell-To-Cell Signaling and	Adhesion of vascular endothelial cells	0.00106	-0.665	ADAM10,ALCAM,CD63,F2,IGFBP7,ITGA6,RICTOR,SPARC,STAT6,STX4	10
Cancer,Neurological Disease,Organismal Injury and Abnormalities	Glioma cancer	0.00107		A2M,AAK1,ACAD8,ACADM,ACOT2,ACP2,ACP6,ACSL1,ACY3,ADA,ADAM10,AHR,AKR1	291
Cancer,Organismal Injury and Abnormalities,Reproductive System Disease	Uterine carcinoma	0.00108		AAK1,ACAD8,ACADM,ACSL1,ADD3,AGK,AHR,AKT2,ALB,ALDH4A1,ALG2,ANKH,ANXA	215
Cancer,Organismal Injury and Abnormalities,Reproductive System Disease	Endometrial adenocarcinoma	0.00108		AAK1,ACAD8,ACADM,ACSL1,ADD3,AGK,AHR,AKT2,ALB,ALDH4A1,ALG2,ANKH,ANXA	192

Cancer,Organismal Injury and Abnormalities	Melanoma	0.00108	1.302	A2M,AAK1,ACADM,ACOT2,ACP2,ACP6,ACY3,ADA,ADAM10,ADD3,AGK,AHR,AKT1S1,A	358
Lipid Metabolism,Small Molecule Biochemistry	Synthesis of thromboxane A2	0.00108		ACACA,F2,LDLR,PTGS1	4
Cardiovascular Disease,Organismal Injury and Abnormalities,Tissue Morpholo	Quantity of vascular lesion	0.00108		APOA1,LDLR,LOX,STAT6	4
Cardiovascular Disease,Organismal Injury and Abnormalities	Fibrosis of blood vessel	0.00108		AHR,BLMH,IL1RN,LDLR	4
Hair and Skin Development and Function	Tensile strength of skin	0.00108	-1.969	COL5A1,COL5A2,LOX,P3H4	4
Cardiovascular Disease	Fatty lesion	0.00108		LDLR,PON3,STAT6,TGFBR2	4
Developmental Disorder,Hereditary Disorder,Ophthalmic Disease,Organismal	Autosomal recessive congenital cataract	0.00108		AGK,MSMO1,PIK3C2A,SLC33A1	4
Hematological Disease,Metabolic Disease,Organismal Injury and Abnormalitie	Acidemia	0.00108		ALDH4A1,GCSH,PCCA,PCCB	4
Infectious Diseases	Replication of Sendai virus	0.00108	0.849	CNOT7,HDAC6,STING1,UBE3C	4
Inflammatory Response	Inflammation of body cavity	0.00111	0.625	ADA,AHR,AKT2,ALB,ANXA4,APOA1,ARID1A,ATRX,BCL2L1,C3,CAMLG,CHCHD2,CLCN	60
Infectious Diseases	Sexually transmitted disease	0.00111		ALB,BCL2L1,C3,CAMLG,CHCHD2,CYP51A1,DDX58,GCSH,HBA1/HBA2,MTOR,PTGS1,S	13
Cancer,Gastrointestinal Disease,Hepatic System Disease,Organismal Injury a	Hepatobiliary system cancer	0.00114	-0.053	A2M,ACSL1,ADA,ADAM10,AGK,AHR,AKT2,ALB,ALCAM,ALDH4A1,ANAPC5,ANXA2,APC	221
Hematological Disease	Hypercoagulation	0.00114		F5,PTGS1,SERPINC1	3
Cardiovascular Disease,Organismal Injury and Abnormalities,Skeletal and Mu	Recurrent myocardial infarction	0.00114		F2,PTGS1,SERPINC1	3
Dermatological Diseases and Conditions,Organismal Injury and Abnormalities	Fibrosis of dermis	0.00114		AHR,COL1A1,TGFBR2	3
Cellular Assembly and Organization,DNA Replication, Recombination, and Re	Quantity of PML nuclear bodies	0.00114		GOLGA3,NBN,STAT5A	3
Organ Morphology	Enlargement of airspace	0.00114		ADA,MTOR,SERPINB1	3
Lipid Metabolism,Small Molecule Biochemistry	Hydrolysis of cholesterol ester	0.00114		APOA1,LDLR,SCARB1	3
Lipid Metabolism,Molecular Transport,Small Molecule Biochemistry	Influx of cholesterol	0.00114		APOA1,LDLR,SCARB1	3
Developmental Disorder,Hereditary Disorder,Metabolic Disease,Neurological I	Susceptibility to Alzheimer disease	0.00114		A2M,ADAM10,TF	3
Cardiovascular Disease,Cardiovascular System Development and Function,O	Abnormal morphology of aorta	0.00115		COL3A1,COL5A1,LOX,LTBP1,PLOD1,TAB1	6
Lipid Metabolism,Small Molecule Biochemistry	Catabolism of lipid	0.00116	-2.211	ACSL1,AHR,ECI2,GLA,GLB1,GM2A,LDLR,PCCA,PCCB,SCARB1,SERPINF1,SMPD4,TB	13
Cardiovascular Disease,Cardiovascular System Development and Function	Abnormal morphology of cardiovascular sy	0.00117		ACADM,ACSL1,ADA,ADAM10,AHR,ARAF,BCL2L1,CCN2,CDK9,CDS2,COL18A1,COL3A	48
Infectious Diseases	Infection of tumor cell lines	0.00118	-1.878	ADAM10,ARPC1A,ATP6AP2,CAMK1,CAPN6,CAV2,CHCHD2,CNOT7,COG5,DAB2,DDX5	32
Lipid Metabolism,Molecular Transport,Small Molecule Biochemistry	Concentration of phospholipid	0.00119	1.777	ACACA,ACP6,ADA,APOA1,CDS2,F2,GLB1,GNAQ,KNG1,LDLR,MBOAT7,MTOR,PIK3C2	16
Cellular Movement	Cell movement of blood cells	0.0012	-0.285	ADA,ADAM10,AHR,AKT2,ALB,ALCAM,ANXA2,APOA1,C3,CAMK1,CCN2,CD276,CD63,C	58
